# System analysis based on Anoikis-related genes identifies MAPK1 as a novel therapy target for osteosarcoma with neoadjuvant chemotherapy

**DOI:** 10.1186/s12891-024-07547-2

**Published:** 2024-06-04

**Authors:** Zhouwei Wu, Jiapei Yu, Tao Han, Yiting Tu, Fang Su, Shi Li, Yixing Huang

**Affiliations:** 1 Department of Orthopedics, the Second Affiliated Hospital, Yuying Children’s Hospital of Wenzhou Medical University, Wenzhou, 325000 China; 2 Department of Orthopedics, the Shaoxing People’s Hospital (Shaoxing Hospital of Zhejiang University), Shaoxing, 312000 China; 3 Key Laboratory of Orthopedics of Zhejiang Province, Wenzhou, 325000 China; 4 Department of Orthopaedics, The Second Affiliated Hospital, Yuying Children’s Hospital of Wenzhou Medical University, 109 West Xueyuan Road, Wenzhou, 325027 Zhejiang Province China

**Keywords:** Anoikis, MAPK1, Osteosarcoma, Prognostic model, Drug resistance

## Abstract

**Background:**

Osteosarcoma (OS) is the most common bone malignant tumor in children, and its prognosis is often poor. Anoikis is a unique mode of cell death.However, the effects of Anoikis in OS remain unexplored.

**Method:**

Differential analysis of Anoikis-related genes was performed based on the metastatic and non-metastatic groups. Then LASSO logistic regression and SVM-RFE algorithms were applied to screen out the characteristic genes. Later, Univariate and multivariate Cox regression was conducted to identify prognostic genes and further develop the Anoikis-based risk score. In addition, correlation analysis was performed to analyze the relationship between tumor microenvironment, drug sensitivity, and prognostic models.

**Results:**

We established novel Anoikis-related subgroups and developed a prognostic model based on three Anoikis-related genes (MAPK1, MYC, and EDIL3). The survival and ROC analysis results showed that the prognostic model was reliable. Besides, the results of single-cell sequencing analysis suggested that the three prognostic genes were closely related to immune cell infiltration. Subsequently, aberrant expression of two prognostic genes was identified in osteosarcoma cells. Nilotinib can promote the apoptosis of osteosarcoma cells and down-regulate the expression of MAPK1.

**Conclusions:**

We developed a novel Anoikis-related risk score model, which can assist clinicians in evaluating the prognosis of osteosarcoma patients in clinical practice. Analysis of the tumor immune microenvironment and chemotherapeutic drug sensitivity can provide necessary insights into subsequent mechanisms. MAPK1 may be a valuable therapeutic target for neoadjuvant chemotherapy in osteosarcoma.

**Supplementary Information:**

The online version contains supplementary material available at 10.1186/s12891-024-07547-2.

## Introduction

Osteosarcoma is a threatening bone malignancy that is common in adolescents and children [1]. Current treatments include surgical resection and pre-and postoperative chemotherapy, with five-year survival rates of up to 70% for non-metastatic osteosarcoma patients [2]. Osteosarcoma, a type of bone cancer, is known for its resistance to conventional chemotherapy treatments, which can have a significant impact on the patient’s prognosis [3]. Our aim is to identify the genes that hinder patients’ response to treatment and find suitable chemotherapy drugs. This would help us discover more effective targeted therapies that can overcome treatment resistance and serve as prognostic indicators for patients with osteosarcoma [4].

Anoikis is a type of programmed cell death that occurs when cells separate from the correct extracellular matrix, thereby disrupting integrin ligation. It is a critical mechanism that prevents dysplastic cells from growing abnormally or attaching to inappropriate substrates [5]. Anoikis resistance was found to be an important mechanism of cancer growth, invasion, and metastasis [6]. Numerous studies have shown that Anoikis plays a significant role in the occurrence and development of cancer. CPT1A promotes colorectal cancer cell metastasis by inhibiting Anoikis [7]. In addition, the CamKK2-AMPK signaling pathway leads to the metastasis and invasion of LKB1-deficient lung cancer [8]. However, the correlation between Anoikis and the prognosis of osteosarcoma still needs to be elucidated.

In the present study, we constructed a new model of risk score based on the Anoikis-related genes in osteosarcoma. We further explored the predictive value of these genes and investigated the association between Anoikis and the tumor immune microenvironment. We also focused on the analysis of related genes and chemotherapeutic susceptibility. Our findings will provide a novel perspective for predicting individualized survival and better treatment of osteosarcoma patients.

## Materials and methods

### Data collection

The gene sets information, and corresponding clinical data of 84 osteosarcoma patients(63 nonmetastasis and 21 metastasis) were obtained from the Therapeutically Applicable Research to Generate Effective Treatments (TARGET) database. Clinical information included gender, age, diagnosis, metastasis, survival time, status, and follow-up. We downloaded another dataset of 53 osteosarcoma patients from the Gene Expression Omnibus (GEO) database (GSE21257). We retrieved the 639 Anoikis-related gene lists from the Genecards database (https://www.genecards.org/) and Harmonizome portals (https://maayanlab.cloud/Harmonizome/) [9] (Shown in Table S1 and Table S2). The expression data were normalized to fragment per kilobase million (FPKM) values. The calculation of FPKM for gene i uses the following formula [10]:

$$FPK{M_i} = {{{q_i}} \over {{{{l_i}} \over {{{10}^3}}}*{{\sum\nolimits_j {{q_j}} } \over {{{10}^6}}}}} = {{{q_i}} \over {{l_i}*\sum\nolimits_j {{q_j}} }}*{10^9}$$

*q*_*i*_ are raw read or fragment counts, $l_{i}$ is feature length, $\sum\limits_j {{q_j}}$ and corresponds to the total number of mapped reads or fragments.

### Identification of differentially expressed Anoikis-related genes

We applied the “limma” R package to find different expressions of Anoikis-related genes. Then we used the “heatmap” R package to accomplish the heatmaps of differentially expressed genes (DEGs).

### Machine learning algorithm

LASSO logistic regression and support vector machine recursive feature elimination (SVM-RFE) algorithms were used to screen out characteristic genes. LASSO logistic regression was performed based on the “glmnet” R package [11]. SVM analysis, a machine learning method that depends on the “e1071” R package, can find the best variables by deleting the feature vectors generated by SVM [12].

### Development and validation of the Anoikis-related genes (ARGS) prognostic model

Univariable Cox regression analysis was used to assess the prognostic value of each Anoikis-related DEG in the TARGET cohort. Those genes with *p* < 0.05 were chosen for further investigation, and multivariate Cox regression analysis was applied to shrink the potential genes and build the prognostic prediction model. The formula calculating for risk score was “Riskscore = Gene A∗Coef A + Gene B ∗ Coef B + … + Gene N ∗ Coef N” [13]. High- and low-risk score groups were divided according to the median Anoikis-related risk score of the training cohort. And the Kaplan-Meier analysis was conducted to compare survival possibility and overall survival time between the high- and low-risk groups. The area under the curve (AUC) was calculated to assess the sensitivity and specificity of the risk score system. Then, we used the univariate and multivariate Cox regression analysis to validate the independence of our prognosis model. Finally, we underwent external validation for the Anoikis-related risk score system in the GEO cohort.

### Development of a nomogram

We applied the univariate and multivariable Cox regression analysis (“survival” R package) to assess the risk score combined with clinical information (age, gender, and metastatic status) in the TARGET cohort. Based on the results of Cox regression analysis, a nomogram was presented to predict the prognosis of osteosarcoma patients. Besides, the calibration curve was shown to evaluate the nomogram’s performance.

### Gene set enrichment analysis (GSEA)

Gene set enrichment analyses (GSEA) software was used to identify the enriched pathways between the two cluster groups based on the curated genesets (go.v7.4.symbols.gmt and kegg.v7.4.symbols.gmt) [14].

### Immune Cell Infiltration and Immune function analysis

We used the “GSVA” R package to quantify immune cell infiltration and immune function in the TARGET cohort [15]. Subsequently, we analyzed the correlation between the two clusters and different enriched pathways.

### Drug susceptibility analysis

The “OncoPredict” R package was applied to predict in vivo drug responses in cancer patients [16]. OncoPredict fits the gene expression profile of tissues to the half-maximal inhibitory concentration (IC50) of the cancer cell lines to drugs downloaded from Genomics of Drug Sensitivity in Cancer (GDSC; https://www.cancerrxgene.org/) and the gene expression profile of cancer lines from the Broad Institute Cancer Cell Line Encyclopedia (CCLE; https://portals.broadinstitute.org/ccle_legacy/home). 198 drugs were calculated in total, and the sensitivity of the drugs was analyzed using unpaired t-tests. *p* < 0.05 was considered statistically significant.

In addition, we downloaded the NCI-60 human cancer cell lines from the CellMiner database (https://discover.nci.nih.gov/cellminer) [17]. Pearson correlation analysis was applied to assess the relationship between MAPK1 and chemosensitivity.

### Single-cell sequencing analysis

The Tumor Immune Single Cell Hub (TISCH, http://tisch.comp-genomics.org) database is a comprehensive website that can realize the visualization of the tumor immune microenvironment [18]. Gene expression data were downloaded from the GEO database (GSE162454). All data were uniformly processed with a standardized cell-type annotation and differential expression analysis.

### Cell lines and cultures

One human osteoblast cell line (hFOB1.19) and two human osteosarcoma cell lines (U-2OS and MG-63) were obtained from the National Collection of Authenticated Cell Cultures (Shanghai, China). Dulbecco’s modified Eagle’s medium (DMEM, Gibco) contains 1% penicillin/streptomycin (Thermo Fisher Scientific, United States) and 10% fetal bovine serum (FBS, Gibco). We cultured human osteoblast cells in the medium at 34℃ with 5% CO2, and the osteosarcoma cells at 37℃ with 5% CO2.

### Cell viability assay

Following the manufacturer’s protocol, U-2OS and MG-63 cell survival was assessed via the CCK-8 kit. About 5 × 10³ cells were extracted from U-2OS and MG-63 cell suspensions, respectively, and incubated at 37℃ and 5% CO2 for 24 h in each well of the 96-well plate. The cells were then treated with different concentrations of Nilotinib (0, 10, 20, and 30 µM). Cells were washed using phosphate-buffered saline (PBS) after incubation. 100 µl of DMEM containing 10 µl CCK-8 solution was added to each well, then the mixture was incubated for 2–4 h. The absorbance of the wells was measured using a microplate reader at 450 nm.

### Western blotting

After cell treatments, whole-cell proteins from hFOB, U-2OS, and MG-63 cells were extracted using commercial kits (Beyotime) according to the manufacturer’s instructions. The protein quantification was determined using the BCA protein assay kit. Then 20 ng of protein from each group was resolved via sodium dodecyl sulfate-polyacrylamide gel electrophoresis (SDS PAGE) and transferred to a PVDF membrane. Membranes were blocked with 5% non-fat milk. Then the blots were cut prior to incubated with primary antibodies against MYC (1:1000), MAPK1 (1:1000), GAPDH (1:1000), Bcl-2 (1:1000), Bax (1:1000), and CASP3 (1:1000) overnight at 4℃. Membranes were washed and further incubated with the respective secondary antibodies. Electrochemiluminescence plus reagent (Invitrogen) was used to detect the bands. Blots were imaged and quantified using Image Lab 3.0 software.

### TUNEL staining

To measure U-2OS apoptosis under various 24 h treatments, TUNEL labeling was used. After being fixed for 15 min at room temperature in 4% paraformaldehyde (PFA), U-2OS were rinsed with PBS and permeabilized for 3 min on ice using 0.1% Triton X-100 buffer. Following observation under a confocal microscope, apoptotic U-2OS were stained with the TUNEL staining kit reagents, and the nuclei were counterstained with DAPI for 10 min. Apoptotic U-2OS were then counted and analyzed.

### RNA extraction and real-time PCR analysis (RT-PCR)

Following the manufacturer’s instructions, total RNA was isolated from the hFOB, U-2OS, and MG-63 using TRIzol (Invitrogen), and then reverse-transcribed into cDNA (MBI Fermentas, Germany), and the RT-PCR reaction was performed in the RT-PCR system (Bio-Rad Laboratories, CA, USA), according to the operating instruction. The expression levels of relative genes were calculated via a comparative quantification method (2 − ΔΔCt formula) and were normalized to internal control, GAPDH. MYC and MAPK1 primers were designed with the NCBI Primer-Blast Tool, and they are presented below:

### MYC

- (F) 5’ GGCTCCTGGCAAAAGGTCA 3’,
- (R) 5’ CTGCGTAGTTGTGCTGATGT 3’.

### MAPK1

c. (F) 5’ TACACCAACCTCTCGTACATCG 3’,
d. (R) 5’ CATGTCTGAAGCGCAGTAAGATT 3’.

### Statistical analysis

All statistical analysis was conducted by using R software (Version:3.6.1) and GraphPad Prism (Version:7.00). Comparisons between two independent groups were applied using a two-tailed, unpaired t-test. Two-way analysis of variance (ANOVA) with Tukey’s multiple comparisons test was applied to analyze differences among three or more groups when the data were normally distributed. What’s more, nonparametric Mann-Whitney U tests were used for groups if the data were not normally distributed. P value < 0.05 was considered statistically significant.

## Results

### Identification of characteristic genes

**Fig. 1:**
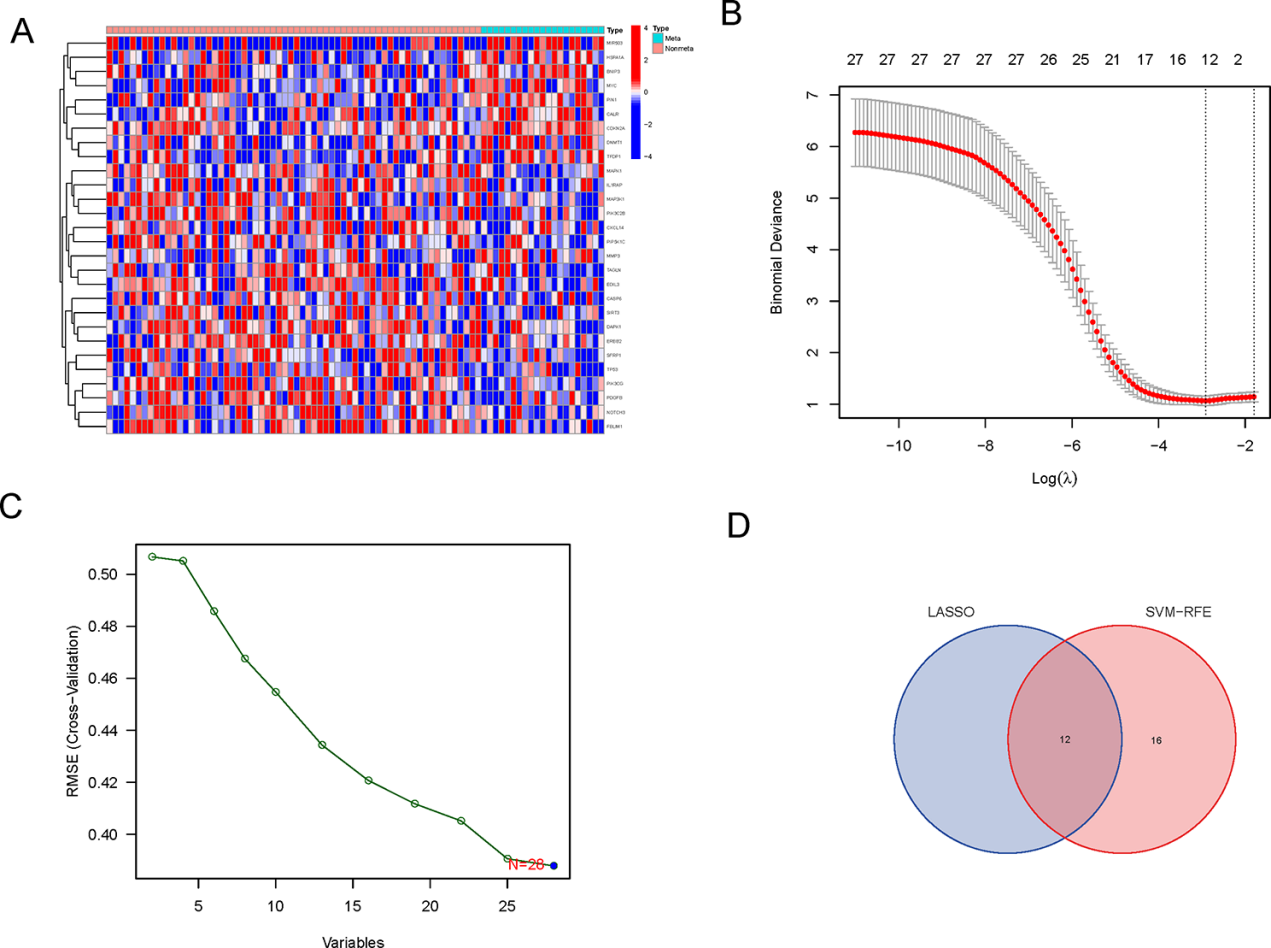
Identification of anoikis-related gene signature. (**A**) A heatmap (blue: low expression level; red: high expression level) of genes between the metaststic and the non-metaststic tumor tissues. (**B**) LASSO coefficient profiles. (**C**) SVM- RFE algorithm. (**D**) Twelve characteristic genes were selected between LASSO and SVM- RFE algorithms

The 639 Anoikis-related gene expression levels were compared in the TARGET database from metastatic and non-metastatic tissues. We identified 28 DEGs (all *P* < 0.05). The RNA levels of these Anoikis-related genes are shown in Fig. 1A (red: high expression level; blue: low expression level). LASSO logistic regression and SVM-RFE algorithms were applied to screen out the characteristic genes related to metastasis (Fig. 1B, C). Finally, we ended up with 12 genes which were selected between LASSO and SVM- RFE algorithms for further research., including MAPK1, ERBB2, CALR, PIK3CG, MYC, SIRT3, PIN1, CASP6, FBLIM1, PIP5K1C, DNMT1, and CXCL14 (Fig. 1D).

### Cancer classification based on the DEGs

**Fig. 2:**
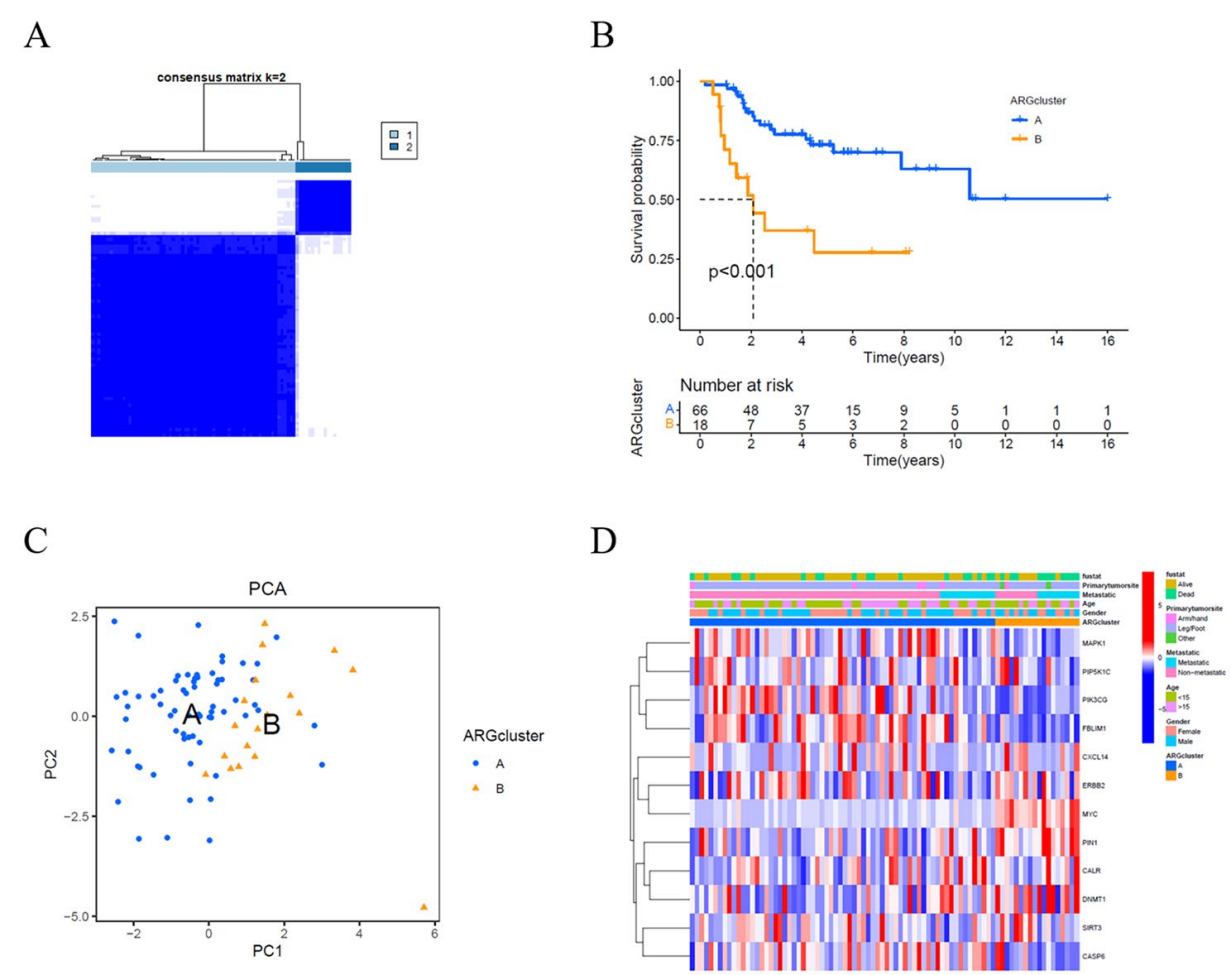
Tumor classification based on DEGs. (**A**) 84 osteosarcoma patients were grouped into two clusters via the consensus clustering matrix (k = 2). (**B**) Kaplan–Meier overall survival curves for the two clusters. (**C**) PCA plot. (**D**) A heatmap (blue: low expression level; red: high expression level)

We further investigated the association between the expression level of 12 Anoikis-related DEGs and osteosarcoma. Hence, we performed a consensus clustering analysis with 84 osteosarcoma patients. The intragroup correlations decreased as the clustering variable (k) increased from 2 to 10. when k = 2, the 84 osteosarcoma patients could be well divided into two clusters (Fig. 2A). We compared survival rates among the two clusters and found significant differences among these clusters’ patients (*p* < 0.01, Fig. 2B). Then the principal components analysis (PCA) results indicated that patients separated into A and B groups had significantly different discrimination (Fig. 2C). The relationship between the expression of these genes and the clinical characteristics, including gender, age (< 15 or > 15 years), metastasis status (metastatic or non-metastatic), and primary tumor site (Arm/hand Leg/Foot and other), is displayed in a heatmap (Fig. 2D).

#### Immune status and tumor microenvironment (TME)

**Fig. 3:**
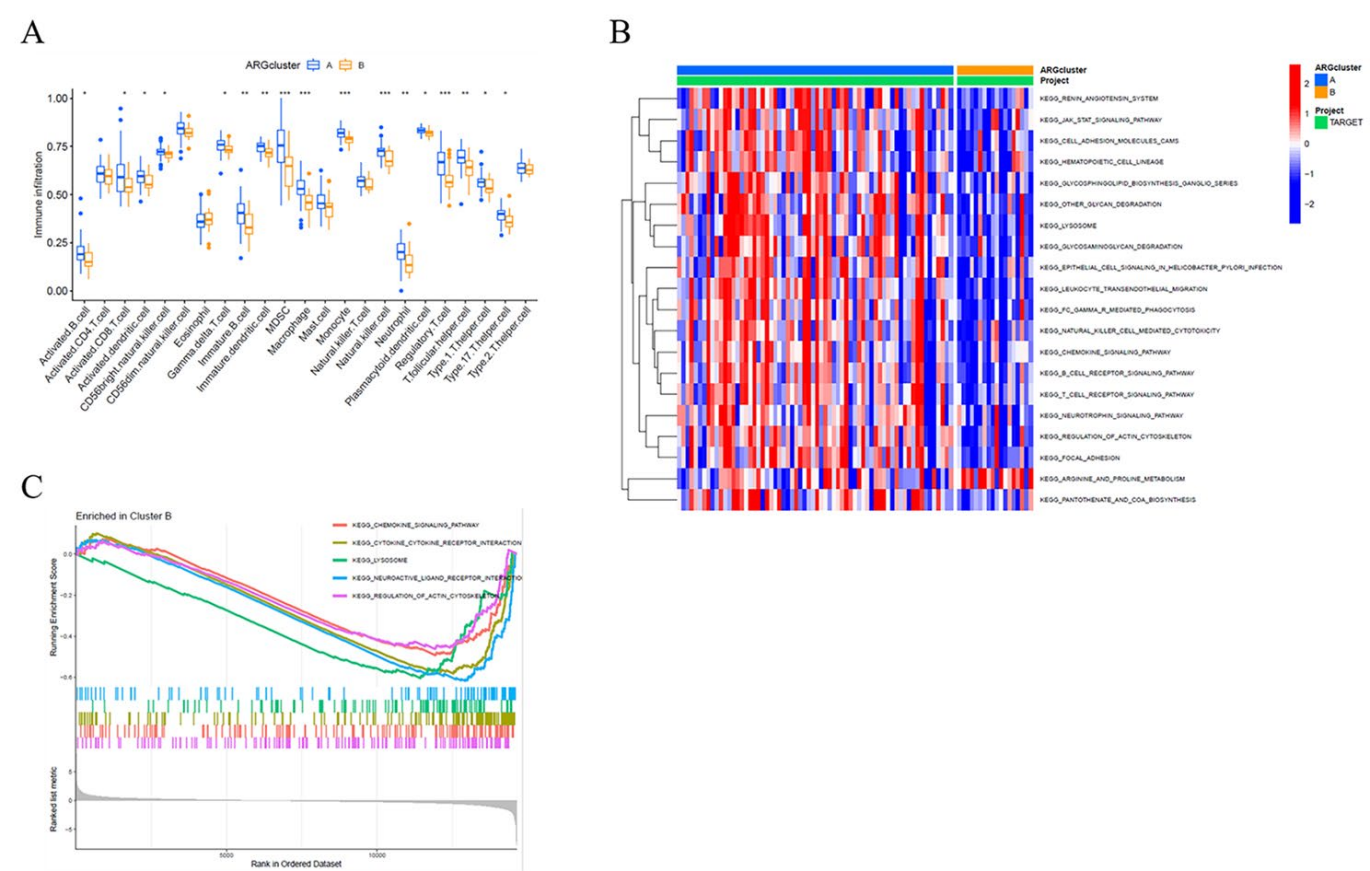
Immune status and tumor microenvironment. (**A**) The ssGSEA analysis for immune cells between two clusters. (**B**) The GSVA (Gene Set Variation Analysis) for KEGG pathways between two clusters. (**C**) GSEA (Gene set enrichment analysis) in B cluster (low survival group)

To study the TME differences between the two clusters, we further used ssGSEA to evaluate the enrichment scores of 23 types of immune cells. Several immune cell infiltrates were significantly enriched in group A, such as Macrophages, CD8 T cells, activated NK cells, and others (Fig. 3A). Gene Set Variation Analysis (GSVA) revealed that the following pathways were significantly activated in group A: JAK/STAT signaling pathway, chemokine signaling pathway, and others. Interestingly, Arginine and Proline Metabolism pathways were much more active in group B (Fig. 3B, C).

### Development of a prognostic model in the TARGET cohort

**Fig. 4:**
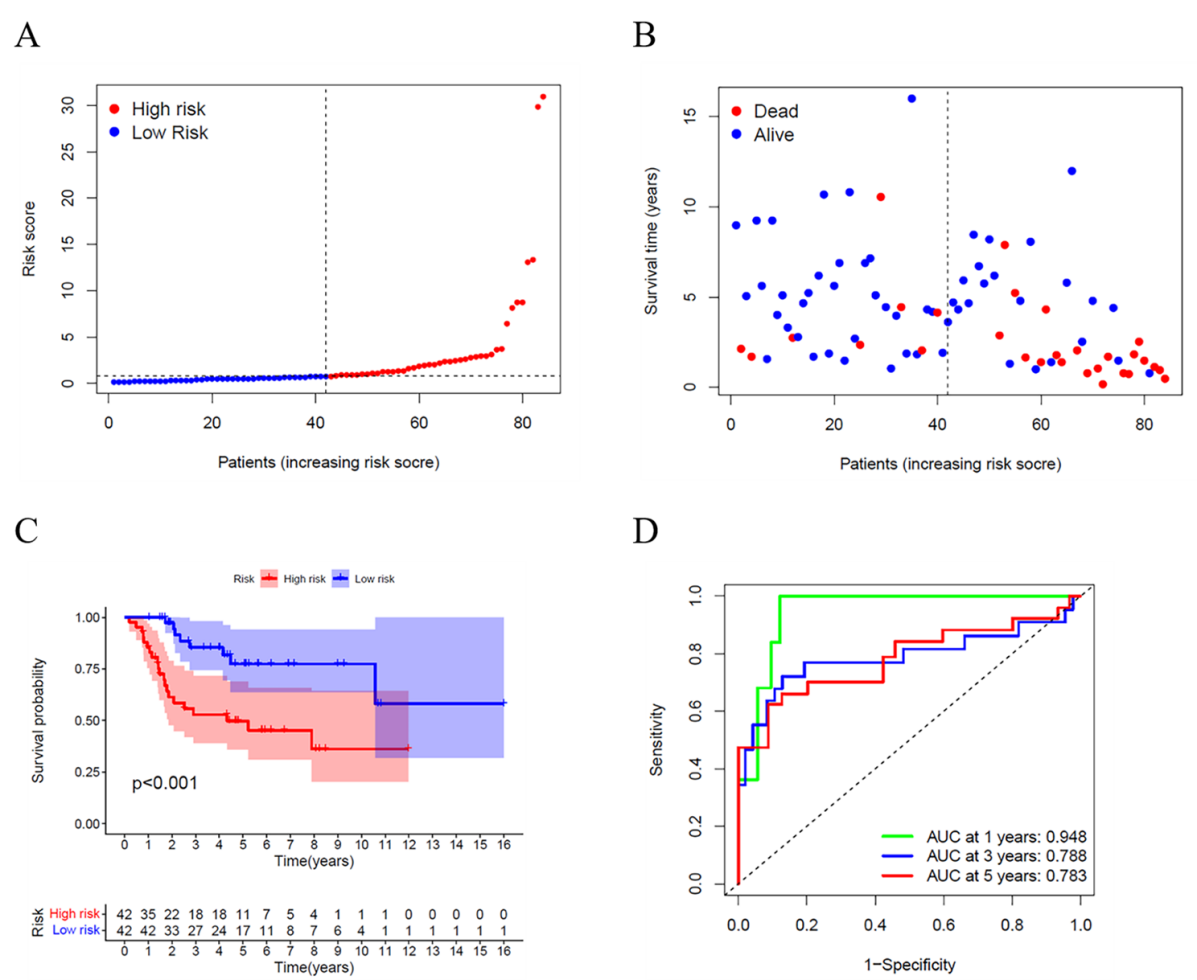
Construction of risk signature in the TARGET cohort. (**A**) Distribution of patients based on the risk score. (**B**) The survival status for each patient (left side of the dotted line: low-risk population; right side of the dotted line: high-risk population). (**C**) Kaplan–Meier curves for the overall survival of patients between the high- and low-risk groups. (**D**) ROC curves

A total of 84 osteosarcoma specimens were matched to corresponding patients with complete survival information. Univariate Cox regression analysis was used to initially screen genes associated with survival. The 4 genes (MAPK1, PIK3CG, MYC, EDIL3) that met the criteria of *P* < 0.05 were further analyzed. Then multivariate Cox regression analysis was performed, and a three-gene signature was constructed. The risk score was calculated as follows: risk score = (-0.884* MAPK1 exp.) + (0.656* MYC exp.) + (-0.772* EDIL3 exp.). According to the median risk score, we divided 84 osteosarcoma patients into high- and low-risk subgroups (Fig. 4A). High-risk patients had more deaths and shorter survival times than low-risk patients (Fig. 4B). The Kaplan-Meier curve showed that overall survival time and possibility were significantly lower in the high-risk group (Fig. 4C, *P* < 0.001). The value of the area under the curve was 0.948 for 1-year, 0.788 for 3-year, and 0.783 for 5-year survival prediction (Fig. 4D).

#### External validation of risk score in a GEO cohort

**Fig. 5:**
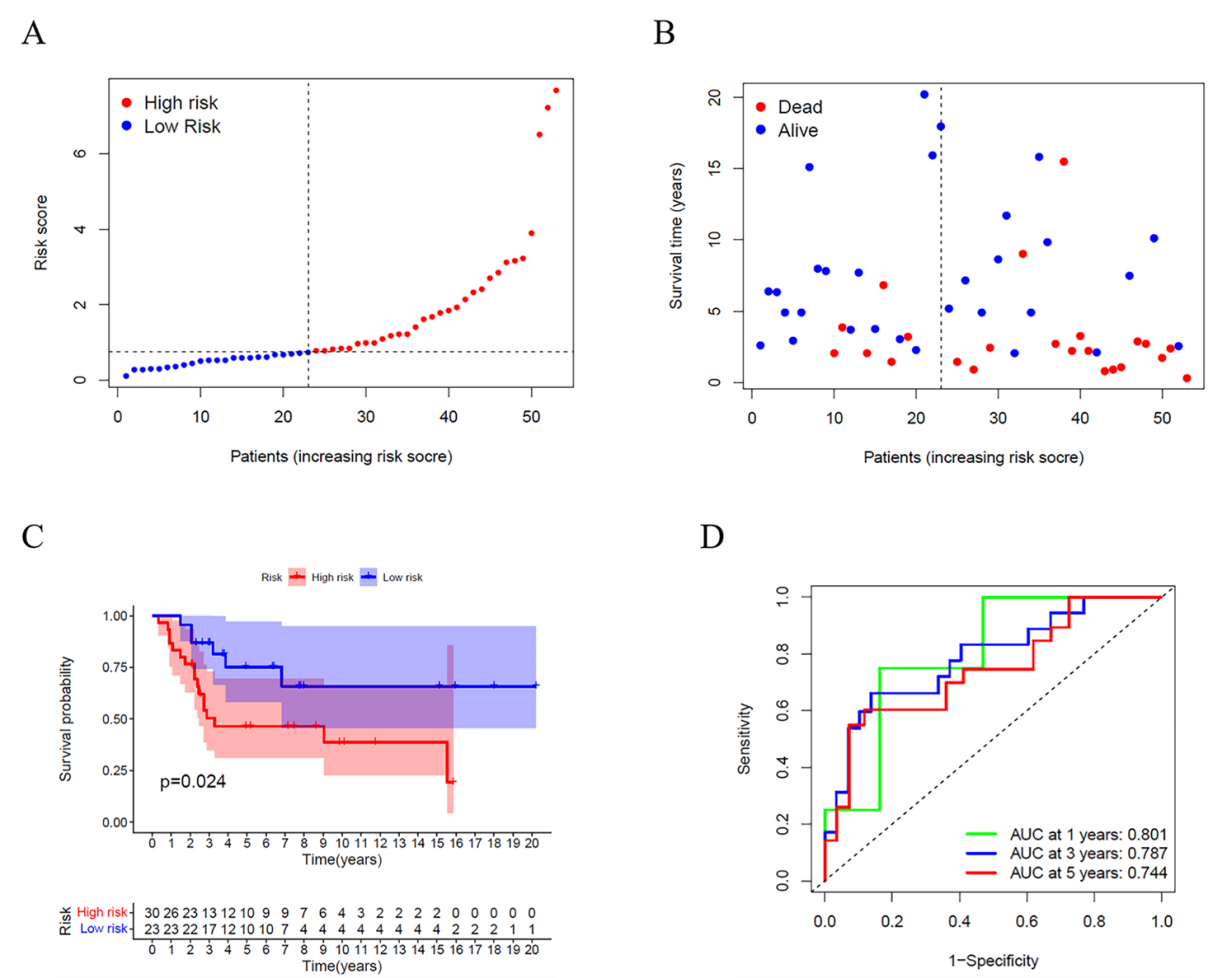
Validation of the risk model in the GSE21257. (**A**) Distribution of patients in the GSE21257 based on the median risk score of the TARGET cohort. (**B**) The survival status for each patient. (**C**) Kaplan–Meier curves. (**D**) Time-dependent ROC curves for osteosarcoma

53 osteosarcoma patients from a GEO cohort (GSE21257) were extracted as the external validation set. Based on the median risk score of the TARGET model, 30 patients were regarded as the high-risk group, while the other 23 were at low risk (Fig. 5A). The risk scores, survival time, and survival status of patients are shown in Fig. 5B. High-risk patients had higher mortality and shorter overall survival time. Besides, the Kaplan-Meier curve results also showed a lower survival possibility in the high-risk group (Fig. 5C, *P* = 0.024). AUC values of external validation also showed an optimistic prediction, and the AUC was 0.801 for 1 year, 0.787 for 3 years, and 0.744 for 5 years (Fig. 5D).

#### Independent prognostic value of the risk model

**Fig. 6:**
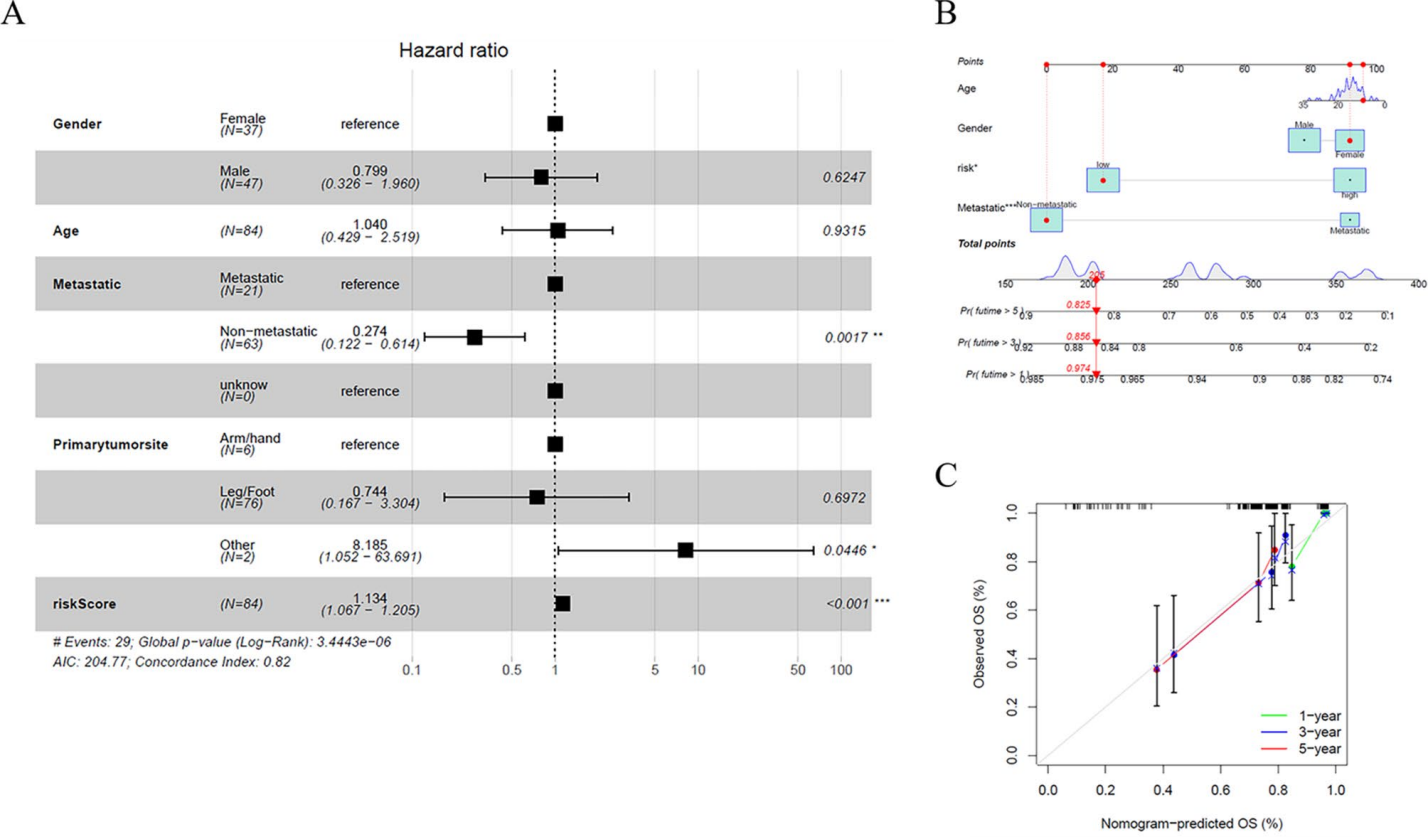
Construction of the predictive model. (**A**) Independence detection of the constructed risk prediction model. (**B**) A prognostic model to predict overall survival in the TARGET cohort. (**C**) Calibration curves of the OS nomogram model

Univariate and multivariable Cox regression models were conducted to assess independent prognostic factors of the gene-based risk score and clinical characteristics. In the TARGET cohort, univariate Cox analysis showed the metastatic status, primary tumor site, and risk score were significantly associated with prognosis (Fig. 6A).

#### Nomogram

According to the prognostic model and clinical factors (age, gender, and metastatic status), we developed a risk estimation nomogram in the TARGET cohort (Fig. 6B). 1-, 3-, and 5-year calibration curves showed that the nomogram consistently predicted the survival rate (Fig. 6C).

### Immune microenvironment analysis

**Fig. 7:**
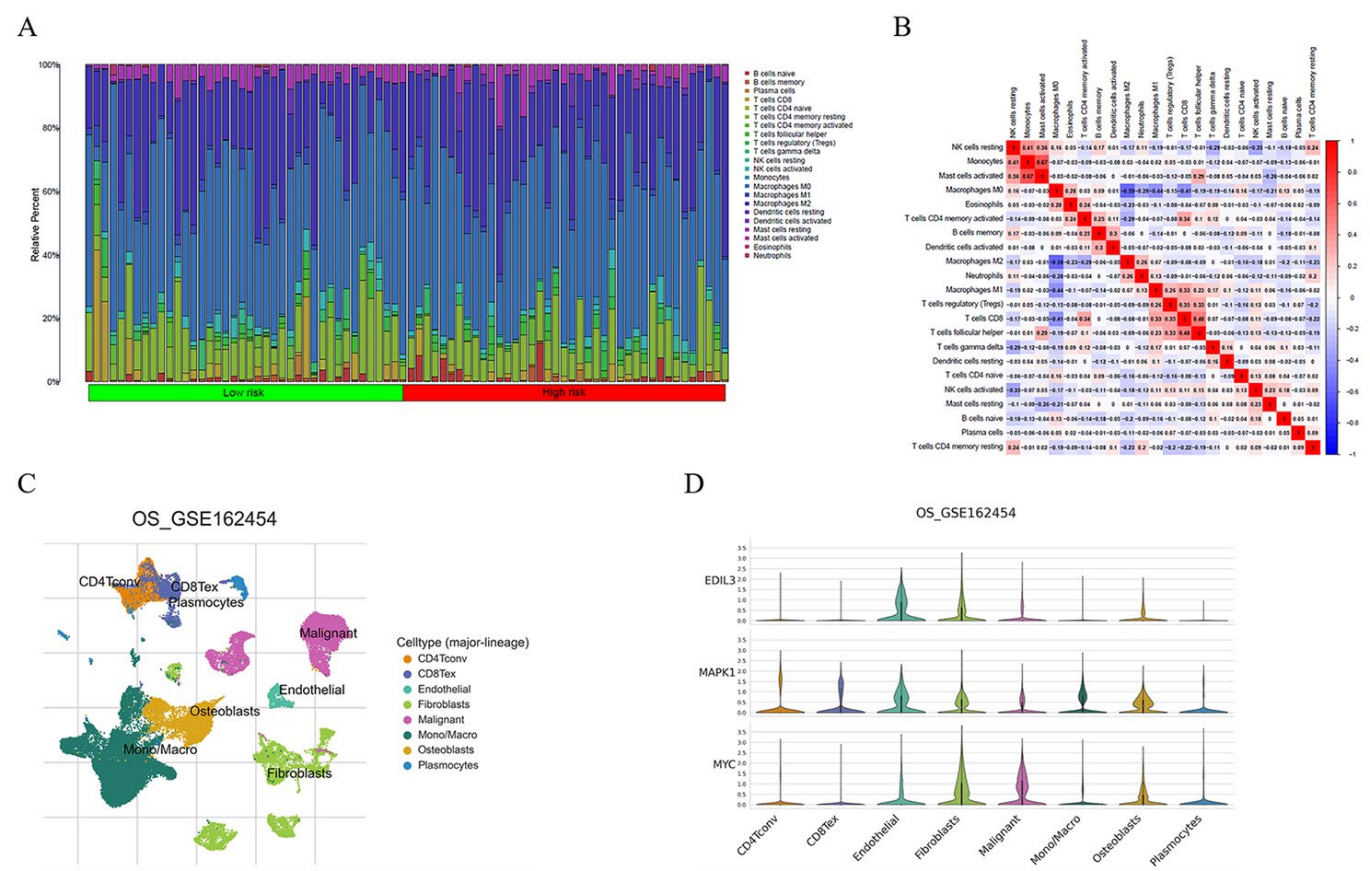
Immune microenvironment. (**A**) The immune infiltration of 22 immune cells between low- and high-risk group. (**B**) An immunocyte related heatmap. (**C**, **D**) single-cell cluster in TISCH database

The immune infiltration of 22 immune cells was investigated by the CIBERSORT algorithm (Fig. 7A). As shown in Fig. 7B, there were apparent correlations between various immune cells in the prognostic model. Furthermore, we validated the correlation of MAPK1, PIK3CG, and EDIL3 expression and immune cell infiltration in the datasets of GSE162454 from the TISCH database (Fig. 7C). The results showed MAPK1, PIK3CG, and EDIL3 play a valuable role in the fibroblast cell(Fig. 7D). In osteosarcoma, immunotherapy often faces hurdles posed by cancer-associated fibroblasts (CAFs) that secrete dense extracellular matrix components and cytokines. Directly removing CAFs may prove ineffective and even promote tumor metastasis [19]. Therefore, we speculate that the metastasis and deterioration of osteosarcoma can be inhibited by influencing these three targets.

### OncoPredict for drug susceptibility analysis

**Fig. 8:**
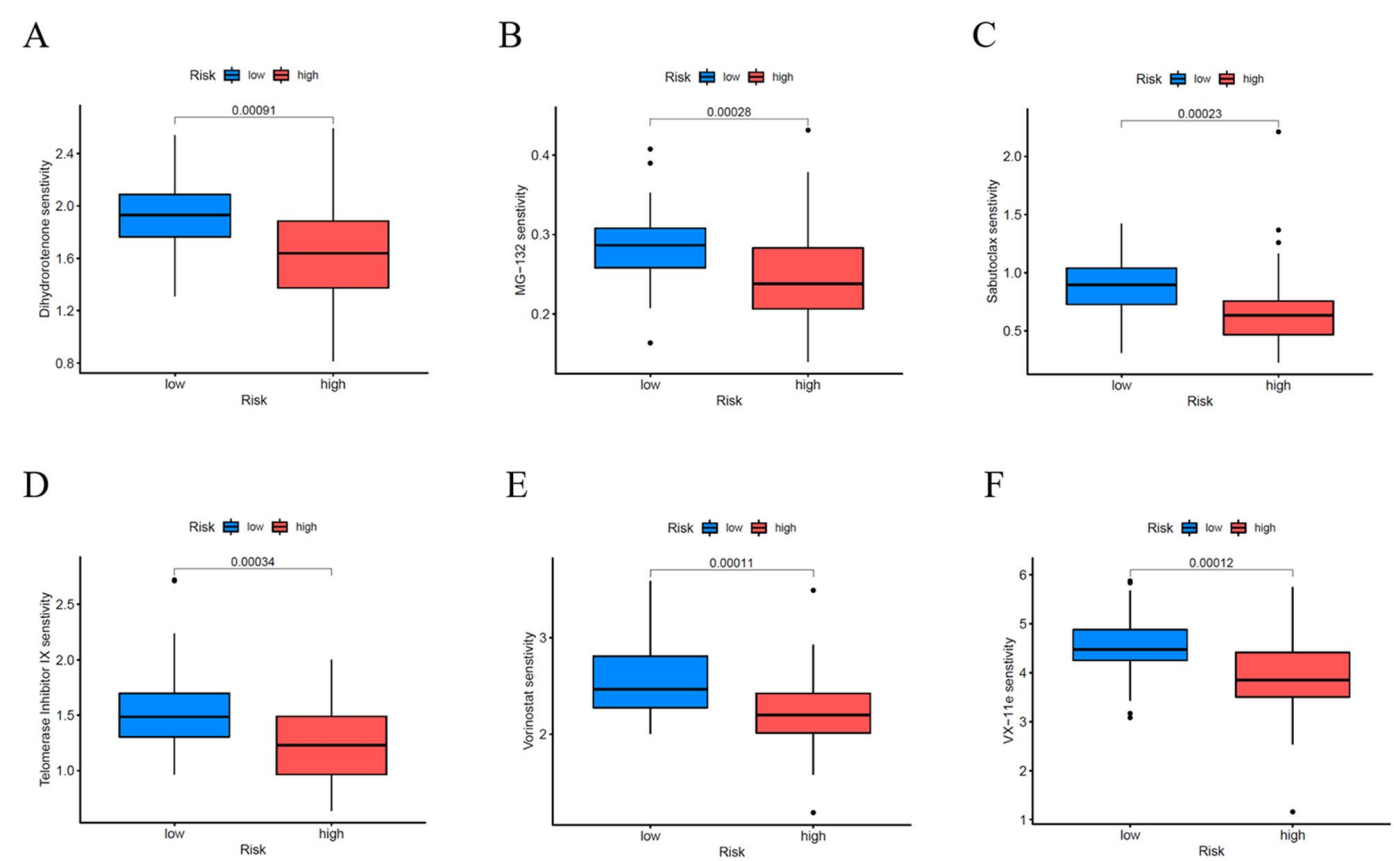
OncoPredict for drug susceptibility analysis

To explore suitable drugs for patients with high-risk scores, we transformed the gene expression of osteosarcoma tissues in the TARGET group into a drug sensitivity matrix using the OncoPredict algorithm (Fig. 8). Osteosarcoma tissues from high-risk group patients were more sensitive to 6 drugs than those osteosarcoma tissues from low-risk group patients, including those of Dihydrorotenone (mitochondrial inhibitor), MG-132 (autophagy activator), Sabutoclax(targeting drug, Bcl-2 inhibitor), Telomerase Inhibitor IX(Telomerase Inhibitor), Vorinostat(HDAC1 inhibitor), and VX-11e ERK inhibitor). These drugs may help in the treatment of osteosarcoma patients with high-risk scores.

## Verification the expression of two predictive genes

**Fig. 9:**
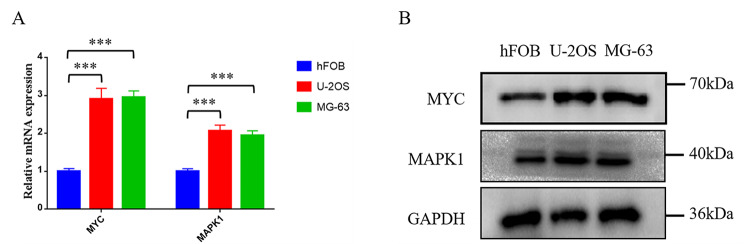
The expression levels of two genes between osteosarcoma cell lines and osteoblasts. (**A**) The qRT-PCR results of MYC and MAPK1. (**B**) Western blotting results of MYC and MAPK1 expression

To verify the expression levels of MAPK1 and MYC in osteosarcoma, we performed Western blotting and RT-PCR analysis on the osteoblast cell line hFOB1.19 and two osteosarcoma cell lines (U-2OS and MG-63). The results showed that the expression levels of MYC and MAPK1 were significantly upregulated in two osteosarcoma cell lines (U-2OS and MG-63) compared with the osteoblast cell line hFOB1.19 (Fig. 9A, B).

### Nilotinib can decrease osteosarcoma cell viability and down-regulate MAPK1 expression

**Fig. 10:**
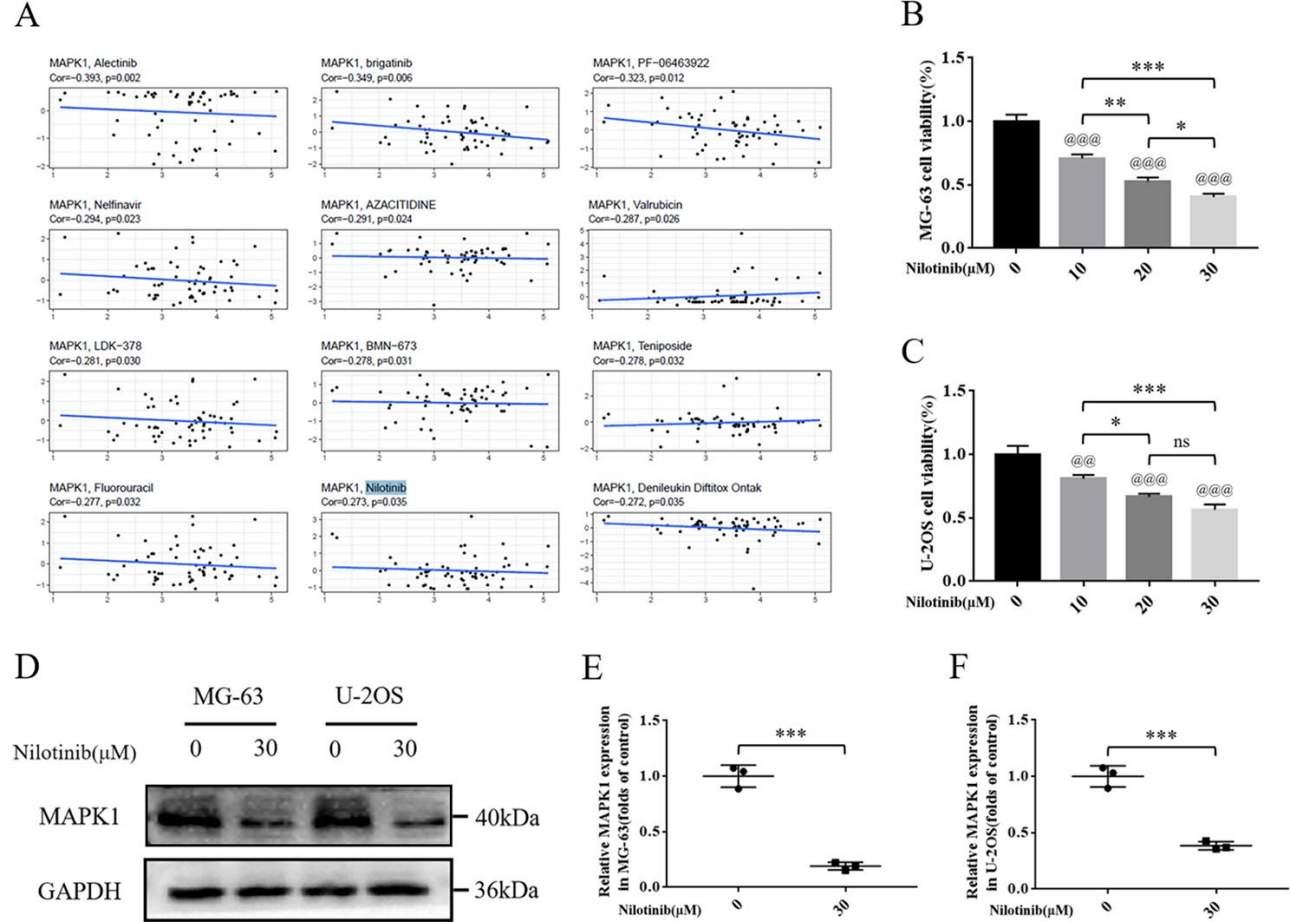
Nilotinib can reduce cell viability and MAPK1 expression in osteosarcoma cells. (**A**) Scatter plot of relationship between MAPK1 expression and drug sensitivity. (**B**) Evaluation of MG-63 osteosarcoma cell viability using CCK-8 assay after exposure to nilotinib for 24 h. (**C**) Evaluation of U-2OS osteosarcoma cell viability using CCK-8 assay after exposure to nilotinib for 24 h. (**D**, **E**, **F**) The expression level of MAPK1 protein in osteosarcoma cells. GAPDH serves as an internal standard. The gels have been run under the same experimental conditions. All experiments were repeated in triplicates (*n* = 3). The obtained data are represented as mean ± SE. Significance: *p-value < 0.05, **p-value < 0.01, ***p-value < 0.001

We further investigated the sensitivity of MAPK1 to chemotherapeutic agents. The results showed that MAPK1 was sensitive to Nilotinib (Fig. 10A, *p* = 0.035). Nilotinib is an anti-vascular targeted agent that promotes apoptosis of several sarcoma cell lines, thereby inhibiting the metastasis of osteosarcoma [20]. However, there are few reports in the literature. To evaluate the therapeutic effect of Nilotinib on osteosarcoma, two types of osteosarcoma cell lines (U-2OS and MG-63) were treated with different doses of Nilotinib (10, 20, and 30 µM). Then the U-2OS and MG-63 cell survival rate was assessed via the CCK-8 kit. We found that Nilotinib has a dose-dependent cytotoxic effect on osteosarcoma cell lines (Fig. 10B, C). Subsequently, we used the western blotting analysis to detect the expression level of MAPK1 in the U-2OS and MG-63 osteosarcoma cell lines in response to Nilotinib (30µM). The results showed that the expression of MAPK1 was down-regulated in both osteosarcoma cell lines after Nilotinib treatment (Fig. 10D, E, F).

### Nilotinib promotes apoptosis in two osteosarcoma cells

**Fig. 11:**
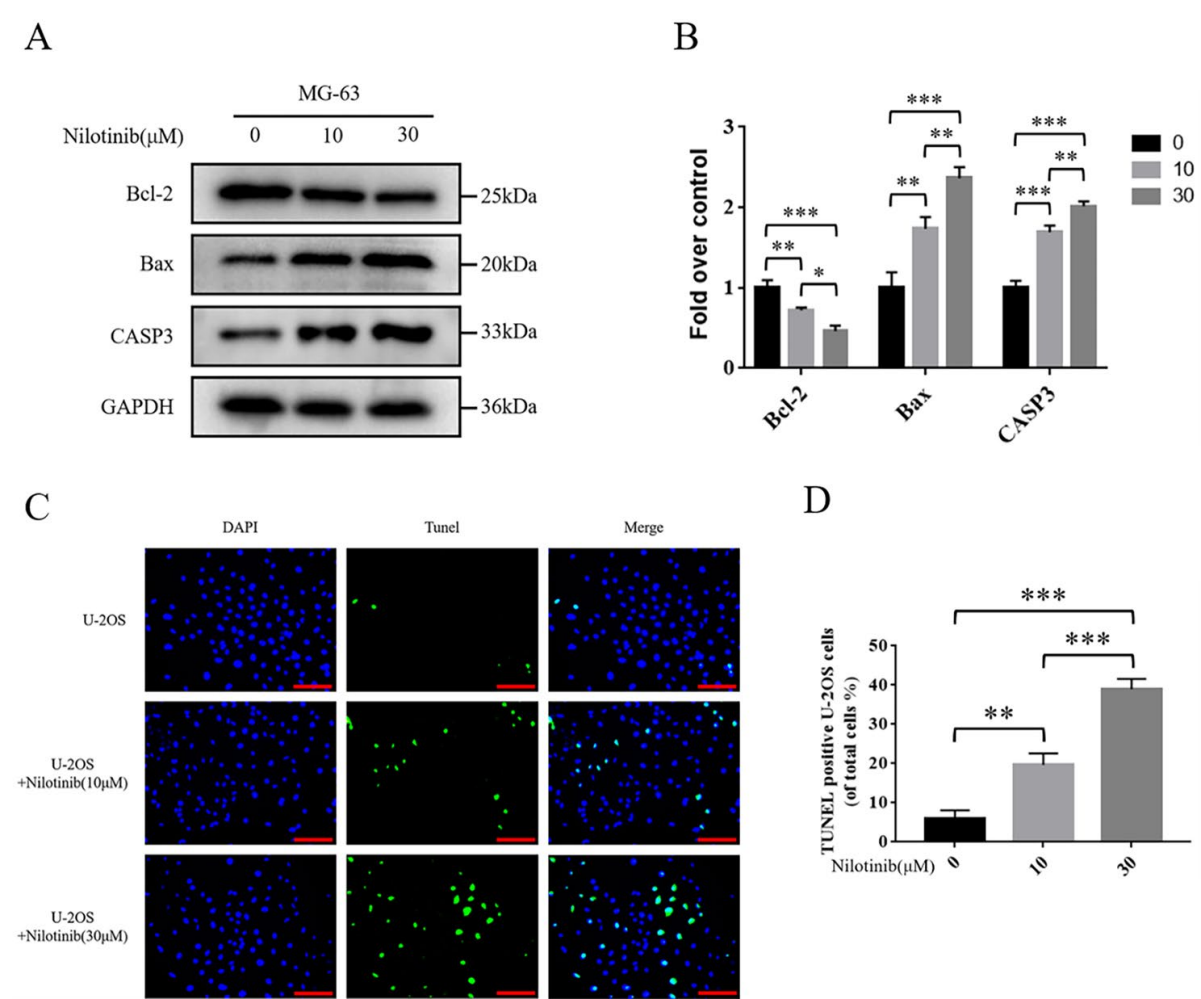
(**A**, **B**) The protein expression levels of Bcl-2, Bax, and CASP3 in osteosarcoma cells. (**C**, **D**) TUNEL staining was used to detect osteosarcoma cell apoptosis (bar: 50 μm; nuclei: blue; positive cells: green). All experiments were repeated in triplicates (*n* = 3). The obtained data are represented as mean ± SE. Significance: *p-value < 0.05, **p-value < 0.01, ***p-value < 0.001

The results of Western blotting analysis demonstrated that the expression levels of Bax and CASP3 were enhanced. In contrast, the expression of Bcl-2 decreased after applying Nilotinib to treat MG-63 osteosarcoma cells. What’s more, the effect of the high-concentration group (30µM) on apoptosis-related proteins in MG-63 osteosarcoma cells was more evident than the low-concentration group (10µM) (Fig. 11A, B). In addition, we applied TUNEL to evaluate the treatment of Nilotinib for U-2OS osteosarcoma cells. As expected, the number of TUNEL-positive cells was upregulated with increased Nilotinib concentration (Fig. 11C, D).

## Discussion

Osteosarcoma, one of the common malignant bone tumors, occurs predominantly in the long bone epiphysis of children and adolescents and often develops metastasis [21]. Effective therapeutic strategies, including surgery, radiotherapy, and chemotherapy, are considered against osteosarcoma [22, 23]. However, the prognosis of osteosarcoma patients is still poor, and the 5-year survival rate is low [24]. Osteosarcoma patients with the same clinical risk factors may significantly differ in prognosis and treatment [25]. Therefore, it is of great significance for the early diagnosis, targeted therapy, and prognosis analysis of osteosarcoma to deeply understand the molecular pathological mechanism and screen key biomarkers related to the occurrence and development of osteosarcoma. At present, many risk scoring systems and prognosis predictions have been developed in clinical application and improvement of patient prognosis management.For example, The four pseudo-genetic markers developed by Xiaoqiang Zhang et al. apply to patients of different sex, age, and metastatic status. These four pseudogenes are involved in the regulation of malignant phenotype, immunity, and DNA/RNA editing, and have a good predictive effect on the treatment of osteosarcoma patients [26]. The result of this study proves that machine learning has a good prediction effect. In addition, the use of various algorithms will take your essay to the next level. Lai et al. used a variety of different algorithms to construct a new regulation network of gene-lncRNA-pathway-immunocyte [27]. miRNA is the main target of function. Therefore we decided to combine the fields of machine learning and biology to develop an entirely new gene predictive model.

Programmed cell death is regulated by various genes and plays an important role in the growth and development of organisms. It is also essential for maintaining tissue and organ homeostasis and is involved in a variety of pathological processes. In addition to apoptosis, iron death, necrotization and cell pyrodeath also contribute to the occurrence and development of cancer [28].Anoikis is an important defense of the organism. Once normal epithelial cells lose contact with the extracellular matrix (ECM), they rapidly undergo apoptosis [29]. However, a common feature of tumor development and growth is the ability of transformed cells to survive under independent growth conditions [30]. This resistance to Anoikis has been shown to be associated with loss of intracellular environmental homeostasis, cancer growth, and metastasis, and this acquired ability is known as lost-nest apoptosis resistance [31]. Cancer cells with Anoikis resistance can spread to distant tissues or organs through the peripheral circulatory system and cause cancer metastasis [32]. In this context, Anoikis resistance is a natural molecular prerequisite for the spread of invasive cancer metastases [33]. Studying the molecular mechanism controlling Anoikis resistance will help search for effective therapies for malignant tumors.

In our study, we comprehensively assessed the Anoikis-related genes in osteosarcoma. We found 28 Anoikis-related genes were differentially expressed between metastasis and non-metastasis groups. Then we used Lasso and SVM-RFE algorithms to screen out feature genes. The two clusters generated by consensus clustering analysis based on the feature genes showed significant differences in survival probability. Next, we constructed a 3-gene risk signature by univariable Cox and multivariate Cox regression analyses. Further, we evaluated the prognostic value of these Anoikis-related gene regulators in training and validation cohorts.

Among the three prognosis gene signatures, MAPK1 was a significant target in osteosarcoma treatment [34]. MAPK1/3 kinase can attenuate Mitophagy and promotes breast cancer bone metastasis [35]. In addition, Mitophagy which plays an important role in carcinogenesis and tumor progression, occurs through an alternative autophagy pathway, requiring the MAPK1 and MAPK14 signaling pathways [36, 37]. Previous studies have shown that ezrin is required for metastasis of osteosarcoma in mouse models, and high expression levels are often associated with adverse outcomes in dogs and patients with osteosarcoma [38]. Ezrin’s ability to attach cell membranes to the actin cytoskeleton allows the cell to interact directly with its microenvironment, thereby facilitating signal transduction through growth factor receptors and adhesion molecules. Furthermore, in mouse models, Ezrin-mediated metastasis survival was found to be partially dependent on MAPK signaling [38, 39]. Therefore, we speculate that MAPK1 may be an effective target to influence the resistance of osteosarcoma patients to anoikis by altering ezrin expression, thereby affecting the prognosis of osteosarcoma patients. MYC is a transcription factor that dimerizes with MAX to bind DNA and regulate gene expression [40]. It has been known that MYC promotes cell growth and proliferation in normal cells, but it also contributes to the genesis of many human cancers [41–43]. MYC could mediate cancer cell energy metabolism and may be a new anticancer therapy [44]. Moreover, previous research also proposes that therapies targeting the MYC pathway will be vital to reversing cancerous growth and restoring antitumor immune responses in patients with MYC-driven cancers [45]. Inhibition of PML could lead to a remarkable growth arrest associated with a decrease in MYC kinase levels [46]. EDIL3 was identified as a novel regulator of epithelial-mesenchymal transition (EMT), contributing to angiogenesis, metastasis, and recurrence of hepatocellular carcinoma [47]. EDIL3 is also a strong and highly accurate diagnostic marker for breast cancer [48]. Moreover, in accordance with previous studies, we found that tumor-derived EDIL3 was involved in tumor-associated bone loss [49]. We believe these three genes may be essential to osteosarcoma’s occurrence, development, and prognosis.

Targeting the tumor immune and bone microenvironment could open up new therapeutic opportunities for patients [50]. According to the immune cell Infiltration results, this new scoring system is closely related to the tumor immune microenvironment. We then used single-cell sequencing analysis to verify our results, showing that the three genes characteristic of the novel scoring model are closely related to immune cells.

Currently, there are many factors that contribute to cancer chemotherapy resistance. For example, the regulation of circular RNA on downstream targets [51, 52]. We want to find new therapeutic targets and drugs. Subsequently, we evaluated the association between relevant prognostic genes and chemotherapeutic drug sensitivity to apply our findings to clinical treatment. From the results, we found that MAPK1 is an important therapeutic target sensitive to various chemotherapy drugs.

We found that the expression level of MAPK1 was significantly decreased in osteosarcoma cells treated with Nilotinib. What’s more, the expression level of MAPK1 decreased in a dose-dependent manner. In addition, through a series of studies, we found that Nilotinib could significantly increase the apoptosis of osteosarcoma cells by down-regulating MAPK1 expression. We suggest that Nilotinib may provide a better therapeutic effect for osteosarcoma patients with elevated MAPK1 expression. MAPK1 may be a key anoikis-related target for the treatment of osteosarcoma.

However, there are some limitations to our study. Firstly, we constructed and validated our risk score model based on public databases, and the sample size was not rich enough. Therefore, our model needs to be further validated based on our clinical data in the future. Moreover, the mechanism of these three predictive genes could be more precise. We will conduct comprehensive functional experiments and multi-omics analysis in our future research.

## Conclusion

In our study, we looked forward to exploring rational prognostic predictors for osteosarcoma patients with metastasis. To predict prognosis, we developed and validated an Anoikis-based risk score system for osteosarcoma patients with metastasis. The AUC value also showed this Anoikis-related risk score system had a good prediction performance. Our study provides a novel gene signature for predicting the prognosis of osteosarcoma patients with metastasis. It opens an avenue for future studies of the relationships between Anoikis-related genes and immunity in these patients. Finally, MAPK1 may be a vital biotherapeutic target.

### Electronic supplementary material

Below is the link to the electronic supplementary material.

Supplementary Material 1

Supplementary Material 2

Supplementary Material 3

## Data Availability

The data that support the findings of this study are openly available in the Therapeutically Applicable Research to Generate Effective Treatments (TARGET; https://ocg.cancer.gov/programs/target) and GEO (https://www.ncbi.nlm.nih.gov/geo/) databases at GSE21257 and GSE162454.
