# Supplementary material for: System analysis based on Anoikis-related genes identifies MAPK1 as a novel therapy target for osteosarcoma with neoadjuvant chemotherapy
Source: BMC Musculoskelet Disord. 2024 Jun 4;25:437. doi: 10.1186/s12891-024-07547-2 (PMC11149263; doi:10.1186/s12891-024-07547-2)
Supplement: Supplementary file 1 — Supplementary Material 1 [file 12891_2024_7547_MOESM1_ESM.pdf]

Anoikis-related gene lists from the Genecards database

| Gene Sym | Descriptio | Category | Gifts | GC Id | Relevance | GeneCards Link |
|---|---|---|---|---|---|---|
| BRMS1 | BRMS1 Tra | Protein Co | 38 | GC11M09 | 14.7102 | https://www.genecards.org/cgi- |
| PTK2 | Protein Ty | Protein Co | 50 | GC08M14 | 7.287515 | https://www.genecards.org/cgi- |
| NTRK2 | Neurotrop | Protein Co | 56 | GC09P084 | 7.26254 | https://www.genecards.org/cgi- |
| BCL2L11 | BCL2 Like | Protein Co | 47 | GC02P111 | 6.691599 | https://www.genecards.org/cgi- |
| SRC | SRC Proto | Protein Co | 54 | GC20P037 | 6.184272 | https://www.genecards.org/cgi- |
| CEACAM6 | CEA Cell A | Protein Co | 44 | GC19P041 | 6.103184 | https://www.genecards.org/cgi- |
| CAV1 | Caveolin 1 | Protein Co | 51 | GC07P116 | 5.463298 | https://www.genecards.org/cgi- |
| AKT1 | AKT Serine | Protein Co | 56 | GC14M10 | 5.40746 | https://www.genecards.org/cgi- |
| ITGB1 | Integrin Su | Protein Co | 53 | GC10M03 | 5.002957 | https://www.genecards.org/cgi- |
| CEACAM5 | CEA Cell A | Protein Co | 45 | GC19P069 | 4.655791 | https://www.genecards.org/cgi- |
| EGFR | Epidermal | Protein Co | 58 | GC07P055 | 4.615613 | https://www.genecards.org/cgi- |
| BCL2 | BCL2 Apo | Protein Co | 53 | GC18M06 | 4.541405 | https://www.genecards.org/cgi- |
| CASP8 | Caspase 8 | Protein Co | 55 | GC02P201 | 4.488767 | https://www.genecards.org/cgi- |
| SIK1 | Salt Induci | Protein Co | 48 | GC21M04 | 4.375074 | https://www.genecards.org/cgi- |
| PTRH2 | Peptidyl-T | Protein Co | 45 | GC17M05 | 4.192896 | https://www.genecards.org/cgi- |
| STAT3 | Signal Tra | Protein Co | 56 | GC17M04 | 4.140685 | https://www.genecards.org/cgi- |
| TLE1 | TLE Family | Protein Co | 47 | GC09M08 | 4.070988 | https://www.genecards.org/cgi- |
| DAPK2 | Death Ass | Protein Co | 43 | GC15M06 | 3.988476 | https://www.genecards.org/cgi- |
| CTNNB1 | Catenin Be | Protein Co | 56 | GC03P041 | 3.976931 | https://www.genecards.org/cgi- |
| ZNF304 | Zinc Finge | Protein Co | 32 | GC19P057 | 3.940119 | https://www.genecards.org/cgi- |
| MAPK1 | Mitogen- | Protein Co | 54 | GC22M02 | 3.734493 | https://www.genecards.org/cgi- |
| BMF | Bcl2 Modi | Protein Co | 40 | GC15M04 | 3.73254 | https://www.genecards.org/cgi- |
| ITGA5 | Integrin Su | Protein Co | 52 | GC12M05 | 3.677547 | https://www.genecards.org/cgi- |
| TP53 | Tumor Pro | Protein Co | 55 | GC17M00 | 3.639736 | https://www.genecards.org/cgi- |
| MCL1 | MCL1 Apo | Protein Co | 50 | GC01M15 | 3.579083 | https://www.genecards.org/cgi- |
| BCL2L1 | BCL2 Like | Protein Co | 50 | GC20M03 | 3.362039 | https://www.genecards.org/cgi- |
| CASP3 | Caspase 3 | Protein Co | 52 | GC04M18 | 3.11994 | https://www.genecards.org/cgi- |
| CDH1 | Cadherin 1 | Protein Co | 52 | GC16P068 | 3.059575 | https://www.genecards.org/cgi- |
| BAD | BCL2 Asso | Protein Co | 47 | GC11M09 | 2.96305 | https://www.genecards.org/cgi- |
| PIK3CA | Phosphati | Protein Co | 55 | GC03P179 | 2.942048 | https://www.genecards.org/cgi- |
| PAK1 | P21 (RAC1 | Protein Co | 52 | GC11M09 | 2.93086 | https://www.genecards.org/cgi- |
| ITGAV | Integrin Su | Protein Co | 51 | GC02P186 | 2.875284 | https://www.genecards.org/cgi- |
| FN1 | Fibronecti | Protein Co | 52 | GC02M21 | 2.821428 | https://www.genecards.org/cgi- |
| MAPK3 | Mitogen- | Protein Co | 51 | GC16M03 | 2.729859 | https://www.genecards.org/cgi- |
| PTGS2 | Prostaglan | Protein Co | 51 | GC01M18 | 2.692126 | https://www.genecards.org/cgi- |
| BAX | BCL2 Asso | Protein Co | 52 | GC19P048 | 2.548128 | https://www.genecards.org/cgi- |
| BCAR1 | BCAR1 Sca | Protein Co | 46 | GC16M07 | 2.548128 | https://www.genecards.org/cgi- |
| PTEN | Phosphata | Protein Co | 54 | GC10P093 | 2.518463 | https://www.genecards.org/cgi- |
| ERBB2 | Erb-B2 Re | Protein Co | 58 | GC17P039 | 2.433978 | https://www.genecards.org/cgi- |
| ANGPTL4 | Angiopoie | Protein Co | 47 | GC19P008 | 2.417242 | https://www.genecards.org/cgi- |
| PDK4 | Pyruvate D | Protein Co | 46 | GC07M09 | 2.415361 | https://www.genecards.org/cgi- |
| CYCS | Cytochron | Protein Co | 51 | GC07M02 | 2.339439 | https://www.genecards.org/cgi- |
| BRAF | B-Raf Prot | Protein Co | 55 | GC07M14 | 2.335299 | https://www.genecards.org/cgi- |
| YAP1 | Yes1 Asso | Protein Co | 50 | GC11P102 | 2.33217 | https://www.genecards.org/cgi- |
| ANKRD13 | Ankyrin Re | Protein Co | 34 | GC01M07 | 2.329537 | https://www.genecards.org/cgi- |
| ITGA2 | Integrin Su | Protein Co | 49 | GC05P052 | 2.298167 | https://www.genecards.org/cgi- |
| ANXA5 | Annexin A | Protein Co | 48 | GC04M12 | 2.265912 | https://www.genecards.org/cgi- |
| BIRC5 | Baculovira | Protein Co | 48 | GC17P078 | 2.255684 | https://www.genecards.org/cgi- |
| MTOR | Mechanist | Protein Co | 56 | GC01M01 | 2.249703 | https://www.genecards.org/cgi- |
| TIMP1 | TIMP Meta | Protein Co | 47 | GC0XP047 | 2.24534 | https://www.genecards.org/cgi- |
| BDNF | Brain Deri | Protein Co | 52 | GC11M02 | 2.221462 | https://www.genecards.org/cgi- |
| CSPG4 | Chondroiti | Protein Co | 50 | GC15M07 | 2.195114 | https://www.genecards.org/cgi- |
| BSG | Basigin (O | Protein Co | 47 | GC19P000 | 2.195114 | https://www.genecards.org/cgi- |
| AKT2 | AKT Serine | Protein Co | 56 | GC19M04 | 2.182597 | https://www.genecards.org/cgi- |
| STK11 | Serine/Thr | Protein Co | 53 | GC19P001 | 2.150353 | https://www.genecards.org/cgi- |

| | | | | | | |
|---|---|---|---|---|---|---|
| IGF1 | Insulin Like | Protein Co | 50 | GC12M102 | 2.147692 | https://www.genecards.org/cgi- |
| IGF1R | Insulin Like | Protein Co | 57 | GC15P098 | 2.146957 | https://www.genecards.org/cgi- |
| ITGA6 | Integrin Su | Protein Co | 52 | GC02P172 | 2.10977 | https://www.genecards.org/cgi- |
| ILK | Integrin Lin | Protein Co | 48 | GC11P006 | 2.083155 | https://www.genecards.org/cgi- |
| CFLAR | CASP8 And | Protein Co | 46 | GC02P201 | 2.083022 | https://www.genecards.org/cgi- |
| RHOA | Ras Homo | Protein Co | 52 | GC03M049 | 2.068578 | https://www.genecards.org/cgi- |
| HIF1A | Hypoxia In | Protein Co | 51 | GC14P061 | 2.064971 | https://www.genecards.org/cgi- |
| DAP3 | Death Asso | Protein Co | 42 | GC01P155 | 2.0553 | https://www.genecards.org/cgi- |
| MYBBP1A | MYB Bindi | Protein Co | 41 | GC17M004 | 2.020877 | https://www.genecards.org/cgi- |
| TLE5 | TLE Family | Protein Co | 39 | GC19M006 | 1.998172 | https://www.genecards.org/cgi- |
| ITGA3 | Integrin Su | Protein Co | 51 | GC17P050 | 1.998085 | https://www.genecards.org/cgi- |
| PTK2B | Protein Ty | Protein Co | 51 | GC08P027 | 1.99505 | https://www.genecards.org/cgi- |
| CCND1 | Cyclin D1 | Protein Co | 55 | GC11P069 | 1.981372 | https://www.genecards.org/cgi- |
| CTTN | Cortactin | Protein Co | 45 | GC11P070 | 1.981372 | https://www.genecards.org/cgi- |
| CALR | Calreticulin | Protein Co | 54 | GC19P012 | 1.944715 | https://www.genecards.org/cgi- |
| ATF4 | Activating | Protein Co | 47 | GC22P039 | 1.944715 | https://www.genecards.org/cgi- |
| CDCP1 | CUB Doma | Protein Co | 41 | GC03M045 | 1.931936 | https://www.genecards.org/cgi- |
| PLAUR | Plasminog | Protein Co | 47 | GC19M043 | 1.907844 | https://www.genecards.org/cgi- |
| SKP2 | S-Phase K | Protein Co | 46 | GC05P036 | 1.907844 | https://www.genecards.org/cgi- |
| CHEK2 | Checkpoin | Protein Co | 56 | GC22M028 | 1.907213 | https://www.genecards.org/cgi- |
| HGF | Hepatocyt | Protein Co | 54 | GC07M081 | 1.883366 | https://www.genecards.org/cgi- |
| E2F1 | E2F Transc | Protein Co | 45 | GC20M033 | 1.879978 | https://www.genecards.org/cgi- |
| EGF | Epidermal | Protein Co | 54 | GC04P109 | 1.870717 | https://www.genecards.org/cgi- |
| PIK3CG | Phosphatid | Protein Co | 50 | GC07P106 | 1.86744 | https://www.genecards.org/cgi- |
| ITGB4 | Integrin Su | Protein Co | 52 | GC17P075 | 1.855637 | https://www.genecards.org/cgi- |
| DAPK1 | Death Asso | Protein Co | 52 | GC09P087 | 1.845545 | https://www.genecards.org/cgi- |
| MAPK8 | Mitogen-A | Protein Co | 52 | GC10P048 | 1.839446 | https://www.genecards.org/cgi- |
| PIK3R1 | Phosphoin | Protein Co | 53 | GC05P068 | 1.820627 | https://www.genecards.org/cgi- |
| PIK3R3 | Phosphoin | Protein Co | 45 | GC01M046 | 1.81566 | https://www.genecards.org/cgi- |
| MAP2K1 | Mitogen-A | Protein Co | 55 | GC15P066 | 1.79616 | https://www.genecards.org/cgi- |
| CXCL12 | C-X-C Mo | Protein Co | 46 | GC10M044 | 1.77687 | https://www.genecards.org/cgi- |
| LGALS3 | Galectin 3 | Protein Co | 48 | GC14P055 | 1.741451 | https://www.genecards.org/cgi- |
| FBXW7-AS | FBXW7 An | RNA Gene | 15 | GC04P152 | 1.73386 | https://www.genecards.org/cgi- |
| BAK1 | BCL2 Anta | Protein Co | 47 | GC06M033 | 1.730096 | https://www.genecards.org/cgi- |
| ABHD4 | Abhydrola | Protein Co | 41 | GC14P033 | 1.706411 | https://www.genecards.org/cgi- |
| CD44 | CD44 Mole | Protein Co | 50 | GC11P035 | 1.702935 | https://www.genecards.org/cgi- |
| ITGA4 | Integrin Su | Protein Co | 50 | GC02P181 | 1.689653 | https://www.genecards.org/cgi- |
| FADD | Fas Associ | Protein Co | 48 | GC11P070 | 1.689653 | https://www.genecards.org/cgi- |
| PHLDA2 | Pleckstrin | Protein Co | 39 | GC11M002 | 1.689653 | https://www.genecards.org/cgi- |
| TGFB1 | Transform | Protein Co | 55 | GC19M041 | 1.685504 | https://www.genecards.org/cgi- |
| HMCN1 | Hemicentin | Protein Co | 41 | GC01P185 | 1.685504 | https://www.genecards.org/cgi- |
| MMP2 | Matrix Me | Protein Co | 55 | GC16P055 | 1.672284 | https://www.genecards.org/cgi- |
| CEBPB | CCAAT En | Protein Co | 45 | GC20P050 | 1.672284 | https://www.genecards.org/cgi- |
| CEMIP | Cell Migra | Protein Co | 39 | GC15P080 | 1.672284 | https://www.genecards.org/cgi- |
| CDKN3 | Cyclin Dep | Protein Co | 44 | GC14P054 | 1.666558 | https://www.genecards.org/cgi- |
| CBL | Cbl Proto- | Protein Co | 54 | GC11P119 | 1.654234 | https://www.genecards.org/cgi- |
| CASP9 | Caspase 9 | Protein Co | 49 | GC01M015 | 1.654234 | https://www.genecards.org/cgi- |
| SFN | Stratifin | Protein Co | 48 | GC01P028 | 1.654234 | https://www.genecards.org/cgi- |
| MTDH | Metadheri | Protein Co | 43 | GC08P097 | 1.654234 | https://www.genecards.org/cgi- |
| PRKCA | Protein Kir | Protein Co | 53 | GC17P066 | 1.635414 | https://www.genecards.org/cgi- |
| TNFRSF10 | TNF Recep | Protein Co | 52 | GC08M023 | 1.635414 | https://www.genecards.org/cgi- |
| CXCL8 | C-X-C Mo | Protein Co | 44 | GC04P073 | 1.635414 | https://www.genecards.org/cgi- |
| MIR200C | MicroRNA | RNA Gene | 26 | GC12P022 | 1.635414 | https://www.genecards.org/cgi- |
| AR | Androgen | Protein Co | 54 | GC0XP067 | 1.618418 | https://www.genecards.org/cgi- |
| CDKN2A | Cyclin Dep | Protein Co | 52 | GC09M021 | 1.615718 | https://www.genecards.org/cgi- |
| CPT1A | Carnitine P | Protein Co | 51 | GC11M068 | 1.615718 | https://www.genecards.org/cgi- |
| PIK3CB | Phosphatid | Protein Co | 50 | GC03M138 | 1.615718 | https://www.genecards.org/cgi- |
| CLDN1 | Claudin 1 | Protein Co | 48 | GC03M190 | 1.615718 | https://www.genecards.org/cgi- |

| | | | | | | |
|---|---|---|---|---|---|---|
| MIR204 | MicroRNA | RNA Gene | 25 | GC09M07( | 1.615718 | https://www.genecards.org/cgi-I |
| MIR26A1 | MicroRNA | RNA Gene | 23 | GC03P037 | 1.615718 | https://www.genecards.org/cgi-I |
| CDKN1A | Cyclin Dep | Protein Co | 51 | GC06P087 | 1.595009 | https://www.genecards.org/cgi-I |
| CDKN1B | Cyclin Dep | Protein Co | 50 | GC12P022 | 1.595009 | https://www.genecards.org/cgi-I |
| KLF12 | KLF Transc | Protein Co | 40 | GC13M073 | 1.595009 | https://www.genecards.org/cgi-I |
| NTRK1 | Neurotrop | Protein Co | 55 | GC01P156 | 1.573605 | https://www.genecards.org/cgi-I |
| PLAU | Plasminog | Protein Co | 55 | GC10P073 | 1.573115 | https://www.genecards.org/cgi-I |
| MYC | MYC Proto | Protein Co | 54 | GC08P127 | 1.573115 | https://www.genecards.org/cgi-I |
| SMAD4 | SMAD Fan | Protein Co | 54 | GC18P051 | 1.573115 | https://www.genecards.org/cgi-I |
| PLK1 | Polo Like I | Protein Co | 53 | GC16P024 | 1.573115 | https://www.genecards.org/cgi-I |
| MUC1 | Mucin 1, C | Protein Co | 51 | GC01M15! | 1.573115 | https://www.genecards.org/cgi-I |
| LGALS1 | Galectin 1 | Protein Co | 45 | GC22P037 | 1.573115 | https://www.genecards.org/cgi-I |
| PYCARD | PYD And ( | Protein Co | 44 | GC16M031 | 1.573115 | https://www.genecards.org/cgi-I |
| SESN2 | Sestrin 2 | Protein Co | 40 | GC01P028 | 1.573115 | https://www.genecards.org/cgi-I |
| ITGB3 | Integrin Su | Protein Co | 55 | GC17P058 | 1.568464 | https://www.genecards.org/cgi-I |
| KRAS | KRAS Prot | Protein Co | 54 | GC12M02! | 1.568464 | https://www.genecards.org/cgi-I |
| THBS1 | Thrombos | Protein Co | 48 | GC15P039 | 1.549803 | https://www.genecards.org/cgi-I |
| BID | BH3 Intera | Protein Co | 47 | GC22M017 | 1.549803 | https://www.genecards.org/cgi-I |
| HRAS | HRas Prot | Protein Co | 55 | GC11M003 | 1.535089 | https://www.genecards.org/cgi-I |
| CDK11B | Cyclin Dep | Protein Co | 43 | GC01M007 | 1.524758 | https://www.genecards.org/cgi-I |
| CDK11A | Cyclin Dep | Protein Co | 41 | GC01M001 | 1.524758 | https://www.genecards.org/cgi-I |
| XIAP | X-Linked I | Protein Co | 54 | GC0XP123 | 1.518128 | https://www.genecards.org/cgi-I |
| PPARG | Peroxisom | Protein Co | 55 | GC03P012 | 1.497523 | https://www.genecards.org/cgi-I |
| IL6 | Interleukin | Protein Co | 53 | GC07P022 | 1.497523 | https://www.genecards.org/cgi-I |
| MIR145 | MicroRNA | RNA Gene | 25 | GC05P149 | 1.497523 | https://www.genecards.org/cgi-I |
| CCR7 | C-C Motif | Protein Co | 47 | GC17M04! | 1.467398 | https://www.genecards.org/cgi-I |
| MSLN | Mesothelir | Protein Co | 45 | GC16P012 | 1.467398 | https://www.genecards.org/cgi-I |
| RAC1 | Rac Family | Protein Co | 51 | GC07P006 | 1.461561 | https://www.genecards.org/cgi-I |
| GRHL2 | Grainyhea | Protein Co | 43 | GC08P101 | 1.461561 | https://www.genecards.org/cgi-I |
| BIRC3 | Baculovira | Protein Co | 48 | GC11P102 | 1.448279 | https://www.genecards.org/cgi-I |
| NOTCH1 | Notch Rec | Protein Co | 55 | GC09M137 | 1.438149 | https://www.genecards.org/cgi-I |
| RHOG | Ras Homo | Protein Co | 43 | GC11M004 | 1.436139 | https://www.genecards.org/cgi-I |
| CCAR2 | Cell Cycle | Protein Co | 39 | GC08P022 | 1.436139 | https://www.genecards.org/cgi-I |
| NQO1 | NAD(P)H ( | Protein Co | 50 | GC16M06! | 1.433205 | https://www.genecards.org/cgi-I |
| MMP13 | Matrix Me | Protein Co | 54 | GC11M102 | 1.398545 | https://www.genecards.org/cgi-I |
| FAS | Fas Cell Su | Protein Co | 53 | GC10P093 | 1.395647 | https://www.genecards.org/cgi-I |
| MTA1 | Metastasis | Protein Co | 46 | GC14P105 | 1.395647 | https://www.genecards.org/cgi-I |
| MYO5A | Myosin VA | Protein Co | 47 | GC15M12( | 1.392646 | https://www.genecards.org/cgi-I |
| EDA2R | Ectodyspla | Protein Co | 45 | GC0XM06( | 1.392646 | https://www.genecards.org/cgi-I |
| CCN6 | Cellular Cc | Protein Co | 43 | GC06P112 | 1.392646 | https://www.genecards.org/cgi-I |
| MMP9 | Matrix Me | Protein Co | 56 | GC20P046 | 1.374343 | https://www.genecards.org/cgi-I |
| ABL1 | ABL Proto | Protein Co | 55 | GC09P130 | 1.374343 | https://www.genecards.org/cgi-I |
| MAPK11 | Mitogen-/ | Protein Co | 51 | GC22M05( | 1.374343 | https://www.genecards.org/cgi-I |
| SOD2 | Superoxid | Protein Co | 51 | GC06M15! | 1.374343 | https://www.genecards.org/cgi-I |
| PTHLH | Parathyroi | Protein Co | 48 | GC12M027 | 1.370602 | https://www.genecards.org/cgi-I |
| PDGFB | Platelet De | Protein Co | 53 | GC22M06( | 1.353635 | https://www.genecards.org/cgi-I |
| GLI2 | GLI Family | Protein Co | 51 | GC02P120 | 1.353635 | https://www.genecards.org/cgi-I |
| EZH2 | Enhancer ( | Protein Co | 56 | GC07M148 | 1.352577 | https://www.genecards.org/cgi-I |
| RIPK1 | Receptor I | Protein Co | 51 | GC06P003 | 1.351373 | https://www.genecards.org/cgi-I |
| CXCR4 | C-X-C Mo | Protein Co | 55 | GC02M13( | 1.343367 | https://www.genecards.org/cgi-I |
| HMGA1 | High Mobi | Protein Co | 48 | GC06P087 | 1.331741 | https://www.genecards.org/cgi-I |
| SIK2 | Salt Induci | Protein Co | 48 | GC11P111 | 1.331741 | https://www.genecards.org/cgi-I |
| TNFSF10 | TNF Super | Protein Co | 47 | GC03M172 | 1.331741 | https://www.genecards.org/cgi-I |
| ANGPTL2 | Angiopoie | Protein Co | 40 | GC09M127 | 1.313242 | https://www.genecards.org/cgi-I |
| S100A4 | S100 Calci | Protein Co | 48 | GC01M153 | 1.308429 | https://www.genecards.org/cgi-I |
| NTF3 | Neurotrop | Protein Co | 47 | GC12P022 | 1.308429 | https://www.genecards.org/cgi-I |
| ETV4 | ETS Varian | Protein Co | 45 | GC17M043 | 1.308429 | https://www.genecards.org/cgi-I |
| MIR21 | MicroRNA | RNA Gene | 26 | GC17P059 | 1.308429 | https://www.genecards.org/cgi-I |

| | | | | | | |
|---|---|---|---|---|---|---|
| MIR124-1 | MicroRNA | RNA Gene | 21 | GC08M00 | 1.308429 | https://www.genecards.org/cgi- |
| HTRA1 | HtrA Serin | Protein Co | 48 | GC10P122 | 1.283384 | https://www.genecards.org/cgi- |
| LATS1 | Large Tum | Protein Co | 46 | GC06M14 | 1.283384 | https://www.genecards.org/cgi- |
| CEACAM3 | CEA Cell A | Protein Co | 45 | GC19P041 | 1.283384 | https://www.genecards.org/cgi- |
| EIF2AK3 | Eukaryotic | Protein Co | 51 | GC02M08 | 1.280375 | https://www.genecards.org/cgi- |
| LAMC2 | Laminin Su | Protein Co | 50 | GC01P183 | 1.280375 | https://www.genecards.org/cgi- |
| LAMA3 | Laminin Su | Protein Co | 48 | GC18P023 | 1.280375 | https://www.genecards.org/cgi- |
| LAMB3 | Laminin Su | Protein Co | 48 | GC01M20 | 1.280375 | https://www.genecards.org/cgi- |
| CDH2 | Cadherin 2 | Protein Co | 55 | GC18M02 | 1.260679 | https://www.genecards.org/cgi- |
| CSNK2A1 | Casein Kin | Protein Co | 53 | GC20M00 | 1.260679 | https://www.genecards.org/cgi- |
| EDIL3 | EGF Like R | Protein Co | 43 | GC05M08 | 1.260679 | https://www.genecards.org/cgi- |
| ZEB2 | Zinc Finge | Protein Co | 50 | GC02M14 | 1.256149 | https://www.genecards.org/cgi- |
| TLN1 | Talin 1 | Protein Co | 44 | GC09M03 | 1.256149 | https://www.genecards.org/cgi- |
| EPHA2 | EPH Recep | Protein Co | 56 | GC01M01 | 1.239971 | https://www.genecards.org/cgi- |
| SIRT3 | Sirtuin 3 | Protein Co | 50 | GC11M00 | 1.239971 | https://www.genecards.org/cgi- |
| OLFM3 | Olfactome | Protein Co | 40 | GC01M10 | 1.239971 | https://www.genecards.org/cgi- |
| CLU | Clusterin | Protein Co | 50 | GC08M02 | 1.226024 | https://www.genecards.org/cgi- |
| SPINK1 | Serine Pep | Protein Co | 44 | GC05M14 | 1.226024 | https://www.genecards.org/cgi- |
| CPEB2 | Cytoplasm | Protein Co | 36 | GC04P018 | 1.226024 | https://www.genecards.org/cgi- |
| NAT1 | N-Acetyltr | Protein Co | 45 | GC08P018 | 1.218076 | https://www.genecards.org/cgi- |
| TSG101 | Tumor Sus | Protein Co | 45 | GC11M01 | 1.218076 | https://www.genecards.org/cgi- |
| MIR200A | MicroRNA | RNA Gene | 22 | GC01P004 | 1.218076 | https://www.genecards.org/cgi- |
| MIR6744 | MicroRNA | RNA Gene | 9 | GC11P001 | 1.218076 | https://www.genecards.org/cgi- |
| SERPINA1 | Serpin Fan | Protein Co | 51 | GC14M09 | 1.212177 | https://www.genecards.org/cgi- |
| AKT3 | AKT Serine | Protein Co | 57 | GC01M24 | 1.194765 | https://www.genecards.org/cgi- |
| RELA | RELA Prot | Protein Co | 54 | GC11M06 | 1.194765 | https://www.genecards.org/cgi- |
| TNFRSF1A | TNF Recep | Protein Co | 52 | GC12M00 | 1.194765 | https://www.genecards.org/cgi- |
| FASLG | Fas Ligand | Protein Co | 50 | GC01P172 | 1.194765 | https://www.genecards.org/cgi- |
| AFP | Alpha Fetc | Protein Co | 48 | GC04P073 | 1.194765 | https://www.genecards.org/cgi- |
| ITGA8 | Integrin Su | Protein Co | 48 | GC10M01 | 1.194765 | https://www.genecards.org/cgi- |
| NOX4 | NADPH O | Protein Co | 47 | GC11M08 | 1.194765 | https://www.genecards.org/cgi- |
| PBK | PDZ Bindir | Protein Co | 47 | GC08M02 | 1.194765 | https://www.genecards.org/cgi- |
| SATB1 | SATB Hom | Protein Co | 47 | GC03M02 | 1.194765 | https://www.genecards.org/cgi- |
| CD63 | CD63 Mol | Protein Co | 45 | GC12M05 | 1.194765 | https://www.genecards.org/cgi- |
| EEF1A1 | Eukaryotic | Protein Co | 45 | GC06M07 | 1.194765 | https://www.genecards.org/cgi- |
| LTB4R2 | Leukotrien | Protein Co | 45 | GC14P033 | 1.194765 | https://www.genecards.org/cgi- |
| MAVS | Mitochonc | Protein Co | 41 | GC20P004 | 1.194765 | https://www.genecards.org/cgi- |
| HRC | Histidine R | Protein Co | 39 | GC19M04 | 1.194765 | https://www.genecards.org/cgi- |
| CCN2 | Cellular Cc | Protein Co | 48 | GC06M13 | 1.191831 | https://www.genecards.org/cgi- |
| RHOB | Ras Homo | Protein Co | 47 | GC02P020 | 1.191831 | https://www.genecards.org/cgi- |
| PPP1R13B | Protein Ph | Protein Co | 40 | GC14M10 | 1.191831 | https://www.genecards.org/cgi- |
| PLG | Plasminog | Protein Co | 52 | GC06P160 | 1.185631 | https://www.genecards.org/cgi- |
| MET | MET Proto | Protein Co | 56 | GC07P116 | 1.184292 | https://www.genecards.org/cgi- |
| RAF1 | Raf-1 Prot | Protein Co | 57 | GC03M01 | 1.16972 | https://www.genecards.org/cgi- |
| PARP1 | Poly(ADP- | Protein Co | 53 | GC01M22 | 1.16972 | https://www.genecards.org/cgi- |
| PRKCQ | Protein Kir | Protein Co | 51 | GC10M00 | 1.16972 | https://www.genecards.org/cgi- |
| BRCA2 | BRCA2 DN | Protein Co | 50 | GC13P032 | 1.16972 | https://www.genecards.org/cgi- |
| RB1 | RB Transcr | Protein Co | 50 | GC13P048 | 1.16972 | https://www.genecards.org/cgi- |
| SP1 | Sp1 Transc | Protein Co | 49 | GC12P053 | 1.16972 | https://www.genecards.org/cgi- |
| HAVCR2 | Hepatitis A | Protein Co | 48 | GC05M15 | 1.16972 | https://www.genecards.org/cgi- |
| DOCK1 | Dedicator | Protein Co | 47 | GC10P126 | 1.16972 | https://www.genecards.org/cgi- |
| VTN | Vitronectir | Protein Co | 47 | GC17M04 | 1.16972 | https://www.genecards.org/cgi- |
| INHBB | Inhibin Sul | Protein Co | 45 | GC02P123 | 1.16972 | https://www.genecards.org/cgi- |
| PDCD4 | Programm | Protein Co | 45 | GC10P110 | 1.16972 | https://www.genecards.org/cgi- |
| PRPF4B | Pre-MRNA | Protein Co | 44 | GC06P004 | 1.16972 | https://www.genecards.org/cgi- |
| RANBP9 | RAN Bindi | Protein Co | 44 | GC06M01 | 1.16972 | https://www.genecards.org/cgi- |
| SESN1 | Sestrin 1 | Protein Co | 43 | GC06M10 | 1.16972 | https://www.genecards.org/cgi- |
| SESN3 | Sestrin 3 | Protein Co | 41 | GC11M09 | 1.16972 | https://www.genecards.org/cgi- |

| | | | | | | |
|---|---|---|---|---|---|---|
| CD24 | CD24 Mol | Protein Co | 40 | GC06M10 | 1.16972 | https://www.genecards.org/cgi- |
| ZBTB7A | Zinc Finge | Protein Co | 40 | GC19M00 | 1.16972 | https://www.genecards.org/cgi- |
| MIR141 | MicroRNA | RNA Gene | 23 | GC12P022 | 1.16972 | https://www.genecards.org/cgi- |
| ELANE | Elastase, N | Protein Co | 53 | GC19P003 | 1.151272 | https://www.genecards.org/cgi- |
| KDR | Kinase Ins | Protein Co | 56 | GC04M05 | 1.142485 | https://www.genecards.org/cgi- |
| MDM2 | MDM2 Pro | Protein Co | 55 | GC12P068 | 1.142485 | https://www.genecards.org/cgi- |
| NFE2L2 | NFE2 Like | Protein Co | 53 | GC02M17 | 1.142485 | https://www.genecards.org/cgi- |
| ZEB1 | Zinc Finge | Protein Co | 51 | GC10P031 | 1.142485 | https://www.genecards.org/cgi- |
| KL | Klotho | Protein Co | 50 | GC13P033 | 1.142485 | https://www.genecards.org/cgi- |
| PRKCI | Protein Kir | Protein Co | 50 | GC03P170 | 1.142485 | https://www.genecards.org/cgi- |
| CRYAB | Crystallin | Protein Co | 48 | GC11M11 | 1.142485 | https://www.genecards.org/cgi- |
| FGF2 | Fibroblast | Protein Co | 48 | GC04P122 | 1.142485 | https://www.genecards.org/cgi- |
| HK2 | Hexokinas | Protein Co | 48 | GC02P074 | 1.142485 | https://www.genecards.org/cgi- |
| LTF | Lactotrans | Protein Co | 48 | GC03M04 | 1.142485 | https://www.genecards.org/cgi- |
| IQGAP1 | IQ Motif C | Protein Co | 46 | GC15P090 | 1.142485 | https://www.genecards.org/cgi- |
| MGAT5 | Alpha-1,6- | Protein Co | 44 | GC02P134 | 1.142485 | https://www.genecards.org/cgi- |
| SDCBP | Syndecan | Protein Co | 44 | GC08P058 | 1.142485 | https://www.genecards.org/cgi- |
| ABHD2 | Abhydrola | Protein Co | 42 | GC15P089 | 1.142485 | https://www.genecards.org/cgi- |
| SPIB | Spi-B Tran | Protein Co | 41 | GC19P050 | 1.142485 | https://www.genecards.org/cgi- |
| TRIM31 | Tripartite | Protein Co | 40 | GC06M06 | 1.142485 | https://www.genecards.org/cgi- |
| MIR1827 | MicroRNA | RNA Gene | 13 | GC12P100 | 1.142485 | https://www.genecards.org/cgi- |
| PDGFRB | Platelet De | Protein Co | 56 | GC05M15 | 1.11236 | https://www.genecards.org/cgi- |
| PLAT | Plasminog | Protein Co | 54 | GC08M04 | 1.11236 | https://www.genecards.org/cgi- |
| TLR3 | Toll Like R | Protein Co | 54 | GC04P186 | 1.11236 | https://www.genecards.org/cgi- |
| NRAS | NRAS Prot | Protein Co | 53 | GC01M11 | 1.11236 | https://www.genecards.org/cgi- |
| ROCK1 | Rho Assoc | Protein Co | 52 | GC18M02 | 1.11236 | https://www.genecards.org/cgi- |
| PAK4 | P21 (RAC1 | Protein Co | 51 | GC19P039 | 1.11236 | https://www.genecards.org/cgi- |
| VEGFA | Vascular E | Protein Co | 51 | GC06P043 | 1.11236 | https://www.genecards.org/cgi- |
| CASP10 | Caspase 1 | Protein Co | 50 | GC02P201 | 1.11236 | https://www.genecards.org/cgi- |
| PIN1 | Peptidylpr | Protein Co | 50 | GC19P009 | 1.11236 | https://www.genecards.org/cgi- |
| IL1RAP | Interleukin | Protein Co | 48 | GC03P190 | 1.11236 | https://www.genecards.org/cgi- |
| UBE2C | Ubiquitin ( | Protein Co | 48 | GC20P045 | 1.11236 | https://www.genecards.org/cgi- |
| YWHAZ | Tyrosine 3 | Protein Co | 48 | GC08M10 | 1.11236 | https://www.genecards.org/cgi- |
| TWIST1 | Twist Fami | Protein Co | 47 | GC07M01 | 1.11236 | https://www.genecards.org/cgi- |
| BMP6 | Bone Mor | Protein Co | 46 | GC06P007 | 1.11236 | https://www.genecards.org/cgi- |
| BNIP3L | BCL2 Inter | Protein Co | 45 | GC08P026 | 1.11236 | https://www.genecards.org/cgi- |
| ELK1 | ETS Transc | Protein Co | 45 | GC0XM04 | 1.11236 | https://www.genecards.org/cgi- |
| KDM3A | Lysine Der | Protein Co | 44 | GC02P086 | 1.11236 | https://www.genecards.org/cgi- |
| PRDX4 | Peroxiredc | Protein Co | 44 | GC0XP023 | 1.11236 | https://www.genecards.org/cgi- |
| BNIP3 | BCL2 Inter | Protein Co | 43 | GC10M13 | 1.11236 | https://www.genecards.org/cgi- |
| LMO3 | LIM Doma | Protein Co | 41 | GC12M01 | 1.11236 | https://www.genecards.org/cgi- |
| ZNF32 | Zinc Finge | Protein Co | 36 | GC10M04 | 1.11236 | https://www.genecards.org/cgi- |
| MIR200B | MicroRNA | RNA Gene | 23 | GC01P001 | 1.11236 | https://www.genecards.org/cgi- |
| MIR525 | MicroRNA | RNA Gene | 19 | GC19P053 | 1.11236 | https://www.genecards.org/cgi- |
| MIR363 | MicroRNA | RNA Gene | 18 | GC0XM13 | 1.11236 | https://www.genecards.org/cgi- |
| TUBB3 | Tubulin Be | Protein Co | 51 | GC16P091 | 1.098414 | https://www.genecards.org/cgi- |
| HSP90B1 | Heat Shoc | Protein Co | 50 | GC12P103 | 1.098414 | https://www.genecards.org/cgi- |
| SLC2A1 | Solute Car | Protein Co | 56 | GC01M04 | 1.078167 | https://www.genecards.org/cgi- |
| HMOX1 | Heme Oxy | Protein Co | 55 | GC22P035 | 1.078167 | https://www.genecards.org/cgi- |
| PTPN11 | Protein Ty | Protein Co | 55 | GC12P112 | 1.078167 | https://www.genecards.org/cgi- |
| PRKACA | Protein Kir | Protein Co | 54 | GC19M01 | 1.078167 | https://www.genecards.org/cgi- |
| PAK3 | P21 (RAC1 | Protein Co | 53 | GC0XP110 | 1.078167 | https://www.genecards.org/cgi- |
| CD36 | CD36 Mol | Protein Co | 52 | GC07P080 | 1.078167 | https://www.genecards.org/cgi- |
| PIK3R2 | Phosphoir | Protein Co | 52 | GC19P018 | 1.078167 | https://www.genecards.org/cgi- |
| PPP2CA | Protein Ph | Protein Co | 52 | GC05M13 | 1.078167 | https://www.genecards.org/cgi- |
| CASP6 | Caspase 6 | Protein Co | 51 | GC04M10 | 1.078167 | https://www.genecards.org/cgi- |
| CDH3 | Cadherin 3 | Protein Co | 50 | GC16P068 | 1.078167 | https://www.genecards.org/cgi- |
| EEF2K | Eukaryotic | Protein Co | 50 | GC16P022 | 1.078167 | https://www.genecards.org/cgi- |

| | | | | | | |
|---|---|---|---|---|---|---|
| LRP1 | LDL Recep | Protein Co | 50 | GC12P057 | 1.078167 | https://www.genecards.org/cgi-l |
| PAK2 | P21 (RAC1 | Protein Co | 50 | GC03P196 | 1.078167 | https://www.genecards.org/cgi-l |
| PTK6 | Protein Ty | Protein Co | 50 | GC20M063 | 1.078167 | https://www.genecards.org/cgi-l |
| LPAR1 | Lysophosp | Protein Co | 49 | GC09M110 | 1.078167 | https://www.genecards.org/cgi-l |
| TCF7L2 | Transcripti | Protein Co | 49 | GC10P112 | 1.078167 | https://www.genecards.org/cgi-l |
| CEACAM1 | CEA Cell A | Protein Co | 48 | GC19M042 | 1.078167 | https://www.genecards.org/cgi-l |
| GDF2 | Growth Di | Protein Co | 47 | GC10P047 | 1.078167 | https://www.genecards.org/cgi-l |
| GLO1 | Glyoxalase | Protein Co | 47 | GC06M069 | 1.078167 | https://www.genecards.org/cgi-l |
| IL17A | Interleukin | Protein Co | 47 | GC06P052 | 1.078167 | https://www.genecards.org/cgi-l |
| RBL2 | RB Transcr | Protein Co | 47 | GC16P053 | 1.078167 | https://www.genecards.org/cgi-l |
| SIRPA | Signal Reg | Protein Co | 47 | GC20P001 | 1.078167 | https://www.genecards.org/cgi-l |
| TRAF2 | TNF Recep | Protein Co | 47 | GC09P136 | 1.078167 | https://www.genecards.org/cgi-l |
| ADCY10 | Adenylate | Protein Co | 46 | GC01M167 | 1.078167 | https://www.genecards.org/cgi-l |
| VPS37A | VPS37A Su | Protein Co | 45 | GC08P017 | 1.078167 | https://www.genecards.org/cgi-l |
| TNFRSF12A | TNF Recep | Protein Co | 44 | GC16P003 | 1.078167 | https://www.genecards.org/cgi-l |
| APOBEC3C | Apolipopr | Protein Co | 43 | GC22P041 | 1.078167 | https://www.genecards.org/cgi-l |
| BAG1 | BAG Coch | Protein Co | 43 | GC09M033 | 1.078167 | https://www.genecards.org/cgi-l |
| COL13A1 | Collagen T | Protein Co | 43 | GC10P069 | 1.078167 | https://www.genecards.org/cgi-l |
| MNX1 | Motor Neu | Protein Co | 43 | GC07M156 | 1.078167 | https://www.genecards.org/cgi-l |
| RAD9A | RAD9 Che | Protein Co | 42 | GC11P070 | 1.078167 | https://www.genecards.org/cgi-l |
| IFI27 | Interferon | Protein Co | 40 | GC14P094 | 1.078167 | https://www.genecards.org/cgi-l |
| MEGF11 | Multiple E | Protein Co | 40 | GC15M065 | 1.078167 | https://www.genecards.org/cgi-l |
| ITPRIP | Inositol 1,4 | Protein Co | 39 | GC10M104 | 1.078167 | https://www.genecards.org/cgi-l |
| BCL2L15 | BCL2 Like | Protein Co | 34 | GC01M113 | 1.078167 | https://www.genecards.org/cgi-l |
| SNAI2 | Snail Famil | Protein Co | 48 | GC08M048 | 1.050075 | https://www.genecards.org/cgi-l |
| PTPN1 | Protein Ty | Protein Co | 54 | GC20P050 | 1.037607 | https://www.genecards.org/cgi-l |
| NOTCH3 | Notch Rec | Protein Co | 53 | GC19M015 | 1.037607 | https://www.genecards.org/cgi-l |
| GLUD1 | Glutamate | Protein Co | 52 | GC10M087 | 1.037607 | https://www.genecards.org/cgi-l |
| SIRT1 | Sirtuin 1 | Protein Co | 52 | GC10P067 | 1.037607 | https://www.genecards.org/cgi-l |
| FASN | Fatty Acid | Protein Co | 51 | GC17M082 | 1.037607 | https://www.genecards.org/cgi-l |
| MYH9 | Myosin He | Protein Co | 51 | GC22M036 | 1.037607 | https://www.genecards.org/cgi-l |
| RPS6KB1 | Ribosomal | Protein Co | 51 | GC17P059 | 1.037607 | https://www.genecards.org/cgi-l |
| TPM1 | Tropomyo | Protein Co | 51 | GC15P123 | 1.037607 | https://www.genecards.org/cgi-l |
| PPP2R1A | Protein Ph | Protein Co | 50 | GC19P070 | 1.037607 | https://www.genecards.org/cgi-l |
| COL4A2 | Collagen T | Protein Co | 49 | GC13P110 | 1.037607 | https://www.genecards.org/cgi-l |
| CTNND1 | Catenin De | Protein Co | 48 | GC11P058 | 1.037607 | https://www.genecards.org/cgi-l |
| CD151 | CD151 Mo | Protein Co | 47 | GC11P001 | 1.037607 | https://www.genecards.org/cgi-l |
| MMP11 | Matrix Me | Protein Co | 47 | GC22P023 | 1.037607 | https://www.genecards.org/cgi-l |
| ARHGEF7 | Rho Guani | Protein Co | 45 | GC13P111 | 1.037607 | https://www.genecards.org/cgi-l |
| PPP2R2A | Protein Ph | Protein Co | 45 | GC08P026 | 1.037607 | https://www.genecards.org/cgi-l |
| SEMA7A | Semaphor | Protein Co | 45 | GC15M074 | 1.037607 | https://www.genecards.org/cgi-l |
| PPP2R5A | Protein Ph | Protein Co | 43 | GC01P212 | 1.037607 | https://www.genecards.org/cgi-l |
| BST2 | Bone Marr | Protein Co | 42 | GC19M017 | 1.037607 | https://www.genecards.org/cgi-l |
| CCN1 | Cellular Cc | Protein Co | 41 | GC01P085 | 1.037607 | https://www.genecards.org/cgi-l |
| PPP2R2D | Protein Ph | Protein Co | 37 | GC10P131 | 1.037607 | https://www.genecards.org/cgi-l |
| CCDC178 | Coiled-Co | Protein Co | 31 | GC18M032 | 1.037607 | https://www.genecards.org/cgi-l |
| MIR10A | MicroRNA | RNA Gene | 23 | GC17M048 | 1.037607 | https://www.genecards.org/cgi-l |
| MIR30B | MicroRNA | RNA Gene | 23 | GC08M134 | 1.037607 | https://www.genecards.org/cgi-l |
| MIR30C1 | MicroRNA | RNA Gene | 23 | GC01P040 | 1.037607 | https://www.genecards.org/cgi-l |
| SHC1 | SHC Adap | Protein Co | 46 | GC01M154 | 1.016699 | https://www.genecards.org/cgi-l |
| BUB1 | BUB1 Mito | Protein Co | 51 | GC02M110 | 0.984749 | https://www.genecards.org/cgi-l |
| CDC25C | Cell Divisic | Protein Co | 50 | GC05M138 | 0.984749 | https://www.genecards.org/cgi-l |
| CDK1 | Cyclin Dep | Protein Co | 50 | GC10P060 | 0.984749 | https://www.genecards.org/cgi-l |
| ITGB5 | Integrin Su | Protein Co | 50 | GC03M124 | 0.984749 | https://www.genecards.org/cgi-l |
| SETD2 | SET Doma | Protein Co | 50 | GC03M047 | 0.984749 | https://www.genecards.org/cgi-l |
| BUB3 | BUB3 Mito | Protein Co | 49 | GC10P123 | 0.984749 | https://www.genecards.org/cgi-l |
| FER | FER Tyrosi | Protein Co | 48 | GC05P108 | 0.984749 | https://www.genecards.org/cgi-l |
| TP73 | Tumor Prc | Protein Co | 48 | GC01P003 | 0.984749 | https://www.genecards.org/cgi-l |

| | | | | | | |
|---|---|---|---|---|---|---|
| SLCO1B3 | Solute Car | Protein Co | 47 | GC12P020 | 0.984749 | https://www.genecards.org/cgi- |
| TDGF1 | Teratocarc | Protein Co | 47 | GC03P047 | 0.984749 | https://www.genecards.org/cgi- |
| DLG1 | Discs Larg | Protein Co | 46 | GC03M19 | 0.984749 | https://www.genecards.org/cgi- |
| EDAR | Ectodyspla | Protein Co | 46 | GC02M10 | 0.984749 | https://www.genecards.org/cgi- |
| MAD2L1 | Mitotic Ar | Protein Co | 46 | GC04M12 | 0.984749 | https://www.genecards.org/cgi- |
| BCL2L2 | BCL2 Like | Protein Co | 45 | GC14P033 | 0.984749 | https://www.genecards.org/cgi- |
| PDCD6IP | Programm | Protein Co | 45 | GC03P033 | 0.984749 | https://www.genecards.org/cgi- |
| SH3GLB1 | SH3 Doma | Protein Co | 44 | GC01P086 | 0.984749 | https://www.genecards.org/cgi- |
| SCRIB | Scribble Pl | Protein Co | 42 | GC08M14 | 0.984749 | https://www.genecards.org/cgi- |
| DYNLL2 | Dynein Lig | Protein Co | 40 | GC17P058 | 0.984749 | https://www.genecards.org/cgi- |
| TSC2 | TSC Comp | Protein Co | 51 | GC16P012 | 0.958522 | https://www.genecards.org/cgi- |
| BAG4 | BAG Coch | Protein Co | 41 | GC08P038 | 0.920355 | https://www.genecards.org/cgi- |
| MAP3K7 | Mitogen- | Protein Co | 53 | GC06M09 | 0.917962 | https://www.genecards.org/cgi- |
| F10 | Coagulatio | Protein Co | 53 | GC13P113 | 0.857139 | https://www.genecards.org/cgi- |
| F3 | Coagulatio | Protein Co | 48 | GC01M09 | 0.857139 | https://www.genecards.org/cgi- |
| ADAMTSL | ADAMTS L | Protein Co | 45 | GC09P017 | 0.857139 | https://www.genecards.org/cgi- |
| SERPINB1 | Serpin Fan | Protein Co | 43 | GC06M00 | 0.857139 | https://www.genecards.org/cgi- |
| MIR181A1 | MicroRNA | RNA Gene | 20 | GC01M19 | 0.857139 | https://www.genecards.org/cgi- |
| MAP3K1 | Mitogen- | Protein Co | 52 | GC05P056 | 0.83795 | https://www.genecards.org/cgi- |
| CTBP1 | C-Termina | Protein Co | 49 | GC04M00 | 0.83795 | https://www.genecards.org/cgi- |
| CEACAM4 | CEA Cell A | Protein Co | 37 | GC19M06 | 0.803757 | https://www.genecards.org/cgi- |
| PXN | Paxillin | Protein Co | 47 | GC12M12 | 0.791615 | https://www.genecards.org/cgi- |
| MALAT1 | Metastasis | RNA Gene | 26 | GC11P070 | 0.787873 | https://www.genecards.org/cgi- |
| GSTP1 | Glutathion | Protein Co | 53 | GC11P067 | 0.763197 | https://www.genecards.org/cgi- |
| PRDX1 | Peroxiredo | Protein Co | 52 | GC01M04 | 0.763197 | https://www.genecards.org/cgi- |
| IKBKG | Inhibitor C | Protein Co | 51 | GC0XP154 | 0.737493 | https://www.genecards.org/cgi- |
| TFDP1 | Transcripti | Protein Co | 45 | GC13P113 | 0.737493 | https://www.genecards.org/cgi- |
| CRYBA1 | Crystallin | Protein Co | 41 | GC17P029 | 0.737493 | https://www.genecards.org/cgi- |
| SERPINE1 | Serpin Fan | Protein Co | 52 | GC07P101 | 0.730513 | https://www.genecards.org/cgi- |
| FOXO3 | Forkhead | Protein Co | 48 | GC06P108 | 0.729289 | https://www.genecards.org/cgi- |
| ACTG1 | Actin Gam | Protein Co | 51 | GC17M08 | 0.710339 | https://www.genecards.org/cgi- |
| ARHGDIA | Rho GDP | Protein Co | 49 | GC17M08 | 0.710339 | https://www.genecards.org/cgi- |
| EZR | Ezrin | Protein Co | 48 | GC06M15 | 0.710339 | https://www.genecards.org/cgi- |
| SLC39A6 | Solute Car | Protein Co | 42 | GC18M03 | 0.710339 | https://www.genecards.org/cgi- |
| BIN1 | Bridging In | Protein Co | 48 | GC02M12 | 0.700655 | https://www.genecards.org/cgi- |
| TIAM1 | TIAM Rac1 | Protein Co | 47 | GC21M03 | 0.700655 | https://www.genecards.org/cgi- |
| PDPK1 | 3-Phosph | Protein Co | 51 | GC16P002 | 0.69632 | https://www.genecards.org/cgi- |
| SMAD7 | SMAD Fan | Protein Co | 48 | GC18M04 | 0.673421 | https://www.genecards.org/cgi- |
| NTRK3 | Neurotrop | Protein Co | 55 | GC15M08 | 0.643296 | https://www.genecards.org/cgi- |
| RHOC | Ras Homo | Protein Co | 44 | GC01M11 | 0.643296 | https://www.genecards.org/cgi- |
| CASP2 | Caspase 2 | Protein Co | 50 | GC07P148 | 0.635348 | https://www.genecards.org/cgi- |
| TNC | Tenascin C | Protein Co | 52 | GC09M11 | 0.612036 | https://www.genecards.org/cgi- |
| IRF6 | Interferon | Protein Co | 45 | GC01M20 | 0.612036 | https://www.genecards.org/cgi- |
| HOTAIR | HOX Trans | RNA Gene | 26 | GC12M05 | 0.609103 | https://www.genecards.org/cgi- |
| GNE | Glucosami | Protein Co | 44 | GC09M03 | 0.586991 | https://www.genecards.org/cgi- |
| XAF1 | XIAP Asso | Protein Co | 41 | GC17P006 | 0.586991 | https://www.genecards.org/cgi- |
| SFRP1 | Secreted F | Protein Co | 47 | GC08M04 | 0.582729 | https://www.genecards.org/cgi- |
| MAP2K2 | Mitogen- | Protein Co | 56 | GC19M00 | 0.568543 | https://www.genecards.org/cgi- |
| CSK | C-Termina | Protein Co | 50 | GC15P074 | 0.568543 | https://www.genecards.org/cgi- |
| PIK3C2B | Phosphati | Protein Co | 49 | GC01M20 | 0.568543 | https://www.genecards.org/cgi- |
| TAGLN | Transgelin | Protein Co | 46 | GC11P117 | 0.568543 | https://www.genecards.org/cgi- |
| ENDOG | Endonucle | Protein Co | 45 | GC09P128 | 0.568543 | https://www.genecards.org/cgi- |
| FOXC2 | Forkhead | Protein Co | 45 | GC16P086 | 0.568543 | https://www.genecards.org/cgi- |
| RACK1 | Receptor F | Protein Co | 45 | GC05M18 | 0.568543 | https://www.genecards.org/cgi- |
| ARHGDIB | Rho GDP | Protein Co | 44 | GC12M01 | 0.568543 | https://www.genecards.org/cgi- |
| FBLIM1 | Filamin Bir | Protein Co | 40 | GC01P015 | 0.568543 | https://www.genecards.org/cgi- |
| CCDC80 | Coiled-Co | Protein Co | 39 | GC03M11 | 0.568543 | https://www.genecards.org/cgi- |
| PRKD1 | Protein Kir | Protein Co | 52 | GC14M02 | 0.559756 | https://www.genecards.org/cgi- |

| | | | | | | |
|---|---|---|---|---|---|---|
| LDHA | Lactate De | Protein Co | 54 | GC11P018 | 0.529631 | https://www.genecards.org/cgi-l |
| ANXA2 | Annexin A | Protein Co | 51 | GC15M06( | 0.529631 | https://www.genecards.org/cgi-l |
| SPP1 | Secreted F | Protein Co | 49 | GC04P087 | 0.529631 | https://www.genecards.org/cgi-l |
| SMARCE1 | SWI/SNF F | Protein Co | 47 | GC17M04( | 0.529631 | https://www.genecards.org/cgi-l |
| QSOX1 | Quiescin S | Protein Co | 41 | GC01P180 | 0.529631 | https://www.genecards.org/cgi-l |
| RBFOX2 | RNA Bindi | Protein Co | 41 | GC22M03! | 0.529631 | https://www.genecards.org/cgi-l |
| RPS6KA3 | Ribosomal | Protein Co | 55 | GC0XM02( | 0.495438 | https://www.genecards.org/cgi-l |
| CDC42 | Cell Divisic | Protein Co | 53 | GC01P022 | 0.495438 | https://www.genecards.org/cgi-l |
| MAOA | Monoamir | Protein Co | 51 | GC0XP043 | 0.495438 | https://www.genecards.org/cgi-l |
| PIP5K1C | Phosphati( | Protein Co | 51 | GC19M00( | 0.495438 | https://www.genecards.org/cgi-l |
| ATF2 | Activating | Protein Co | 50 | GC02M17! | 0.495438 | https://www.genecards.org/cgi-l |
| JUP | Junction P | Protein Co | 50 | GC17M04: | 0.495438 | https://www.genecards.org/cgi-l |
| NDRG1 | N-Myc Dc | Protein Co | 48 | GC08M13: | 0.495438 | https://www.genecards.org/cgi-l |
| NKX2-1 | NK2 Home | Protein Co | 48 | GC14M03( | 0.495438 | https://www.genecards.org/cgi-l |
| OCLN | Occludin | Protein Co | 47 | GC05P069 | 0.495438 | https://www.genecards.org/cgi-l |
| CRABP2 | Cellular Re | Protein Co | 46 | GC01M15( | 0.495438 | https://www.genecards.org/cgi-l |
| ID2 | Inhibitor C | Protein Co | 46 | GC02P008 | 0.495438 | https://www.genecards.org/cgi-l |
| CEACAM8 | CEA Cell A | Protein Co | 41 | GC19M04: | 0.495438 | https://www.genecards.org/cgi-l |
| PITPNC1 | Phosphati( | Protein Co | 38 | GC17P067 | 0.495438 | https://www.genecards.org/cgi-l |
| AFAP1L1 | Actin Filan | Protein Co | 37 | GC05P149 | 0.495438 | https://www.genecards.org/cgi-l |
| INSR | Insulin Rec | Protein Co | 56 | GC19M00 | 0.454879 | https://www.genecards.org/cgi-l |
| HSPB1 | Heat Shoc | Protein Co | 54 | GC07P076 | 0.454879 | https://www.genecards.org/cgi-l |
| NGF | Nerve Gro | Protein Co | 54 | GC01M11! | 0.454879 | https://www.genecards.org/cgi-l |
| PCNA | Proliferatir | Protein Co | 53 | GC20M00! | 0.454879 | https://www.genecards.org/cgi-l |
| GSK3B | Glycogen ! | Protein Co | 52 | GC03M11! | 0.454879 | https://www.genecards.org/cgi-l |
| TP63 | Tumor Prc | Protein Co | 50 | GC03P189 | 0.454879 | https://www.genecards.org/cgi-l |
| KRT14 | Keratin 14 | Protein Co | 49 | GC17M04: | 0.454879 | https://www.genecards.org/cgi-l |
| SPHK1 | Sphingosir | Protein Co | 49 | GC17P076 | 0.454879 | https://www.genecards.org/cgi-l |
| CTNNA1 | Catenin Al | Protein Co | 48 | GC05P138 | 0.454879 | https://www.genecards.org/cgi-l |
| EHMT2 | Euchromat | Protein Co | 48 | GC06M03: | 0.454879 | https://www.genecards.org/cgi-l |
| OGT | O-Linked | Protein Co | 47 | GC0XP071 | 0.454879 | https://www.genecards.org/cgi-l |
| RAC3 | Rac Family | Protein Co | 47 | GC17P082 | 0.454879 | https://www.genecards.org/cgi-l |
| SIRT6 | Sirtuin 6 | Protein Co | 47 | GC19M00 | 0.454879 | https://www.genecards.org/cgi-l |
| ACP1 | Acid Phos | Protein Co | 45 | GC02P000 | 0.454879 | https://www.genecards.org/cgi-l |
| FOXA1 | Forkhead I | Protein Co | 45 | GC14M03 | 0.454879 | https://www.genecards.org/cgi-l |
| STK38 | Serine/Thr | Protein Co | 45 | GC06M03( | 0.454879 | https://www.genecards.org/cgi-l |
| RHOQ | Ras Homo | Protein Co | 44 | GC02P046 | 0.454879 | https://www.genecards.org/cgi-l |
| ONECUT1 | One Cut H | Protein Co | 43 | GC15M11 | 0.454879 | https://www.genecards.org/cgi-l |
| S100A7 | S100 Calci | Protein Co | 43 | GC01M15: | 0.454879 | https://www.genecards.org/cgi-l |
| SRSF3 | Serine Anc | Protein Co | 43 | GC06P087 | 0.454879 | https://www.genecards.org/cgi-l |
| MUC4 | Mucin 4, C | Protein Co | 42 | GC03M19! | 0.454879 | https://www.genecards.org/cgi-l |
| GKN1 | Gastrokine | Protein Co | 41 | GC02P068 | 0.454879 | https://www.genecards.org/cgi-l |
| MIR107 | MicroRNA | RNA Gene | 22 | GC10M08! | 0.454879 | https://www.genecards.org/cgi-l |
| MIR630 | MicroRNA | RNA Gene | 13 | GC15P072 | 0.454879 | https://www.genecards.org/cgi-l |
| DNMT1 | DNA Meth | Protein Co | 55 | GC19M01( | 0.402021 | https://www.genecards.org/cgi-l |
| LCK | LCK Proto | Protein Co | 55 | GC01P032 | 0.402021 | https://www.genecards.org/cgi-l |
| MERTK | MER Protc | Protein Co | 55 | GC02P111 | 0.402021 | https://www.genecards.org/cgi-l |
| UCHL1 | Ubiquitin ( | Protein Co | 55 | GC04P041 | 0.402021 | https://www.genecards.org/cgi-l |
| CDK2 | Cyclin Dep | Protein Co | 54 | GC12P055 | 0.402021 | https://www.genecards.org/cgi-l |
| MMP3 | Matrix Me | Protein Co | 53 | GC11M10: | 0.402021 | https://www.genecards.org/cgi-l |
| ACTB | Actin Beta | Protein Co | 52 | GC07M00! | 0.402021 | https://www.genecards.org/cgi-l |
| BRCA1 | BRCA1 DN | Protein Co | 52 | GC17M04: | 0.402021 | https://www.genecards.org/cgi-l |
| SLC2A2 | Solute Car | Protein Co | 52 | GC03M17( | 0.402021 | https://www.genecards.org/cgi-l |
| NOS2 | Nitric Oxic | Protein Co | 51 | GC17M02 | 0.402021 | https://www.genecards.org/cgi-l |
| USP9X | Ubiquitin ! | Protein Co | 51 | GC0XP041 | 0.402021 | https://www.genecards.org/cgi-l |
| ROR1 | Receptor T | Protein Co | 50 | GC01P063 | 0.402021 | https://www.genecards.org/cgi-l |
| FYN | FYN Proto | Protein Co | 49 | GC06M11: | 0.402021 | https://www.genecards.org/cgi-l |
| HSPA1A | Heat Shoc | Protein Co | 48 | GC06P087 | 0.402021 | https://www.genecards.org/cgi-l |

| | | | | | | |
|---|---|---|---|---|---|---|
| HTRA2 | HtrA Serin | Protein Co | 48 | GC02P074 | 0.402021 | https://www.genecards.org/cgi-l |
| SNAI1 | Snail Famil | Protein Co | 48 | GC20P049 | 0.402021 | https://www.genecards.org/cgi-l |
| C5AR1 | Compleme | Protein Co | 47 | GC19P047 | 0.402021 | https://www.genecards.org/cgi-l |
| LATS2 | Large Tum | Protein Co | 47 | GC13M020 | 0.402021 | https://www.genecards.org/cgi-l |
| PRDM1 | PR/SET Dc | Protein Co | 47 | GC06P105 | 0.402021 | https://www.genecards.org/cgi-l |
| SKI | SKI Proto- | Protein Co | 47 | GC01P002 | 0.402021 | https://www.genecards.org/cgi-l |
| TPP2 | Tripeptidyl | Protein Co | 47 | GC13P102 | 0.402021 | https://www.genecards.org/cgi-l |
| XRCC5 | X-Ray Rep | Protein Co | 47 | GC02P216 | 0.402021 | https://www.genecards.org/cgi-l |
| CLDN18 | Claudin 18 | Protein Co | 46 | GC03P137 | 0.402021 | https://www.genecards.org/cgi-l |
| SPTA1 | Spectrin A | Protein Co | 46 | GC01M158 | 0.402021 | https://www.genecards.org/cgi-l |
| THY1 | Thy-1 Cell | Protein Co | 46 | GC11M119 | 0.402021 | https://www.genecards.org/cgi-l |
| TJP1 | Tight Junc | Protein Co | 46 | GC15M029 | 0.402021 | https://www.genecards.org/cgi-l |
| CDX2 | Caudal Typ | Protein Co | 45 | GC13M027 | 0.402021 | https://www.genecards.org/cgi-l |
| CENPF | Centromer | Protein Co | 45 | GC01P214 | 0.402021 | https://www.genecards.org/cgi-l |
| DOK2 | Docking P | Protein Co | 45 | GC08M021 | 0.402021 | https://www.genecards.org/cgi-l |
| S100A11 | S100 Calci | Protein Co | 45 | GC01M152 | 0.402021 | https://www.genecards.org/cgi-l |
| SERPINB5 | Serpin Fan | Protein Co | 45 | GC18P063 | 0.402021 | https://www.genecards.org/cgi-l |
| CLIC4 | Chloride Ir | Protein Co | 44 | GC01P024 | 0.402021 | https://www.genecards.org/cgi-l |
| IKZF3 | IKAROS Fa | Protein Co | 44 | GC17M049 | 0.402021 | https://www.genecards.org/cgi-l |
| SNCG | Synuclein | Protein Co | 44 | GC10P093 | 0.402021 | https://www.genecards.org/cgi-l |
| USP11 | Ubiquitin S | Protein Co | 44 | GC0XP047 | 0.402021 | https://www.genecards.org/cgi-l |
| ELAVL1 | ELAV Like | Protein Co | 43 | GC19M007 | 0.402021 | https://www.genecards.org/cgi-l |
| HOXA10 | Homeobo: | Protein Co | 43 | GC07M027 | 0.402021 | https://www.genecards.org/cgi-l |
| LGALS8 | Galectin 8 | Protein Co | 43 | GC01P236 | 0.402021 | https://www.genecards.org/cgi-l |
| SRPX2 | Sushi Repe | Protein Co | 43 | GC0XP100 | 0.402021 | https://www.genecards.org/cgi-l |
| SLPI | Secretory I | Protein Co | 42 | GC20M045 | 0.402021 | https://www.genecards.org/cgi-l |
| HTRA3 | HtrA Serin | Protein Co | 41 | GC04P008 | 0.402021 | https://www.genecards.org/cgi-l |
| EFHD2 | EF-Hand D | Protein Co | 40 | GC01P015 | 0.402021 | https://www.genecards.org/cgi-l |
| IRX1 | Iroquois H | Protein Co | 40 | GC05P003 | 0.402021 | https://www.genecards.org/cgi-l |
| CXCL14 | C-X-C Mo | Protein Co | 39 | GC05M135 | 0.402021 | https://www.genecards.org/cgi-l |
| KIF18A | Kinesin Fa | Protein Co | 38 | GC11M028 | 0.402021 | https://www.genecards.org/cgi-l |
| ZG16B | Zymogen | Protein Co | 37 | GC16P012 | 0.402021 | https://www.genecards.org/cgi-l |
| SBSN | Suprabasir | Protein Co | 35 | GC19M070 | 0.402021 | https://www.genecards.org/cgi-l |
| MIR223 | MicroRNA | RNA Gene | 24 | GC0XP066 | 0.402021 | https://www.genecards.org/cgi-l |
| MIR503 | MicroRNA | RNA Gene | 23 | GC0XM134 | 0.402021 | https://www.genecards.org/cgi-l |
| MIR99A | MicroRNA | RNA Gene | 23 | GC21P016 | 0.402021 | https://www.genecards.org/cgi-l |
| MIR451A | MicroRNA | RNA Gene | 20 | GC17M028 | 0.402021 | https://www.genecards.org/cgi-l |
| MIR7-1 | MicroRNA | RNA Gene | 18 | GC09M095 | 0.402021 | https://www.genecards.org/cgi-l |
| SNORA80I | Small Nucl | RNA Gene | 18 | GC01M155 | 0.402021 | https://www.genecards.org/cgi-l |

bin/carddisp.pl?gene=BRMS1
bin/carddisp.pl?gene=PTK2
bin/carddisp.pl?gene=NTRK2
bin/carddisp.pl?gene=BCL2L11
bin/carddisp.pl?gene=SRC
bin/carddisp.pl?gene=CEACAM6
bin/carddisp.pl?gene=CAV1
bin/carddisp.pl?gene=AKT1
bin/carddisp.pl?gene=ITGB1
bin/carddisp.pl?gene=CEACAM5
bin/carddisp.pl?gene=EGFR
bin/carddisp.pl?gene=BCL2
bin/carddisp.pl?gene=CASP8
bin/carddisp.pl?gene=SIK1
bin/carddisp.pl?gene=PTRH2
bin/carddisp.pl?gene=STAT3
bin/carddisp.pl?gene=TLE1
bin/carddisp.pl?gene=DAPK2
bin/carddisp.pl?gene=CTNNB1
bin/carddisp.pl?gene=ZNF304
bin/carddisp.pl?gene=MAPK1
bin/carddisp.pl?gene=BMF
bin/carddisp.pl?gene=ITGA5
bin/carddisp.pl?gene=TP53
bin/carddisp.pl?gene=MCL1
bin/carddisp.pl?gene=BCL2L1
bin/carddisp.pl?gene=CASP3
bin/carddisp.pl?gene=CDH1
bin/carddisp.pl?gene=BAD
bin/carddisp.pl?gene=PIK3CA
bin/carddisp.pl?gene=PAK1
bin/carddisp.pl?gene=ITGAV
bin/carddisp.pl?gene=FN1
bin/carddisp.pl?gene=MAPK3
bin/carddisp.pl?gene=PTGS2
bin/carddisp.pl?gene=BAX
bin/carddisp.pl?gene=BCAR1
bin/carddisp.pl?gene=PTEN
bin/carddisp.pl?gene=ERBB2
bin/carddisp.pl?gene=ANGPTL4
bin/carddisp.pl?gene=PDK4
bin/carddisp.pl?gene=CYCS
bin/carddisp.pl?gene=BRAF
bin/carddisp.pl?gene=YAP1
bin/carddisp.pl?gene=ANKRD13C
bin/carddisp.pl?gene=ITGA2
bin/carddisp.pl?gene=ANXA5
bin/carddisp.pl?gene=BIRC5
bin/carddisp.pl?gene=MTOR
bin/carddisp.pl?gene=TIMP1
bin/carddisp.pl?gene=BDNF
bin/carddisp.pl?gene=CSPG4
bin/carddisp.pl?gene=BSG
bin/carddisp.pl?gene=AKT2
bin/carddisp.pl?gene=STK11

bin/carddisp.pl?gene=IGF1
bin/carddisp.pl?gene=IGF1R
bin/carddisp.pl?gene=ITGA6
bin/carddisp.pl?gene=ILK
bin/carddisp.pl?gene=CFLAR
bin/carddisp.pl?gene=RHOA
bin/carddisp.pl?gene=HIF1A
bin/carddisp.pl?gene=DAP3
bin/carddisp.pl?gene=MYBBP1A
bin/carddisp.pl?gene=TLE5
bin/carddisp.pl?gene=ITGA3
bin/carddisp.pl?gene=PTK2B
bin/carddisp.pl?gene=CCND1
bin/carddisp.pl?gene=CTTN
bin/carddisp.pl?gene=CALR
bin/carddisp.pl?gene=ATF4
bin/carddisp.pl?gene=CDCP1
bin/carddisp.pl?gene=PLAUR
bin/carddisp.pl?gene=SKP2
bin/carddisp.pl?gene=CHEK2
bin/carddisp.pl?gene=HGF
bin/carddisp.pl?gene=E2F1
bin/carddisp.pl?gene=EGF
bin/carddisp.pl?gene=PIK3CG
bin/carddisp.pl?gene=ITGB4
bin/carddisp.pl?gene=DAPK1
bin/carddisp.pl?gene=MAPK8
bin/carddisp.pl?gene=PIK3R1
bin/carddisp.pl?gene=PIK3R3
bin/carddisp.pl?gene=MAP2K1
bin/carddisp.pl?gene=CXCL12
bin/carddisp.pl?gene=LGALS3
bin/carddisp.pl?gene=FBXW7-AS1
bin/carddisp.pl?gene=BAK1
bin/carddisp.pl?gene=ABHD4
bin/carddisp.pl?gene=CD44
bin/carddisp.pl?gene=ITGA4
bin/carddisp.pl?gene=FADD
bin/carddisp.pl?gene=PHLDA2
bin/carddisp.pl?gene=TGFB1
bin/carddisp.pl?gene=HMCN1
bin/carddisp.pl?gene=MMP2
bin/carddisp.pl?gene=CEBPB
bin/carddisp.pl?gene=CEMIP
bin/carddisp.pl?gene=CDKN3
bin/carddisp.pl?gene=CBL
bin/carddisp.pl?gene=CASP9
bin/carddisp.pl?gene=SFN
bin/carddisp.pl?gene=MTDH
bin/carddisp.pl?gene=PRKCA
bin/carddisp.pl?gene=TNFRSF10B
bin/carddisp.pl?gene=CXCL8
bin/carddisp.pl?gene=MIR200C
bin/carddisp.pl?gene=AR
bin/carddisp.pl?gene=CDKN2A
bin/carddisp.pl?gene=CPT1A
bin/carddisp.pl?gene=PIK3CB
bin/carddisp.pl?gene=CLDN1

bin/carddisp.pl?gene=MIR204
bin/carddisp.pl?gene=MIR26A1
bin/carddisp.pl?gene=CDKN1A
bin/carddisp.pl?gene=CDKN1B
bin/carddisp.pl?gene=KLF12
bin/carddisp.pl?gene=NTRK1
bin/carddisp.pl?gene=PLAU
bin/carddisp.pl?gene=MYC
bin/carddisp.pl?gene=SMAD4
bin/carddisp.pl?gene=PLK1
bin/carddisp.pl?gene=MUC1
bin/carddisp.pl?gene=LGALS1
bin/carddisp.pl?gene=PYCARD
bin/carddisp.pl?gene=SESN2
bin/carddisp.pl?gene=ITGB3
bin/carddisp.pl?gene=KRAS
bin/carddisp.pl?gene=THBS1
bin/carddisp.pl?gene=BID
bin/carddisp.pl?gene=HRAS
bin/carddisp.pl?gene=CDK11B
bin/carddisp.pl?gene=CDK11A
bin/carddisp.pl?gene=XIAP
bin/carddisp.pl?gene=PPARG
bin/carddisp.pl?gene=IL6
bin/carddisp.pl?gene=MIR145
bin/carddisp.pl?gene=CCR7
bin/carddisp.pl?gene=MSLN
bin/carddisp.pl?gene=RAC1
bin/carddisp.pl?gene=GRHL2
bin/carddisp.pl?gene=BIRC3
bin/carddisp.pl?gene=NOTCH1
bin/carddisp.pl?gene=RHOG
bin/carddisp.pl?gene=CCAR2
bin/carddisp.pl?gene=NQO1
bin/carddisp.pl?gene=MMP13
bin/carddisp.pl?gene=FAS
bin/carddisp.pl?gene=MTA1
bin/carddisp.pl?gene=MYO5A
bin/carddisp.pl?gene=EDA2R
bin/carddisp.pl?gene=CCN6
bin/carddisp.pl?gene=MMP9
bin/carddisp.pl?gene=ABL1
bin/carddisp.pl?gene=MAPK11
bin/carddisp.pl?gene=SOD2
bin/carddisp.pl?gene=PTHLH
bin/carddisp.pl?gene=PDGFB
bin/carddisp.pl?gene=GLI2
bin/carddisp.pl?gene=EZH2
bin/carddisp.pl?gene=RIPK1
bin/carddisp.pl?gene=CXCR4
bin/carddisp.pl?gene=HMGA1
bin/carddisp.pl?gene=SIK2
bin/carddisp.pl?gene=TNFSF10
bin/carddisp.pl?gene=ANGPTL2
bin/carddisp.pl?gene=S100A4
bin/carddisp.pl?gene=NTF3
bin/carddisp.pl?gene=ETV4
bin/carddisp.pl?gene=MIR21

bin/carddisp.pl?gene=MIR124-1
bin/carddisp.pl?gene=HTRA1
bin/carddisp.pl?gene=LATS1
bin/carddisp.pl?gene=CEACAM3
bin/carddisp.pl?gene=EIF2AK3
bin/carddisp.pl?gene=LAMC2
bin/carddisp.pl?gene=LAMA3
bin/carddisp.pl?gene=LAMB3
bin/carddisp.pl?gene=CDH2
bin/carddisp.pl?gene=CSNK2A1
bin/carddisp.pl?gene=EDIL3
bin/carddisp.pl?gene=ZEB2
bin/carddisp.pl?gene=TLN1
bin/carddisp.pl?gene=EPHA2
bin/carddisp.pl?gene=SIRT3
bin/carddisp.pl?gene=OLFM3
bin/carddisp.pl?gene=CLU
bin/carddisp.pl?gene=SPINK1
bin/carddisp.pl?gene=CPEB2
bin/carddisp.pl?gene=NAT1
bin/carddisp.pl?gene=TSG101
bin/carddisp.pl?gene=MIR200A
bin/carddisp.pl?gene=MIR6744
bin/carddisp.pl?gene=SERPINA1
bin/carddisp.pl?gene=AKT3
bin/carddisp.pl?gene=RELA
bin/carddisp.pl?gene=TNFRSF1A
bin/carddisp.pl?gene=FASLG
bin/carddisp.pl?gene=AFP
bin/carddisp.pl?gene=ITGA8
bin/carddisp.pl?gene=NOX4
bin/carddisp.pl?gene=PBK
bin/carddisp.pl?gene=SATB1
bin/carddisp.pl?gene=CD63
bin/carddisp.pl?gene=EEF1A1
bin/carddisp.pl?gene=LTB4R2
bin/carddisp.pl?gene=MAVS
bin/carddisp.pl?gene=HRC
bin/carddisp.pl?gene=CCN2
bin/carddisp.pl?gene=RHOB
bin/carddisp.pl?gene=PPP1R13B
bin/carddisp.pl?gene=PLG
bin/carddisp.pl?gene=MET
bin/carddisp.pl?gene=RAF1
bin/carddisp.pl?gene=PARP1
bin/carddisp.pl?gene=PRKCQ
bin/carddisp.pl?gene=BRCA2
bin/carddisp.pl?gene=RB1
bin/carddisp.pl?gene=SP1
bin/carddisp.pl?gene=HAVCR2
bin/carddisp.pl?gene=DOCK1
bin/carddisp.pl?gene=VTN
bin/carddisp.pl?gene=INHBB
bin/carddisp.pl?gene=PDCD4
bin/carddisp.pl?gene=PRPF4B
bin/carddisp.pl?gene=RANBP9
bin/carddisp.pl?gene=SESN1
bin/carddisp.pl?gene=SESN3

bin/carddisp.pl?gene=CD24
bin/carddisp.pl?gene=ZBTB7A
bin/carddisp.pl?gene=MIR141
bin/carddisp.pl?gene=ELANE
bin/carddisp.pl?gene=KDR
bin/carddisp.pl?gene=MDM2
bin/carddisp.pl?gene=NFE2L2
bin/carddisp.pl?gene=ZEB1
bin/carddisp.pl?gene=KL
bin/carddisp.pl?gene=PRKCI
bin/carddisp.pl?gene=CRYAB
bin/carddisp.pl?gene=FGF2
bin/carddisp.pl?gene=HK2
bin/carddisp.pl?gene=LTF
bin/carddisp.pl?gene=IQGAP1
bin/carddisp.pl?gene=MGAT5
bin/carddisp.pl?gene=SDCBP
bin/carddisp.pl?gene=ABHD2
bin/carddisp.pl?gene=SPIB
bin/carddisp.pl?gene=TRIM31
bin/carddisp.pl?gene=MIR1827
bin/carddisp.pl?gene=PDGFRB
bin/carddisp.pl?gene=PLAT
bin/carddisp.pl?gene=TLR3
bin/carddisp.pl?gene=NRAS
bin/carddisp.pl?gene=ROCK1
bin/carddisp.pl?gene=PAK4
bin/carddisp.pl?gene=VEGFA
bin/carddisp.pl?gene=CASP10
bin/carddisp.pl?gene=PIN1
bin/carddisp.pl?gene=IL1RAP
bin/carddisp.pl?gene=UBE2C
bin/carddisp.pl?gene=YWHAZ
bin/carddisp.pl?gene=TWIST1
bin/carddisp.pl?gene=BMP6
bin/carddisp.pl?gene=BNIP3L
bin/carddisp.pl?gene=ELK1
bin/carddisp.pl?gene=KDM3A
bin/carddisp.pl?gene=PRDX4
bin/carddisp.pl?gene=BNIP3
bin/carddisp.pl?gene=LMO3
bin/carddisp.pl?gene=ZNF32
bin/carddisp.pl?gene=MIR200B
bin/carddisp.pl?gene=MIR525
bin/carddisp.pl?gene=MIR363
bin/carddisp.pl?gene=TUBB3
bin/carddisp.pl?gene=HSP90B1
bin/carddisp.pl?gene=SLC2A1
bin/carddisp.pl?gene=HMOX1
bin/carddisp.pl?gene=PTPN11
bin/carddisp.pl?gene=PRKACA
bin/carddisp.pl?gene=PAK3
bin/carddisp.pl?gene=CD36
bin/carddisp.pl?gene=PIK3R2
bin/carddisp.pl?gene=PPP2CA
bin/carddisp.pl?gene=CASP6
bin/carddisp.pl?gene=CDH3
bin/carddisp.pl?gene=EEF2K

bin/carddisp.pl?gene=LRP1
bin/carddisp.pl?gene=PAK2
bin/carddisp.pl?gene=PTK6
bin/carddisp.pl?gene=LPAR1
bin/carddisp.pl?gene=TCF7L2
bin/carddisp.pl?gene=CEACAM1
bin/carddisp.pl?gene=GDF2
bin/carddisp.pl?gene=GLO1
bin/carddisp.pl?gene=IL17A
bin/carddisp.pl?gene=RBL2
bin/carddisp.pl?gene=SIRPA
bin/carddisp.pl?gene=TRAF2
bin/carddisp.pl?gene=ADCY10
bin/carddisp.pl?gene=VPS37A
bin/carddisp.pl?gene=TNFRSF12A
bin/carddisp.pl?gene=APOBEC3G
bin/carddisp.pl?gene=BAG1
bin/carddisp.pl?gene=COL13A1
bin/carddisp.pl?gene=MNX1
bin/carddisp.pl?gene=RAD9A
bin/carddisp.pl?gene=IFI27
bin/carddisp.pl?gene=MEGF11
bin/carddisp.pl?gene=ITPRIP
bin/carddisp.pl?gene=BCL2L15
bin/carddisp.pl?gene=SNAI2
bin/carddisp.pl?gene=PTPN1
bin/carddisp.pl?gene=NOTCH3
bin/carddisp.pl?gene=GLUD1
bin/carddisp.pl?gene=SIRT1
bin/carddisp.pl?gene=FASN
bin/carddisp.pl?gene=MYH9
bin/carddisp.pl?gene=RPS6KB1
bin/carddisp.pl?gene=TPM1
bin/carddisp.pl?gene=PPP2R1A
bin/carddisp.pl?gene=COL4A2
bin/carddisp.pl?gene=CTNND1
bin/carddisp.pl?gene=CD151
bin/carddisp.pl?gene=MMP11
bin/carddisp.pl?gene=ARHGEF7
bin/carddisp.pl?gene=PPP2R2A
bin/carddisp.pl?gene=SEMA7A
bin/carddisp.pl?gene=PPP2R5A
bin/carddisp.pl?gene=BST2
bin/carddisp.pl?gene=CCN1
bin/carddisp.pl?gene=PPP2R2D
bin/carddisp.pl?gene=CCDC178
bin/carddisp.pl?gene=MIR10A
bin/carddisp.pl?gene=MIR30B
bin/carddisp.pl?gene=MIR30C1
bin/carddisp.pl?gene=SHC1
bin/carddisp.pl?gene=BUB1
bin/carddisp.pl?gene=CDC25C
bin/carddisp.pl?gene=CDK1
bin/carddisp.pl?gene=ITGB5
bin/carddisp.pl?gene=SETD2
bin/carddisp.pl?gene=BUB3
bin/carddisp.pl?gene=FER
bin/carddisp.pl?gene=TP73

bin/carddisp.pl?gene=SLCO1B3
bin/carddisp.pl?gene=TDGF1
bin/carddisp.pl?gene=DLG1
bin/carddisp.pl?gene=EDAR
bin/carddisp.pl?gene=MAD2L1
bin/carddisp.pl?gene=BCL2L2
bin/carddisp.pl?gene=PDCD6IP
bin/carddisp.pl?gene=SH3GLB1
bin/carddisp.pl?gene=SCRIB
bin/carddisp.pl?gene=DYNLL2
bin/carddisp.pl?gene=TSC2
bin/carddisp.pl?gene=BAG4
bin/carddisp.pl?gene=MAP3K7
bin/carddisp.pl?gene=F10
bin/carddisp.pl?gene=F3
bin/carddisp.pl?gene=ADAMTSL1
bin/carddisp.pl?gene=SERPINB1
bin/carddisp.pl?gene=MIR181A1
bin/carddisp.pl?gene=MAP3K1
bin/carddisp.pl?gene=CTBP1
bin/carddisp.pl?gene=CEACAM4
bin/carddisp.pl?gene=PXN
bin/carddisp.pl?gene=MALAT1
bin/carddisp.pl?gene=GSTP1
bin/carddisp.pl?gene=PRDX1
bin/carddisp.pl?gene=IKBKG
bin/carddisp.pl?gene=TFDP1
bin/carddisp.pl?gene=CRYBA1
bin/carddisp.pl?gene=SERPINE1
bin/carddisp.pl?gene=FOXO3
bin/carddisp.pl?gene=ACTG1
bin/carddisp.pl?gene=ARHGDIA
bin/carddisp.pl?gene=EZR
bin/carddisp.pl?gene=SLC39A6
bin/carddisp.pl?gene=BIN1
bin/carddisp.pl?gene=TIAM1
bin/carddisp.pl?gene=PDPK1
bin/carddisp.pl?gene=SMAD7
bin/carddisp.pl?gene=NTRK3
bin/carddisp.pl?gene=RHOC
bin/carddisp.pl?gene=CASP2
bin/carddisp.pl?gene=TNC
bin/carddisp.pl?gene=IRF6
bin/carddisp.pl?gene=HOTAIR
bin/carddisp.pl?gene=GNE
bin/carddisp.pl?gene=XAF1
bin/carddisp.pl?gene=SFRP1
bin/carddisp.pl?gene=MAP2K2
bin/carddisp.pl?gene=CSK
bin/carddisp.pl?gene=PIK3C2B
bin/carddisp.pl?gene=TAGLN
bin/carddisp.pl?gene=ENDOG
bin/carddisp.pl?gene=FOXC2
bin/carddisp.pl?gene=RACK1
bin/carddisp.pl?gene=ARHGDIB
bin/carddisp.pl?gene=FBLIM1
bin/carddisp.pl?gene=CCDC80
bin/carddisp.pl?gene=PRKD1

bin/carddisp.pl?gene=LDHA
bin/carddisp.pl?gene=ANXA2
bin/carddisp.pl?gene=SPP1
bin/carddisp.pl?gene=SMARCE1
bin/carddisp.pl?gene=QSOX1
bin/carddisp.pl?gene=RBFOX2
bin/carddisp.pl?gene=RPS6KA3
bin/carddisp.pl?gene=CDC42
bin/carddisp.pl?gene=MAOA
bin/carddisp.pl?gene=PIP5K1C
bin/carddisp.pl?gene=ATF2
bin/carddisp.pl?gene=JUP
bin/carddisp.pl?gene=NDRG1
bin/carddisp.pl?gene=NKX2-1
bin/carddisp.pl?gene=OCLN
bin/carddisp.pl?gene=CRABP2
bin/carddisp.pl?gene=ID2
bin/carddisp.pl?gene=CEACAM8
bin/carddisp.pl?gene=PITPNC1
bin/carddisp.pl?gene=AFAP1L1
bin/carddisp.pl?gene=INSR
bin/carddisp.pl?gene=HSPB1
bin/carddisp.pl?gene=NGF
bin/carddisp.pl?gene=PCNA
bin/carddisp.pl?gene=GSK3B
bin/carddisp.pl?gene=TP63
bin/carddisp.pl?gene=KRT14
bin/carddisp.pl?gene=SPHK1
bin/carddisp.pl?gene=CTNNA1
bin/carddisp.pl?gene=EHMT2
bin/carddisp.pl?gene=OGT
bin/carddisp.pl?gene=RAC3
bin/carddisp.pl?gene=SIRT6
bin/carddisp.pl?gene=ACP1
bin/carddisp.pl?gene=FOXA1
bin/carddisp.pl?gene=STK38
bin/carddisp.pl?gene=RHOQ
bin/carddisp.pl?gene=ONECUT1
bin/carddisp.pl?gene=S100A7
bin/carddisp.pl?gene=SRSF3
bin/carddisp.pl?gene=MUC4
bin/carddisp.pl?gene=GKN1
bin/carddisp.pl?gene=MIR107
bin/carddisp.pl?gene=MIR630
bin/carddisp.pl?gene=DNMT1
bin/carddisp.pl?gene=LCK
bin/carddisp.pl?gene=MERTK
bin/carddisp.pl?gene=UCHL1
bin/carddisp.pl?gene=CDK2
bin/carddisp.pl?gene=MMP3
bin/carddisp.pl?gene=ACTB
bin/carddisp.pl?gene=BRCA1
bin/carddisp.pl?gene=SLC2A2
bin/carddisp.pl?gene=NOS2
bin/carddisp.pl?gene=USP9X
bin/carddisp.pl?gene=ROR1
bin/carddisp.pl?gene=FYN
bin/carddisp.pl?gene=HSPA1A

bin/carddisp.pl?gene=HTRA2
bin/carddisp.pl?gene=SNAI1
bin/carddisp.pl?gene=C5AR1
bin/carddisp.pl?gene=LATS2
bin/carddisp.pl?gene=PRDM1
bin/carddisp.pl?gene=SKI
bin/carddisp.pl?gene=TPP2
bin/carddisp.pl?gene=XRCC5
bin/carddisp.pl?gene=CLDN18
bin/carddisp.pl?gene=SPTA1
bin/carddisp.pl?gene=THY1
bin/carddisp.pl?gene=TJP1
bin/carddisp.pl?gene=CDX2
bin/carddisp.pl?gene=CENPF
bin/carddisp.pl?gene=DOK2
bin/carddisp.pl?gene=S100A11
bin/carddisp.pl?gene=SERPINB5
bin/carddisp.pl?gene=CLIC4
bin/carddisp.pl?gene=IKZF3
bin/carddisp.pl?gene=SNCG
bin/carddisp.pl?gene=USP11
bin/carddisp.pl?gene=ELAVL1
bin/carddisp.pl?gene=HOXA10
bin/carddisp.pl?gene=LGALS8
bin/carddisp.pl?gene=SRPX2
bin/carddisp.pl?gene=SLPI
bin/carddisp.pl?gene=HTRA3
bin/carddisp.pl?gene=EFHD2
bin/carddisp.pl?gene=IRX1
bin/carddisp.pl?gene=CXCL14
bin/carddisp.pl?gene=KIF18A
bin/carddisp.pl?gene=ZG16B
bin/carddisp.pl?gene=SBSN
bin/carddisp.pl?gene=MIR223
bin/carddisp.pl?gene=MIR503
bin/carddisp.pl?gene=MIR99A
bin/carddisp.pl?gene=MIR451A
bin/carddisp.pl?gene=MIR7-1
bin/carddisp.pl?gene=SNORA80E
