# Supplementary material for: System analysis based on Anoikis-related genes identifies MAPK1 as a novel therapy target for osteosarcoma with neoadjuvant chemotherapy
Source: BMC Musculoskelet Disord. 2024 Jun 4;25:437. doi: 10.1186/s12891-024-07547-2 (PMC11149263; doi:10.1186/s12891-024-07547-2)
Supplement: Supplementary file 2 — Supplementary Material 2 [file 12891_2024_7547_MOESM2_ESM.pdf]

Anoikis-related gene lists from Harmonizome portals

BMF	Bcl2 modifying factor
DAPK2	death-associated protein kinase 2
E2F1	E2F transcription factor 1
STK11	serine/threonine kinase 11
TFDP1	transcription factor Dp-1
ABHD4	abhydrolase domain containing 4
AFAP1L1	actin filament associated protein 1-like 1
AKT1	v-akt murine thymoma viral oncogene homolog 1
AKT2	v-akt murine thymoma viral oncogene homolog 2
ANGPTL4	angiopoietin-like 4
BAX	BCL2-associated X protein
BCAR1	breast cancer anti-estrogen resistance 1

BCL2	B-cell CLL/lymphoma 2
BCL2L11	BCL2-like 11 (apoptosis facilitator)
BIRC3	baculoviral IAP repeat containing 3
BMF	Bcl2 modifying factor
BRAF	B-Raf proto-oncogene, serine/threonine kinase
BRCA2	breast cancer 2, early onset
BSG	basigin (Ok blood group)
CALR	calreticulin
CASP2	caspase 2, apoptosis-related cysteine peptidase
CASP3	caspase 3, apoptosis-related cysteine peptidase
CASP8	caspase 8, apoptosis-related cysteine peptidase

CAV1	caveolin 1, caveolae protein, 22kDa
CCAR2	cell cycle and apoptosis regulator 2
CD63	CD63 molecule
CDCP1	CUB domain containing protein 1
CDH1	cadherin 1, type 1, E-cadherin (epithelial)
CDH2	cadherin 2, type 1, N-cadherin (neuronal)
CDKN2A	cyclin-dependent kinase inhibitor 2A
CEACAM6	carcinoembryonic antigen-related cell adhesion molecule 6 (non-specific cross reacting antigen)
CEBPB	CCAAT/enhancer binding protein (C/EBP), beta
CHUK	conserved helix-loop-helix ubiquitous kinase

CLDN1	claudin 1
CLU	clusterin
CMA1	chymase 1, mast cell
COPS5	COP9 signalosome subunit 5
CSNK2A1	casein kinase 2, alpha 1 polypeptide
CSPG4	chondroitin sulfate proteoglycan 4
CTNND1	catenin (cadherin-associated protein), delta 1
CTTN	cortactin
CXCL12	chemokine (C-X-C motif) ligand 12
DAP3	death associated protein 3
DAPK1	death-associated protein kinase 1
DLG1	discs, large homolog 1 (Drosophila)
EDA2R	ectodysplasin A2 receptor
EEF1A1	eukaryotic translation elongation factor 1 alpha 1

EEF2K	eukaryotic elongation factor 2 kinase
EGFR	epidermal growth factor receptor
EIF2AK3	eukaryotic translation initiation factor 2-alpha kinase 3
ERBB4	erb-b2 receptor tyrosine kinase 4
FER	fer (fps/fes related) tyrosine kinase
FGF2	fibroblast growth factor 2 (basic)
FN1	fibronectin 1
HGF	hepatocyte growth factor (hepapoietin A; scatter factor)
HK2	hexokinase 2
HMCN1	hemicentin 1
HMGA1	high mobility group AT-hook 1
HOXA10	homeobox A10
HTRA1	HtrA serine peptidase 1

IGF1R	insulin-like growth factor 1 receptor
IKZF3	IKAROS family zinc finger 3 (Aiolos)
ITGA2	integrin, alpha 2 (CD49B, alpha 2 subunit of VLA-2 receptor)
ITGA3	integrin, alpha 3 (antigen CD49C, alpha 3 subunit of VLA-3 receptor)
ITGA4	integrin, alpha 4 (antigen CD49D, alpha 4 subunit of VLA-4 receptor)
ITGA5	integrin, alpha 5 (fibronectin receptor, alpha polypeptide)
ITGA6	integrin, alpha 6
ITGA8	integrin, alpha 8
ITGAV	integrin, alpha V

ITGB1	integrin, beta 1 (fibronectin receptor, beta polypeptide, antigen CD29 includes MDF2, MSK12)
KDR	kinase insert domain receptor
KL	klotho
KRAS	Kirsten rat sarcoma viral oncogene homolog
LGALS1	lectin, galactoside-binding, soluble, 1
LRP1	low density lipoprotein receptor-related protein 1
LTB4R2	leukotriene B4 receptor 2
MAPK1	mitogen-activated protein kinase 1
MAPK3	mitogen-activated protein kinase 3
MAVS	mitochondrial antiviral signaling protein
MCL1	myeloid cell leukemia 1

MDM2	MDM2 proto-oncogene, E3 ubiquitin protein ligase
MET	MET proto-oncogene, receptor tyrosine kinase
MGAT5	mannosyl (alpha-1,6-)-glycoprotein beta-1,6-N-acetyl-glucosaminyltransferase
MIR200C	microRNA 200c
MMP11	matrix metallopeptidase 11
MMP13	matrix metallopeptidase 13
MMP2	matrix metallopeptidase 2
MTA1	metastasis associated 1
MTOR	mechanistic target of rapamycin (serine/threonine kinase)
MYBBP1A	MYB binding protein (P160) 1a
NRP1	neuropilin 1
NTF3	neurotrophin 3

NTRK2	neurotrophic tyrosine kinase, receptor, type 2
OLFM3	olfactomedin 3
PAK1	p21 protein (Cdc42/Rac)-activated kinase 1
PAK4	p21 protein (Cdc42/Rac)-activated kinase 4
PECAM1	platelet/endothelial cell adhesion molecule 1
PIK3CA	phosphatidylinositol-4,5-bisphosphate 3-kinase, catalytic subunit alpha
PIK3CG	phosphatidylinositol-4,5-bisphosphate 3-kinase, catalytic subunit gamma
PLK1	polo-like kinase 1
PRKCA	protein kinase C, alpha
PRKD1	protein kinase D1
PTEN	phosphatase and tensin homolog

PTHLH	parathyroid hormone-like hormone
PTK2	protein tyrosine kinase 2
PTK2B	protein tyrosine kinase 2 beta
PTK6	protein tyrosine kinase 6
PTPN11	protein tyrosine phosphatase, non-receptor type 11
PTRH2	peptidyl-tRNA hydrolase 2
RAD9A	RAD9 homolog A (S. pombe)
RHOA	ras homolog family member A
RHOC	ras homolog family member C
RIPK1	receptor (TNFRSF)-interacting serine-threonine kinase 1
ROCK1	Rho-associated, coiled-coil containing protein kinase 1

S100A4	S100 calcium binding protein A4
SCRIB	scribbled planar cell polarity protein
SH3GLB1	SH3-domain GRB2-like endophilin B1
SIK1	salt-inducible kinase 1
SIRPA	signal-regulatory protein alpha
SIRT3	sirtuin 3
SKP2	S-phase kinase-associated protein 2, E3 ubiquitin protein ligase
SLCO1B3	solute carrier organic anion transporter family, member 1B3
SMAD4	SMAD family member 4
SNAI2	snail family zinc finger 2
SRC	SRC proto-oncogene, non-receptor tyrosine kinase

STAT3	signal transducer and activator of transcription 3 (acute-phase response factor)
STK11	serine/threonine kinase 11
TAGLN	transgelin
TGFB1	transforming growth factor, beta 1
THBS1	thrombospondin 1
TIMP1	TIMP metallopeptidase inhibitor 1
TP53	tumor protein p53
TPM1	tropomyosin 1 (alpha)
UCHL1	ubiquitin carboxyl-terminal esterase L1 (ubiquitin thiolesterase)
USP9X	ubiquitin specific peptidase 9, X-linked
WISP3	WNT1 inducible signaling pathway protein 3

WNT2	wingless-type MMTV integration site family member 2
XIAP	X-linked inhibitor of apoptosis, E3 ubiquitin protein ligase
YWHAZ	tyrosine 3-monooxygenase/tryptophan 5-monooxygenase activation protein, zeta
