# Supplementary figures and images for: System analysis based on Anoikis-related genes identifies MAPK1 as a novel therapy target for osteosarcoma with neoadjuvant chemotherapy
Source: BMC Musculoskelet Disord. 2024 Jun 4;25:437. doi: 10.1186/s12891-024-07547-2 (PMC11149263; doi:10.1186/s12891-024-07547-2)

**Fig 9**  
GAPDH

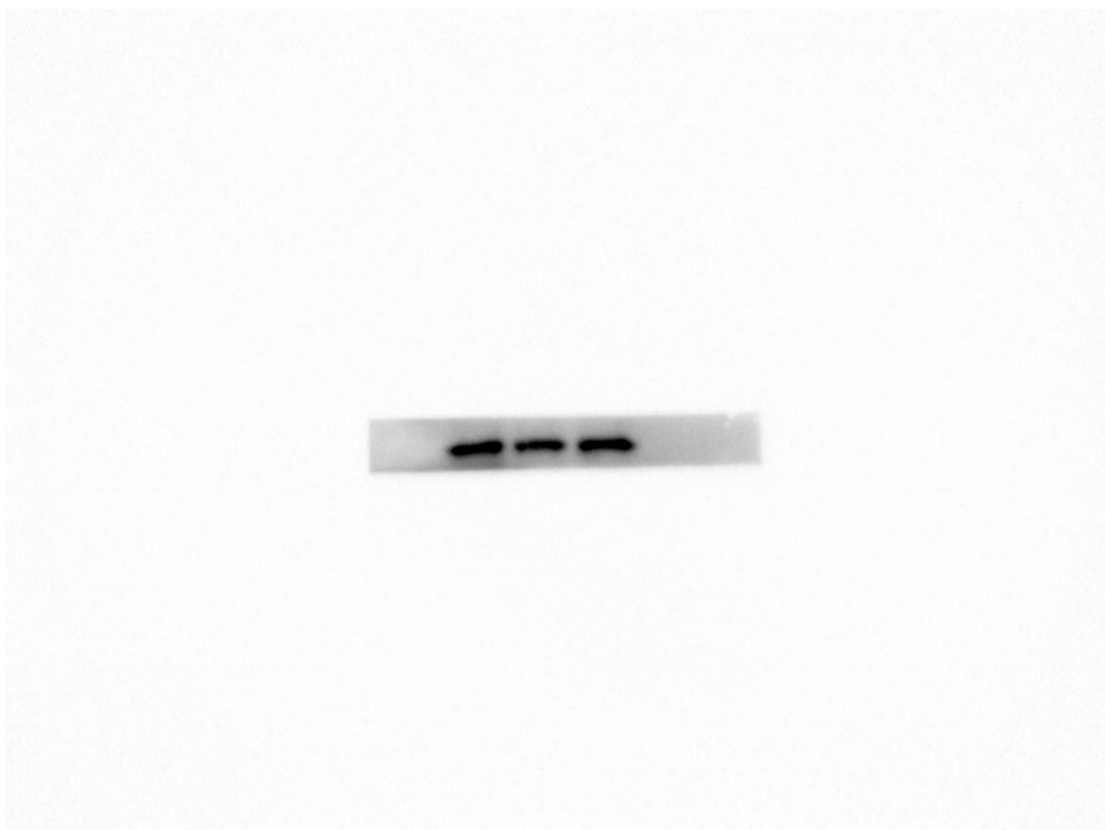

MAPK1

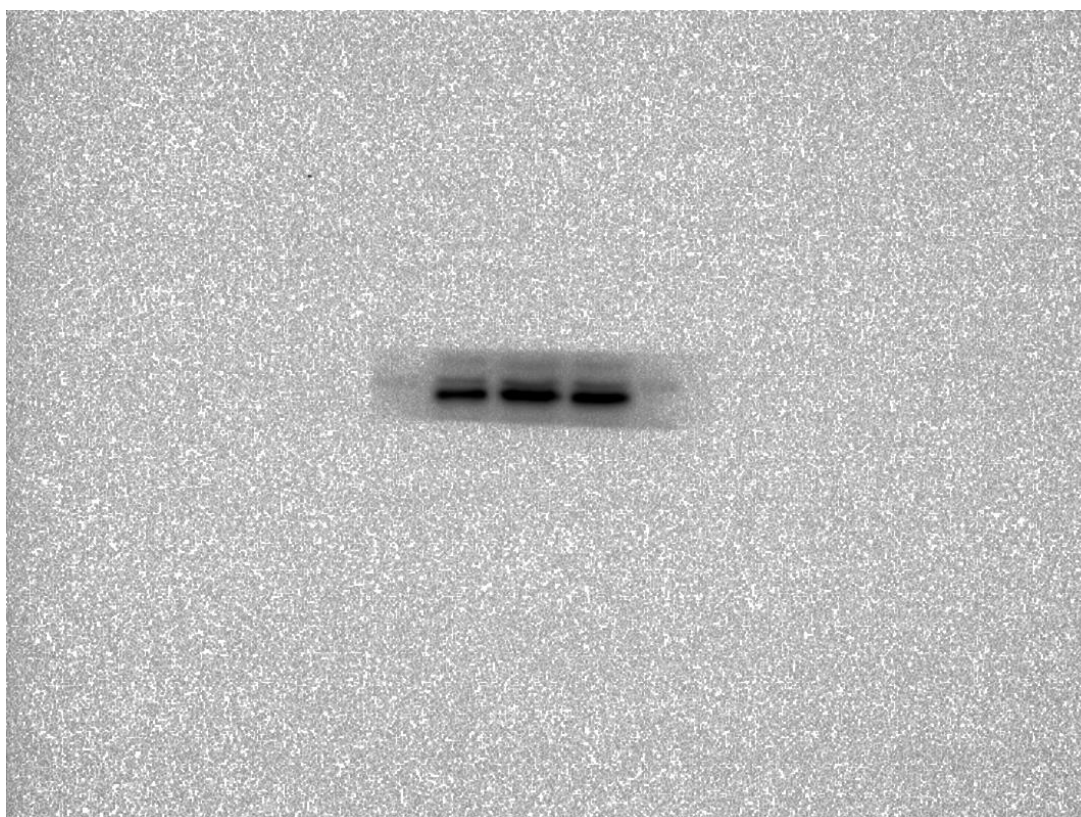

MYC

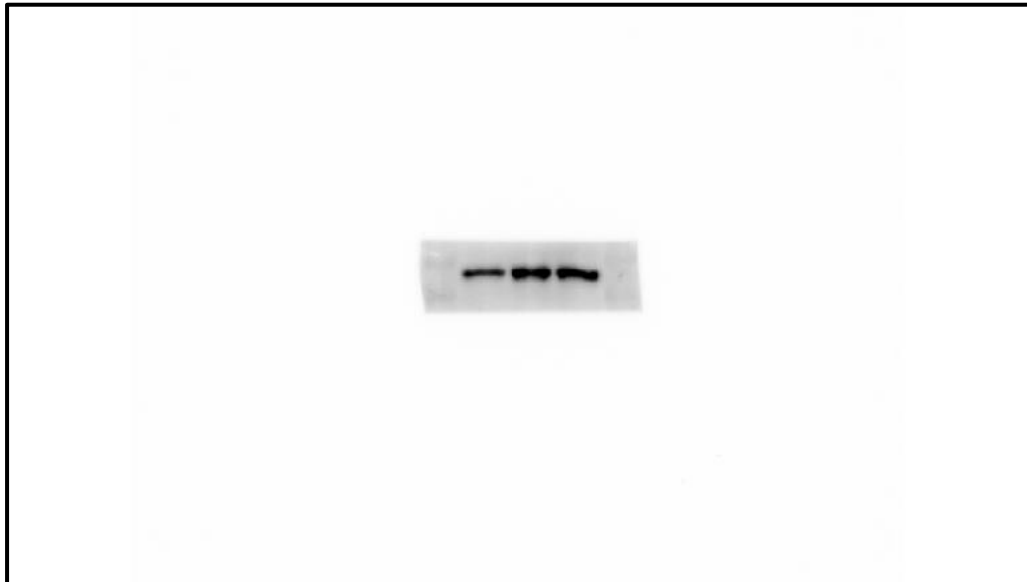

**Fig10**  
GAPDH

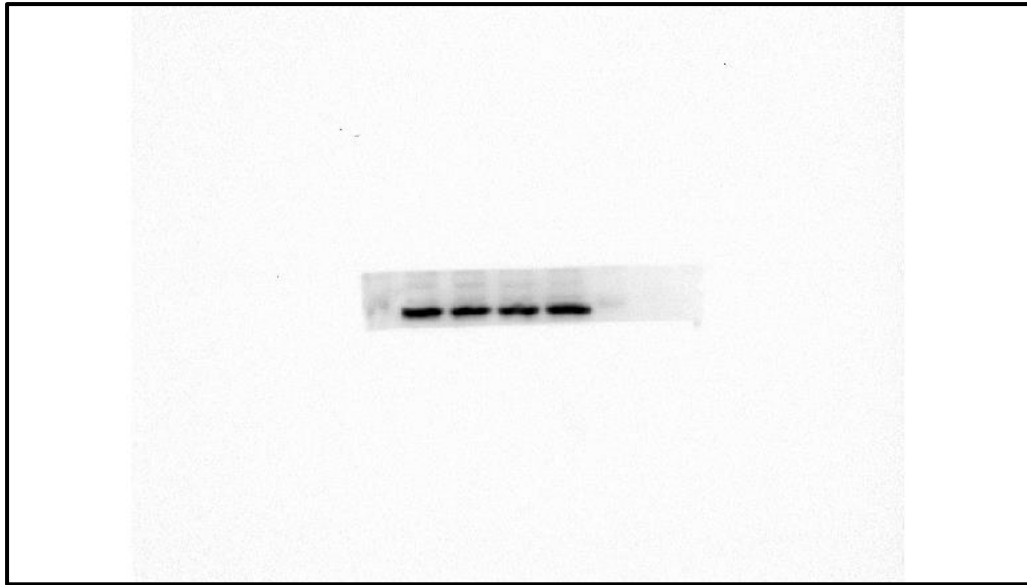

MAPK1

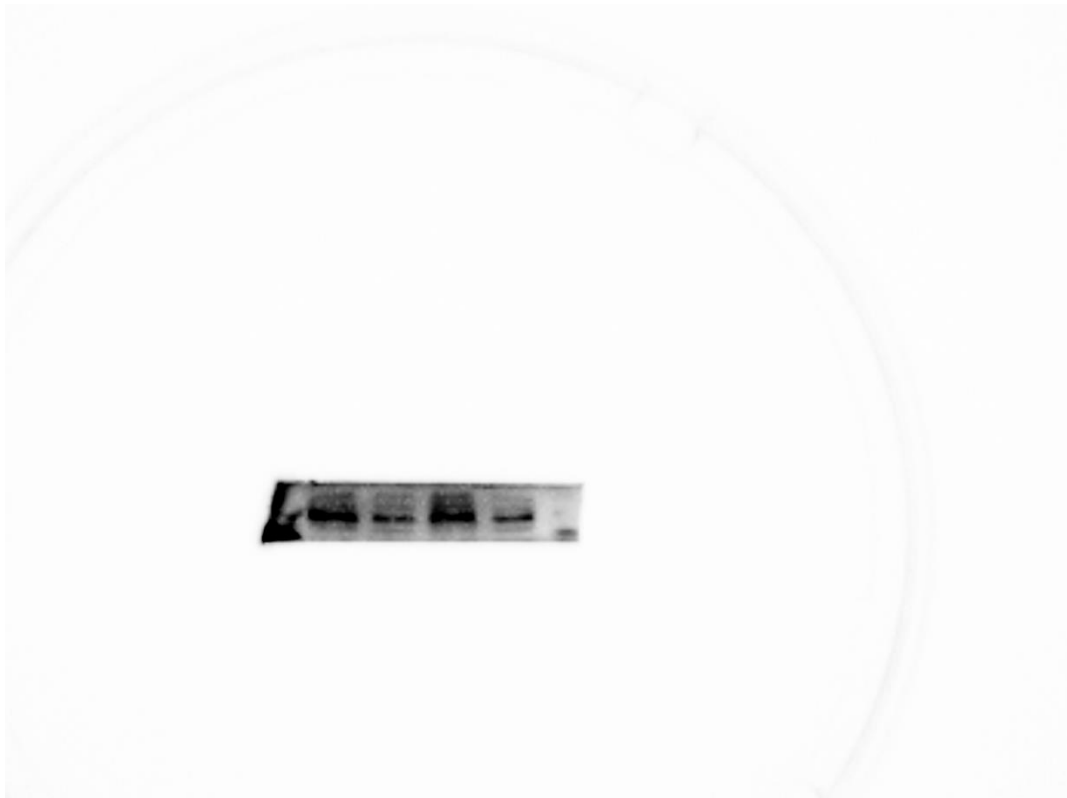

Fig11

BAX

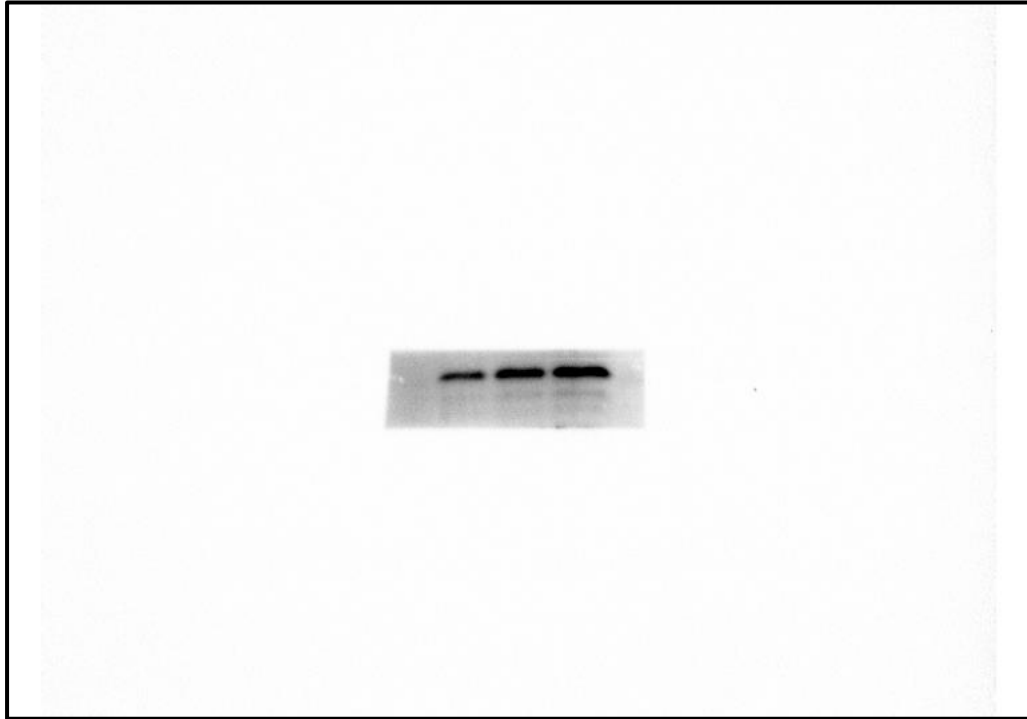

BCL2

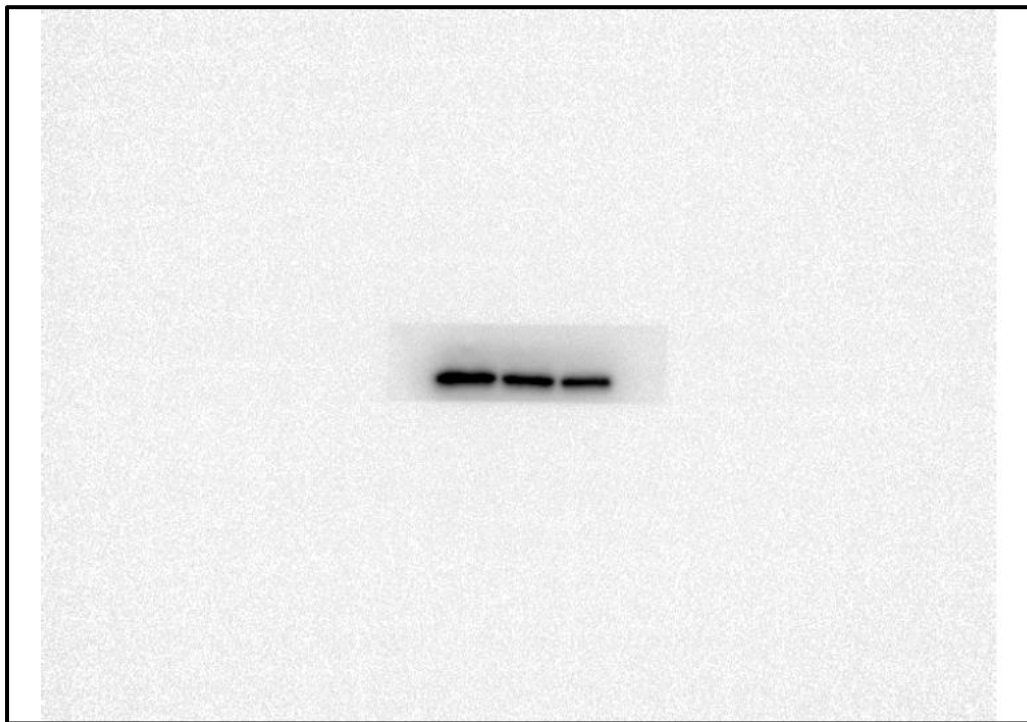

CASP3

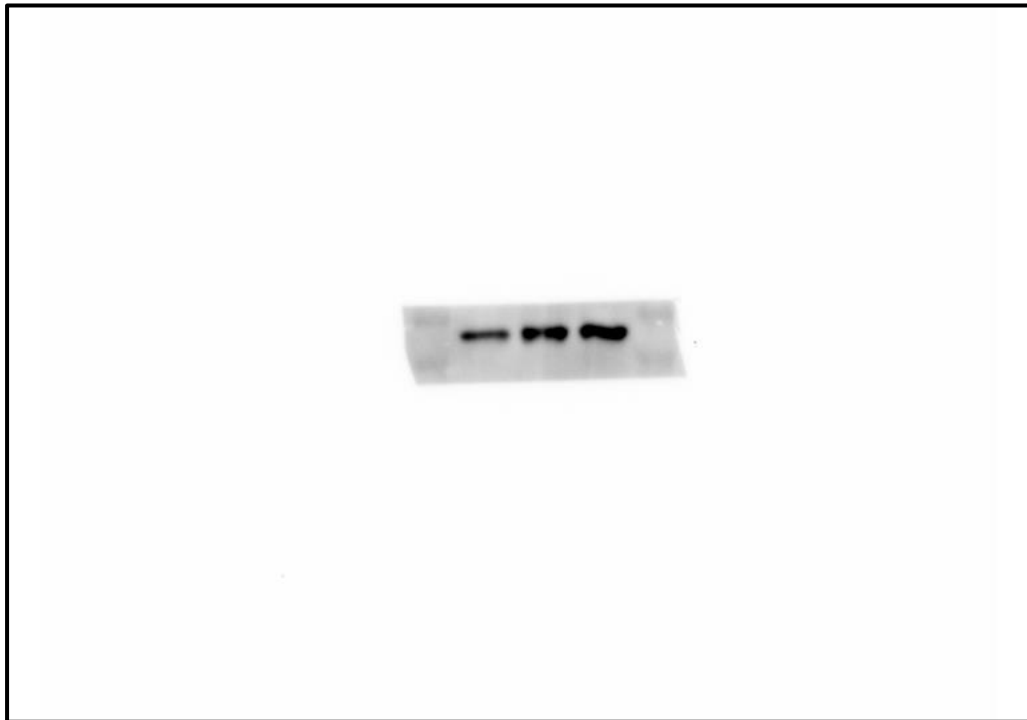

GAPDH

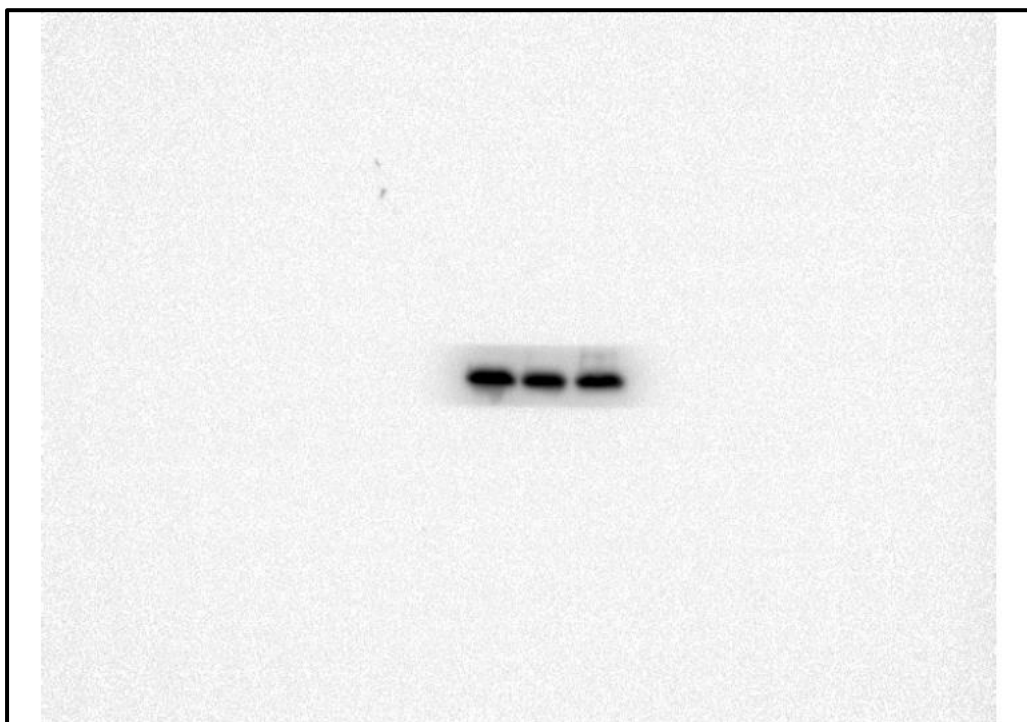

Tunel

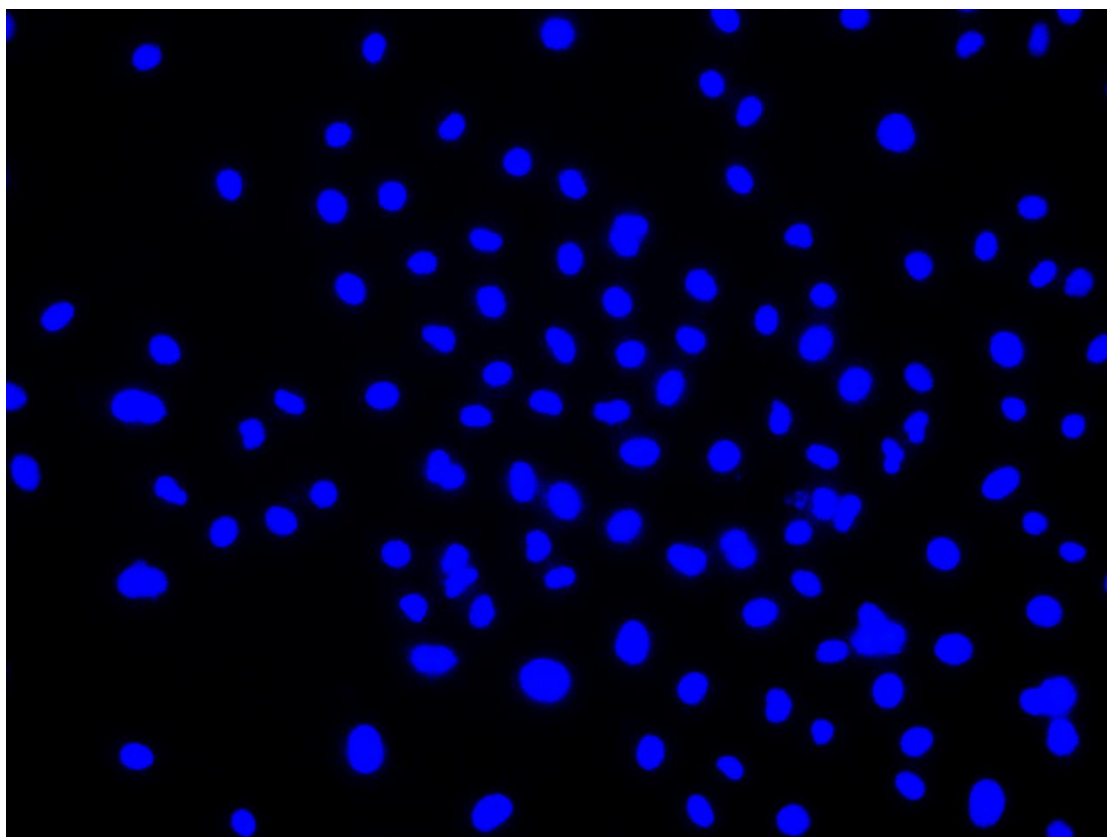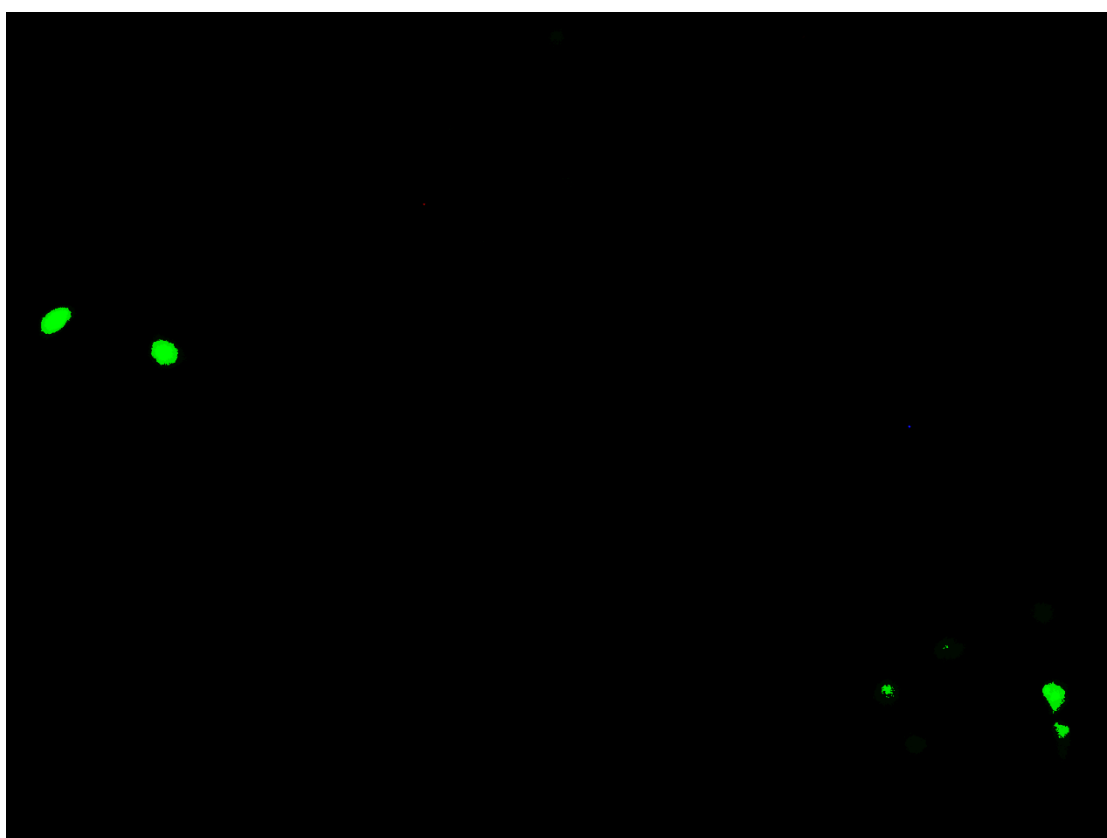

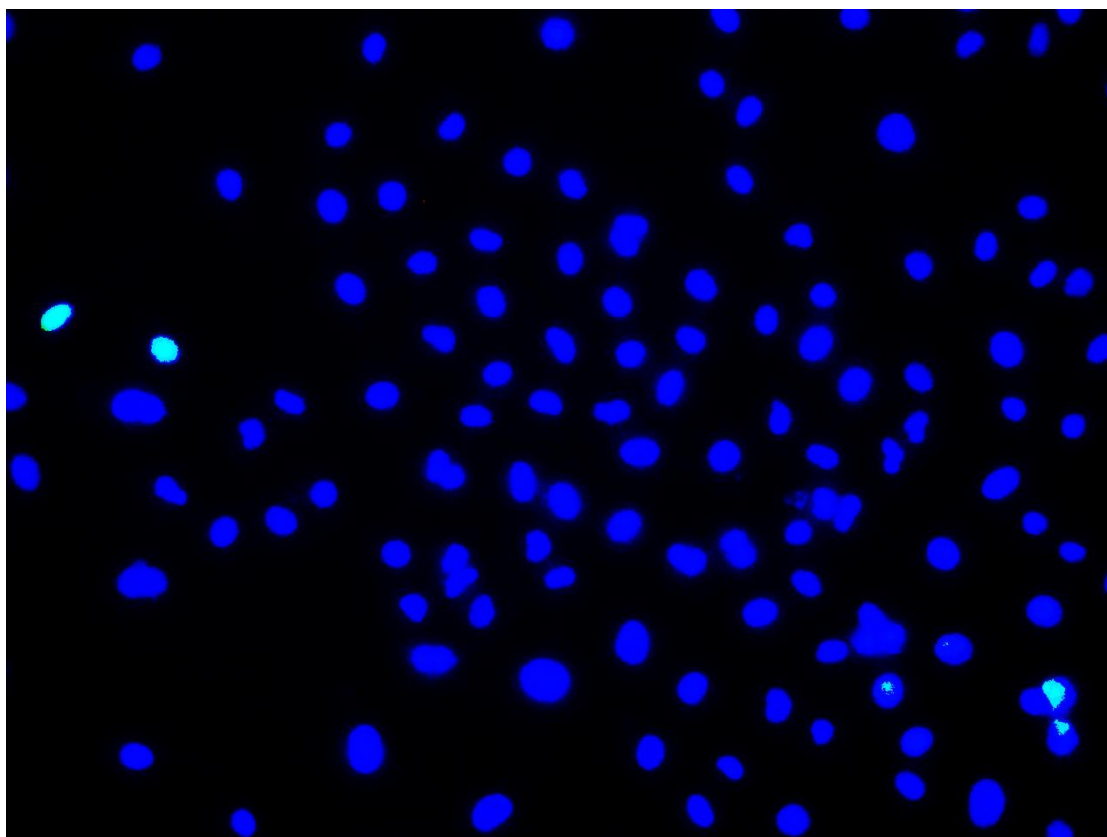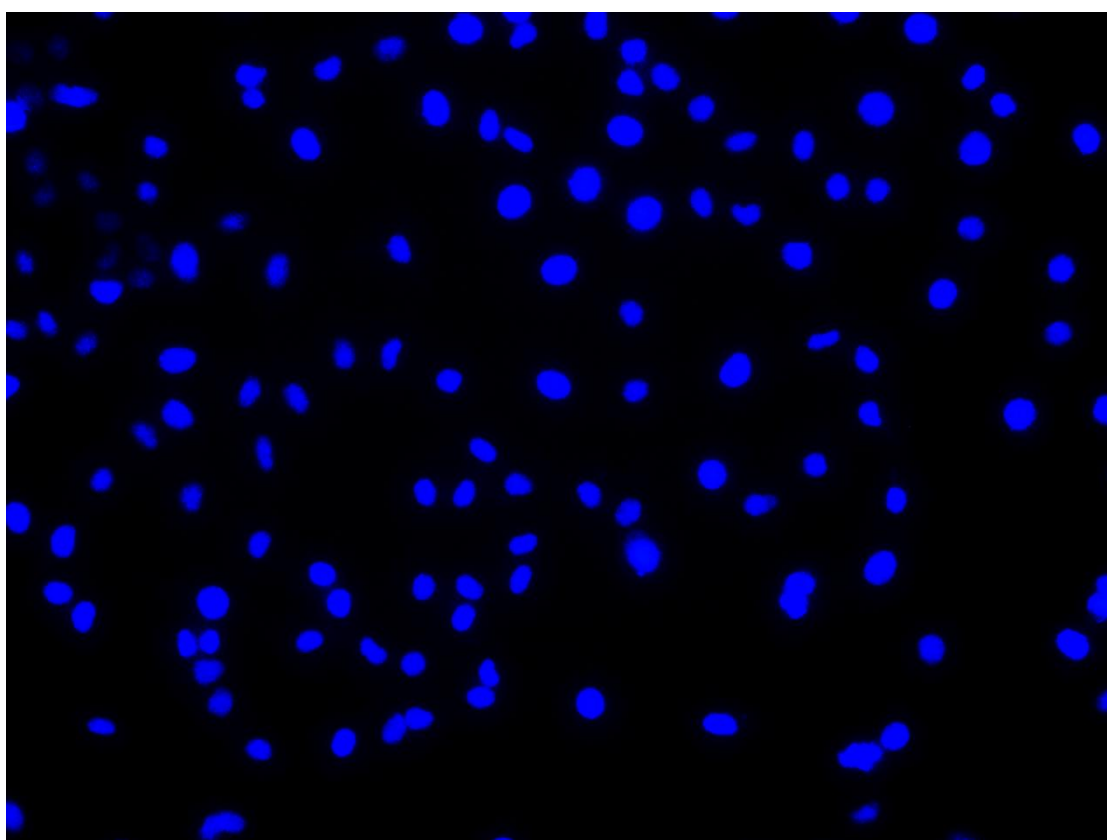

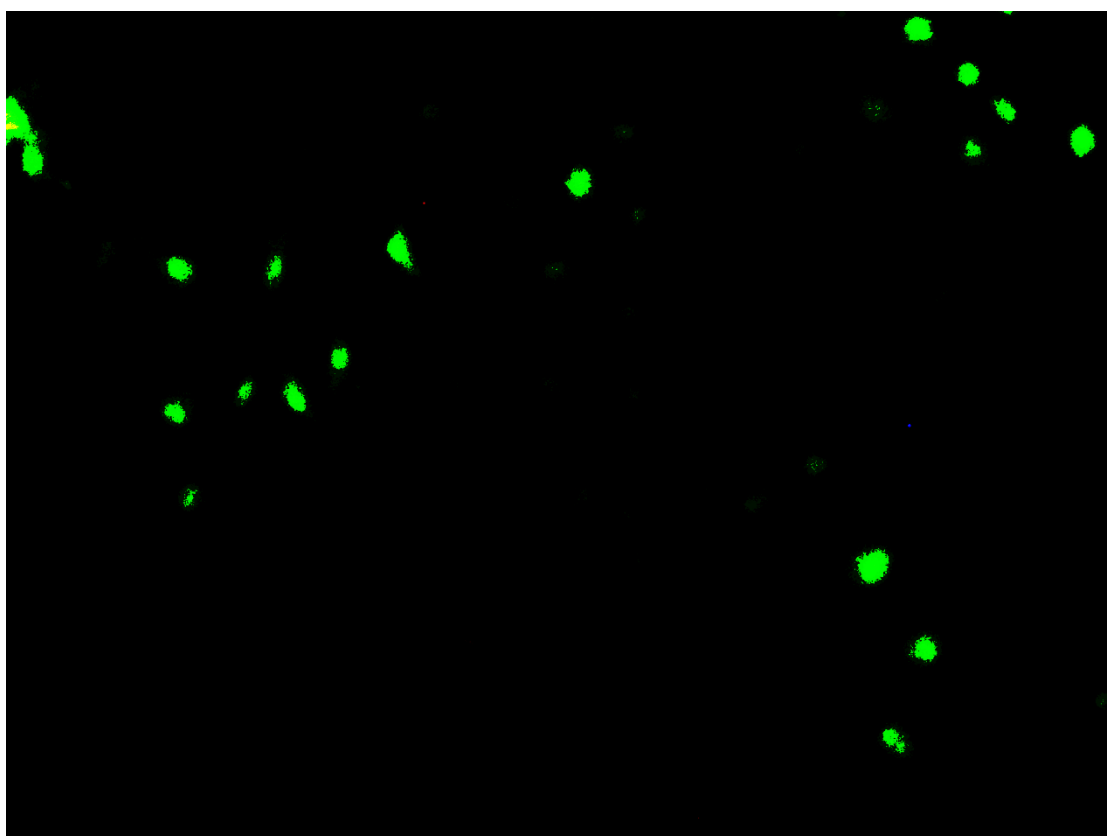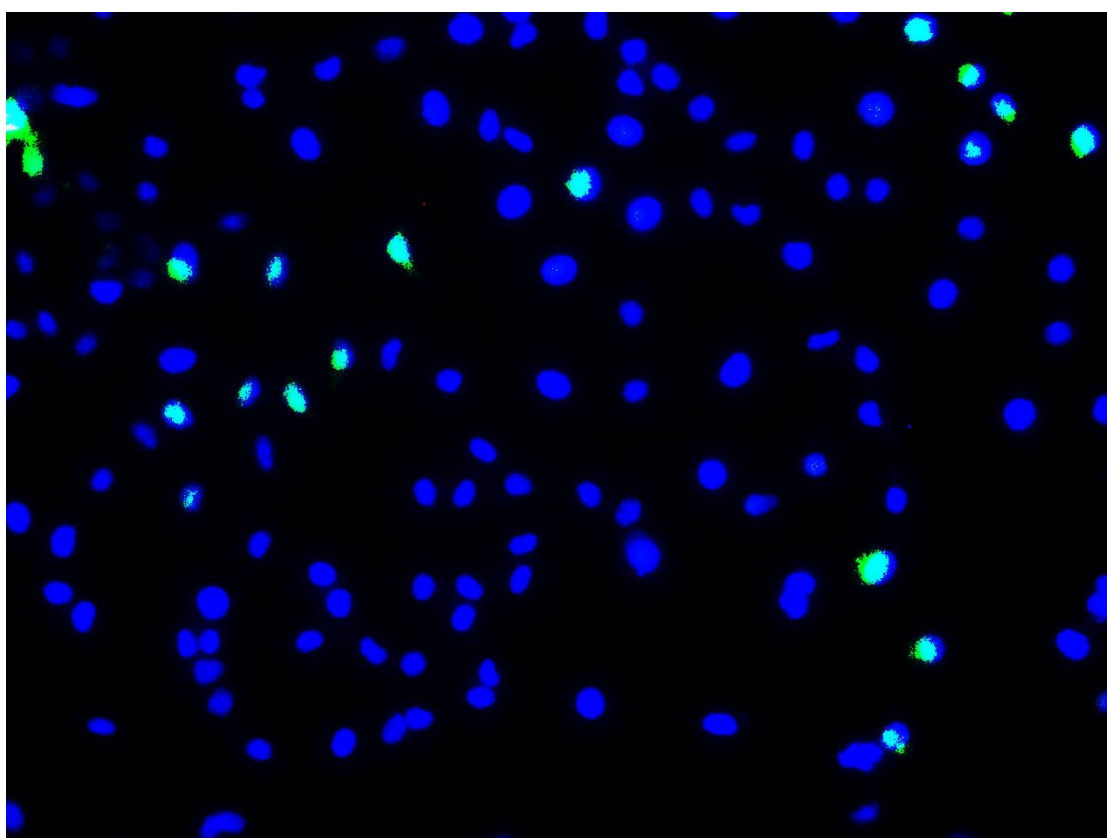

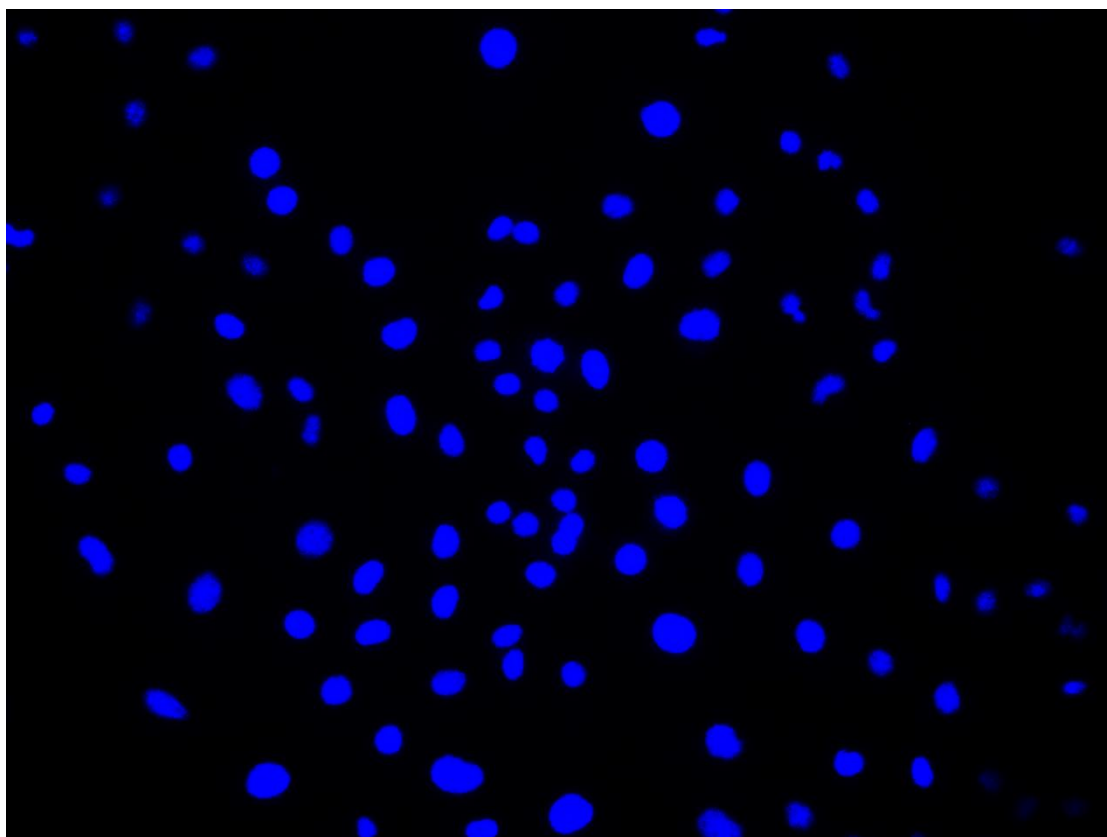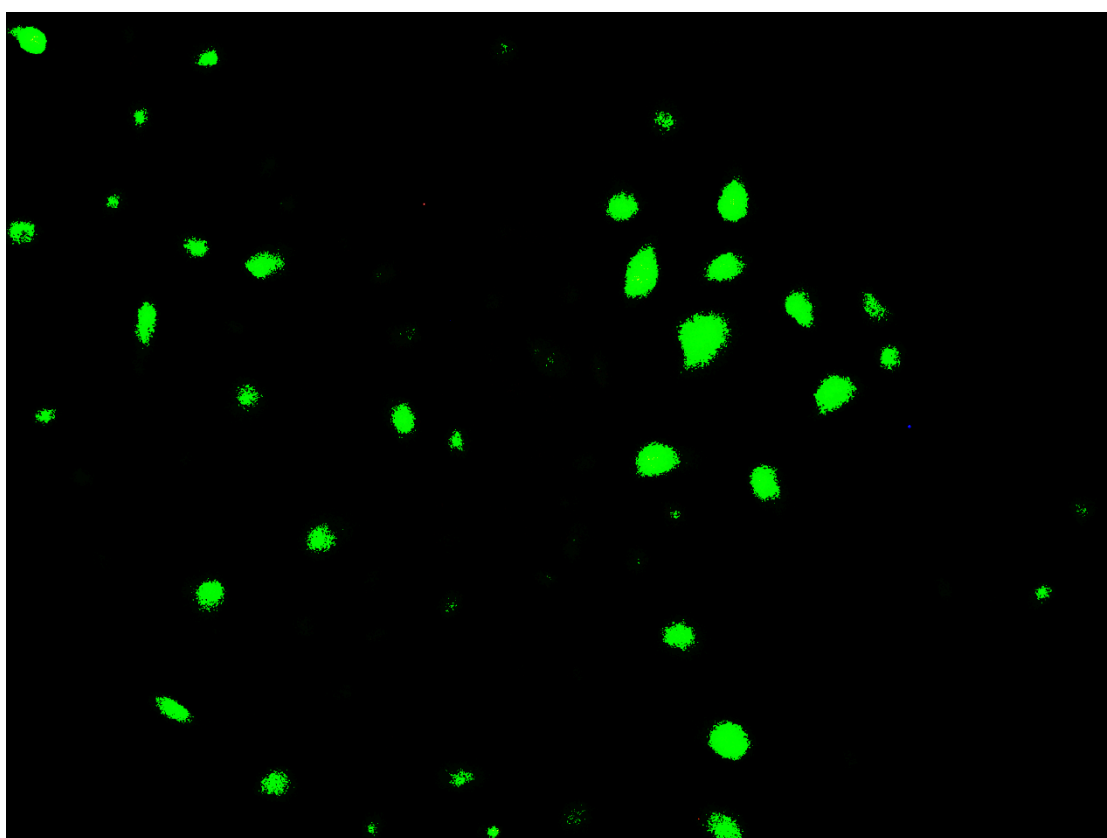

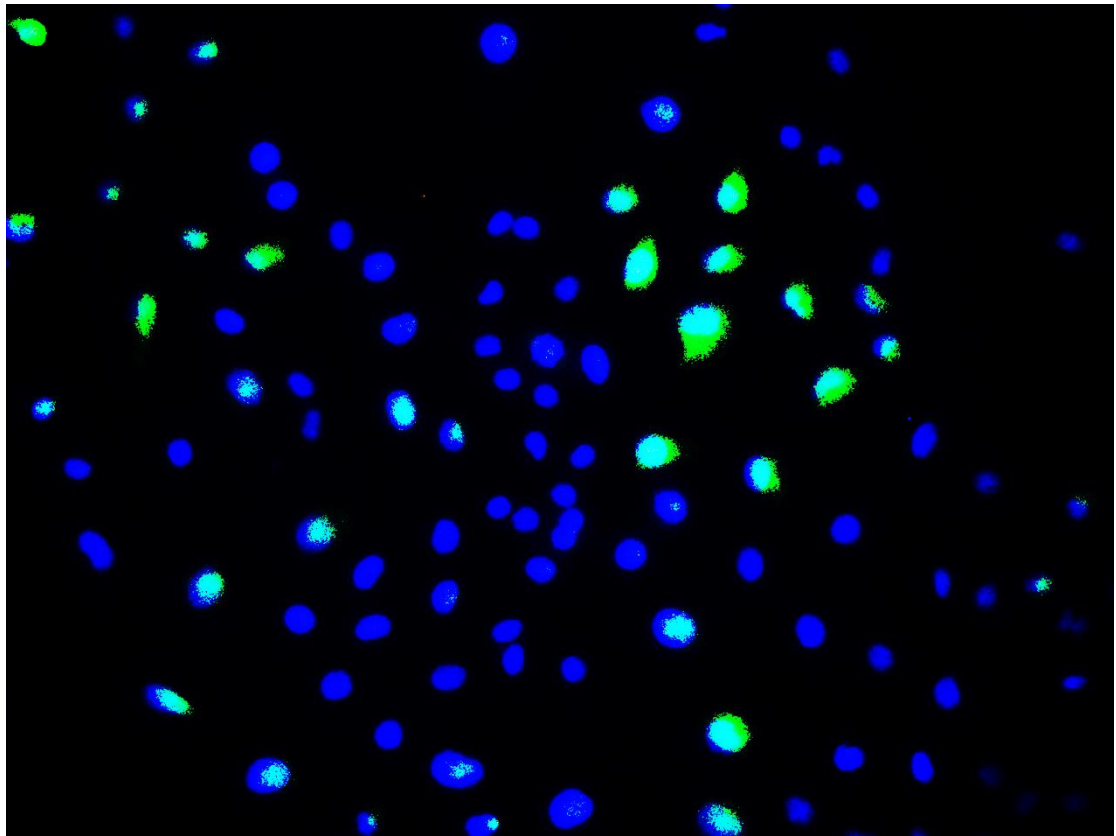

Supplement: Supplementary file 3 — Supplementary Material 3 [file 12891_2024_7547_MOESM3_ESM.pdf]
